# Influence of water deficit on the molecular responses of *Pinus contorta* × *Pinus banksiana* mature trees to infection by the mountain pine beetle fungal associate, *Grosmannia clavigera*

**DOI:** 10.1093/treephys/tpt101

**Published:** 2013-12-05

**Authors:** Adriana Arango-Velez, Leonardo M. Galindo González, Miranda J. Meents, Walid El Kayal, Barry J. Cooke, Jean Linsky, Inka Lusebrink, Janice E.K. Cooke

**Affiliations:** 1 Department of Biological Sciences, University of Alberta, CW405 Biological Sciences Building, Edmonton, AB, Canada T6G 2E9; 2 Natural Resources Canada, Canadian Forest Service, Northern Forestry Centre, Edmonton, AB, Canada T6H 3S5; 3 Department of Renewable Resources, University of Alberta, Edmonton, AB, Canada T6E 2E3

**Keywords:** aquaporin, blue stain fungi, chitinase, *Dendroctonus ponderosae*, DREB, drought, jack pine, lodgepole pine, naïve host, range expansion, systemic, terpene synthase

## Abstract

Conifers exhibit a number of constitutive and induced mechanisms to defend against attack by pests and pathogens such as mountain pine beetle (*Dendroctonus ponderosae* Hopkins) and their fungal associates. Ecological studies have demonstrated that stressed trees are more susceptible to attack by mountain pine beetle than their healthy counterparts. In this study, we tested the hypothesis that water deficit affects constitutive and induced responses of mature lodgepole pine × jack pine hybrids (*Pinus contorta* Dougl. ex Loud. var. *latifolia* Engelm. ex S. Wats. × *Pinus banksiana* Lamb.) to inoculation with the mountain pine beetle fungal associate *Grosmannia clavigera* (Robinson-Jeffrey and Davidson) Zipfel, de Beer and Wingfield. The degree of stress induced by the imposed water-deficit treatment was sufficient to reduce photosynthesis. *Grosmannia clavigera*-induced lesions exhibited significantly reduced dimensions in water-deficit trees relative to well-watered trees at 5 weeks after inoculation. Treatment-associated cellular-level changes in secondary phloem were also observed. Quantitative RT-PCR was used to analyze transcript abundance profiles of 18 genes belonging to four families classically associated with biotic and abiotic stress responses: aquaporins (*AQPs*), dehydration-responsive element binding (*DREB*), terpene synthases (*TPSs*) and chitinases (*CHIs*). Transcript abundance profiles of a *TIP2 AQP* and a *TINY-like DREB* decreased significantly in fungus-inoculated trees, but not in response to water deficit. One TPS, *Pcb(+)-3-carene synthase*, and the Class II CHIs *PcbCHI2.1* and *PcbCHI2.2* showed increased expression under water-deficit conditions in the absence of fungal inoculation, while another TPS, *Pcb(E)-*β*-farnesene synthase-like*, and two CHIs, *PcbCHI1.1* and *PcbCHI4.1*, showed attenuated expression under water-deficit conditions in the presence of fungal inoculation. The effects were observed both locally and systemically. These results demonstrate that both constitutive and induced carbon- and nitrogen-based defenses are affected by water deficit, suggesting potential consequences for mountain pine beetle dynamics, particularly in novel environments.

## Introduction

The current outbreak of mountain pine beetle (MPB; *Dendroctonus ponderasae* Hopkins) has resulted in the loss of more than 28 million hectares of pine forest in western North America since 1999, including more than 19 million hectares in the western Canadian provinces of British Columbia and Alberta (Government of Alberta 2012, Government of British Columbia 2012, Man 2012). In 2006, the MPB outbreak spread to the lodgepole pine (*Pinus contorta* Dougl. ex Loud. var. *latifolia* Engelm. ex S. Wats.) × jack pine (*Pinus banksiana* Lamb.) hybrid zone in north-central Alberta, and more recently has undergone a host-shift expansion into pure jack pine, a boreal forest species with a range extending east to the Atlantic coast (Cullingham et al. 2011). This unprecedented expansion of MPB into more northerly latitudes, into higher elevation forests and east into jack pine forests—all formerly considered marginal habitats for MPB—is postulated to have been facilitated at least in part by warmer than average temperatures during recent decades (Carroll et al. 2004, Trenberth et al. 2007).

Mountain pine beetles overcome host defenses through a mass-attack strategy, i.e., concerted attack of a tree by an aggregation of insects, overcoming the critical threshold of resistance for physiologically stressed trees at lower attack densities than that of healthy and vigorously growing trees (Berryman 1982, Kolb et al. 1998, Wallin and Raffa 2002, Raffa et al. 2005, Boone et al. 2011). It is surmised that larger, healthier trees provide greater resources to MPB and their fungal pathogen associates, enabling greater fecundity and offspring survival (Raffa et al. 2008). Several fungal species belonging to the Ophiostomaceae are associated with MPB, facilitating an MPB attack and subsequent colonization of the host. *Grosmannia clavigera* (Robinson-Jeffrey and Davidson) Zipfel, de Beer and Wingfield (formerly *Ophiostoma clavigerum*) is considered to be the most pathogenic of the characterized MPB fungal associates, and even in the absence of the MPB is able to overcome tree defenses (Yamaoka et al. 1995, Lee et al. 2006, Safranyik and Carroll 2006). *Grosmannia clavigera* grows in the phloem and sapwood of the host tree (Parmeter et al. 1992), inducing water and mineral deficits that decrease the tree's overall vigor, including the ability to synthesize and mobilize secondary compounds. Fungal growth eventually disrupts water transport via tracheid occlusion, resulting in tree mortality (Christiansen et al. 1987, Yamaoka et al. 1995, Huber et al. 2004).

Trees defend against attack by pests and pathogens via a suite of constitutive and inducible defense mechanisms that include physical, cellular, biochemical and molecular processes (Keeling and Bohlmann 2006, Van Loon et al. 2006, Eyles et al. 2010). In response to bark invaders such as MPB and *G. clavigera*, pine species produce a reaction zone called a lesion in both phloem and xylem in an attempt to contain the invading organism (Hudgins et al. 2003, Franceschi et al. 2005). This lesion contains necrotic cells near the point of invasion, as well as cells containing quantities of defensive compounds (Franceschi et al. 2005). Conifer chemical defenses include carbon-based metabolites such as terpenoids and phenolics that create chemical and physical barriers to attackers (Byun-McKay et al. 2003, 2006, Fäldt et al. 2003, Miller et al. 2005, Keeling and Bohlmann 2006, Van Loon et al. 2006). Terpenoids, a major component of the oleoresins that are a cornerstone of the conifer defense response, are synthesized by a complex network of reactions that include steps catalyzed by terpene synthases (TPSs). Terpene synthases can be further classified as mono-, sesqui- and diterpene synthases (Byun-McKay et al. 2006, Zulak and Bohlmann 2010). Mono- and diterpene synthases producing many of the resin terpenoids and volatiles of lodgepole pine and jack pine have recently been described (Hall et al. 2013*a*, 2013*b*). Protein, i.e., nitrogen-based, defenses also appear to figure prominently in the conifer defense response (Lippert et al. 2007). Chitinases (CHIs) are a well-characterized class of defense proteins in both angiosperms (Bishop et al. 2000) and conifers (Davis et al. 2002, Fossdal et al. 2005). Chitinases that exhibit chitinolytic activity hydrolyze chitin, a cell wall constituent of many fungi, and may also be involved in generating signal molecules that function as endogenous elicitors of other defense mechanisms (Bishop et al. 2000, Van Loon et al. 2006, Miranda et al. 2007). Members of the *CHI* gene family also function in non-chitinolytic roles in plants, such as antifreeze proteins and vegetative storage proteins (Islam et al. 2011).

Water deficit is an abiotic stress that affects tree defenses (Dunn and Lorio 1993, Franceschi et al. 2005, McDowell et al. 2008). Lombardero et al. (2000) have demonstrated that constitutive oleoresin flow was increased in water-stressed loblolly pine (*Pinus taeda* L.) relative to unstressed trees, but wound-induced resin flow was decreased. Lusebrink et al. (2011) demonstrated that both constitutive and *G. clavigera*-induced monoterpene volatile profiles of both lodgepole pine and jack pine seedlings are affected by water deficit. Northern Alberta has experienced periods of drought in the past two decades (Hanesiak et al. 2011) that have had measureable negative impacts on these forests (Chhin et al. 2008, Michaelian et al. 2011). If the drought experienced in the region has adversely impacted constitutive and induced defense responses of pines, the critical threshold for a successful MPB attack may be lowered. Given that MPB population densities are comparatively low at the leading edges of an outbreak, factors that serve to reduce the attack threshold at which MPB successfully overcome host tree defenses could increase the probability of MPB establishment in novel habitats, facilitating continued range expansion (Safranyik and Carroll 2006, Raffa et al. 2008).

In this study, we have examined the effect of water limitation on molecular defense responses of mature lodgepole × jack pine hybrid trees in central Alberta to the MPB fungal associate *G. clavigera.* These hybrids are considered ‘naïve’ hosts, as it is thought that the region has not experienced an MPB outbreak in the past (Safranyik et al. 2010). We hypothesized that water deficit would lead to reduced photosynthesis, concomitantly reducing carbon gain and the availability of photosynthate for allocation to defenses. The degree of water stress imposed by the water limitation treatment was determined by measuring gas exchange parameters and conducting quantitative RT-PCR (qRT-PCR) transcript profiling of genes well known for their roles in mediating drought responses: aquaporin (AQP) water channels and dehydration-responsive element binding (DREB) transcription factors. The defense response of these trees was assessed by measuring lesion length, changes to phloem cellular structure and qRT-PCR transcript profiling of secondary phloem for selected genes encoding TPSs and CHIs. We tested whether all defense-associated genes would respond in the same manner to water-deficit-induced stress, and if not, whether reduced carbon gain would impact the trees' carbon-based defenses more than nitrogen-based defenses. We also examined whether the effect of water deficit on tree defense gene expression was manifested both locally and systemically. The results of the study are discussed within the context of pine–MPB co-evolutionary relationships and ecological plant defense theory.

## Materials and methods

### Field study

The study was conducted at Chickadee, a naturally regenerated, thinned stand of ∼60-year-old *P. contorta* × *banksiana* hybrid pine trees (*Pcb*) located 37 km northwest of Whitecourt, Alberta, Canada (54°13′N, 116°03′W). The plot size area was 5513 m^2^. The hybrid status of the trees in the stand was confirmed by microsatellite analysis according to Cullingham et al. (2011). The experiment was conducted from May to August 2009, corresponding to Julian Days 147–226.

Meteorological data to describe environmental conditions at the study site were obtained from Environment Canada's Whitecourt weather station (http://www.climate.weatheroffice.gc.ca). Regional meteorological data from Environment Canada were used as the input to derive Climate Moisture Index (CMI) values according to Hogg (1997). The model was implemented within the BioSIM framework (Régnière 1996); 1971–2000 values were computed using 20 random replicates, and used to generate spatially interpolated maps.

### Water deficit and fungal inoculation treatments

A total of 40 trees with no evident history of disease or mechanical damage, of similar height and with a diameter at breast height between 16 and 24 cm were randomly selected. A two-by-two factorial design with two treatment factors, water level and inoculation with *G. clavigera*, was performed. The 40 experimental trees were randomly assigned to one of four treatments, with 10 trees per treatment: well-watered/uninoculated control, water-deficit/uninoculated control, well-watered/*G. clavigera* inoculated and water-deficit/*G. clavigera* inoculated. Well-watered and water-deficit trees were separated by a minimum of 11 m and a maximum of 126 m in the experimental design. Water treatments (well-watered or water deficit) were applied for 6 weeks before the fungal inoculation was applied. Water deficit was induced placing woven laminated polyethylene tarps (12′ × 14′; GH Factory Sales, Surrey, BC, Canada) around the base of each tree extending to the drip line from 27 May until termination of the experiment on 13 August. Well-watered trees were watered every 2 weeks from 12 June to 7 August using 40-gallon arborist's water bags (Treegator^®^, Rittenhouse, St. Catharines, ON, Canada) placed around the base of each tree. Soil relative water content (RWC) was measured using a time-domain reflectometer (TDR; Tektronix 1502B Cable TDR Cable Tester; Tektronix, Inc., Scarborough, ON, Canada). The relative water content (θ) was determined as a percentage calculated using an equation for organic soils calibrated for sandy soil (Topp et al. 1980, Robinson et al. 2003). Probe rods for measuring the RWC were placed 1-m away from the trunk, at three different depths (30, 60 and 90 cm) in the ground.

Trees were inoculated with *G. clavigera*—considered a moderately virulent fungal pathogen (Six and Wingfield 2011)—on 9 July 2009 according to Rice et al. (2007). The *G. clavigera* inoculum was derived from cultures initiated from an isolate cultured from a single MPB larva collected in 2007 from a naturally attacked tree near Fox Creek, Alberta, Canada (54°48′N, 116°63′W), close to the study site. It was reasoned that the haplotype represented by this culture would be the same or similar to *G. clavigera* carried by MPB attacking trees in the region. Molecular analyses confirmed the identity of the isolate as *G. clavigera* (Roe et al. 2010, 2011). The isolate corresponds to specimen M001-03-03-07-UC04DL09 described in Roe et al. (2010, 2011). A cordless drill was used to make 40 holes of ∼8 mm diameter per tree; holes were positioned 2–3 cm apart and arranged in two rings, one at breast height and the second 40 cm above breast height. Following inoculation, each hole was plugged with a small piece of sterilized dowel rod, and the ring of inoculations was wrapped using Parafilm^®^ strips (Pechiney PM992) to reduce environmental contamination. Mock-inoculated trees were not included in the experimental design due to the logistics of adding additional trees to a study conducted in a natural stand.

Approximately 5 weeks after inoculation, phloem was sampled by removing the bark above and below the inoculated area, and peeling the phloem layer away from the remaining outer tissue of the bark. Phloem adjacent to the maximum vertical extension of the lesion (outside lesion), and ∼1 cm inside of the lesion boundary (inside lesion), were collected separately for gene expression. Tissues collected for gene expression analysis were frozen in liquid nitrogen immediately after harvesting and stored at –80 °C until analysis. Eight to 10 trees were sampled for each treatment. Following harvest of material for molecular analyses, lesions were measured from the remaining bolts (wood) on 12 August 2009. Vertical extents of the lesion were delimited as the outermost discolored edge on the sapwood above and below the inoculation point.

### Gas exchange

Stomatal conductance (*g*_s_) and photosynthesis (*A*) were measured between 10:00 and 15:00 h using an open gas exchange system (Li-6400; Li-Cor, Inc, Lincoln, NE, USA). Measurements were performed on three fascicles of fully expanded current year needles. The plants were exposed to red/blue light (6400-02B LED Light Source) of ∼1200 μmol photons m^−2^ s^−1^ for 5 min prior to measurements. The cuvette CO_2_ concentration was set to 400 μmol CO_2_ mol^−1^. Leaf temperature in the chamber was controlled between 18 and 20 °C using a Peltier cooling system. Gas exchange was measured three times on each set of needles and the mean of the three measurements used for the analysis.

### Microscopy

Preparation of material for staining was carried out as described in El Kayal et al. (2011). Pieces of phloem ∼3 mm^2^ were placed directly in fixative (2% v/v glutaraldehyde, 1% caffeine buffered in 0.1 M sodium phosphate buffer; pH 7.2). Following successive ethanol dehydration and infiltration, samples were embedded in JB-4 Plus^®^ (Polysciences, Hatfield, PA, USA) at 55 °C overnight. Four-micrometer sections were stained with Richardson's stain (Richardson et al. 1960) or periodic acid-Schiff's (PAS) reagent for sugar residues with vicinal hydroxyl groups (Franceschi et al. 2000). In the latter procedure, sections were incubated for 30 min in 1% w/v periodic acid at room temperature, washed with distilled water and air-dried. Some sections were incubated in distilled water as non-oxidized control samples. Sections were then incubated in Schiff's reagent in the dark at room temperature for 1 h, rinsed with distilled water, counterstained with hematoxylin, and then dehydrated and mounted.

### Sequence analysis

Expressed sequence tags are publicly accessible at National Center for Biotechnology Information (NCBI) under accession numbers GW738148-GW774480 (*P. banksiana*) and GT229582-GT270713 (*P. contorta*). The corresponding transcriptome resources are described in Hall et al. (2013*b*). Contigs corresponding to putative *AQP*, *DREB*, *CHI* and *TPS* genes were identified by BLASTX using previously characterized sequences from other species as queries. Sequences used for subsequent analyses are provided in File S1 available as Supplementary Data at *Tree Physiology* Online. Open reading frames were predicted using Geneious 4.8.2 (Drummond et al. 2010). Multiple sequence alignments of deduced amino acid sequences and neighbor-joining phylogenetic analyses were performed in MEGA v4.0 (Tamura et al. 2007). Dendrograms were constructed using pairwise deletion and 1000 bootstrap replicates. The phylogenetic analyses were used to select genes for qRT-PCR based on position relative to functionally characterized genes from other species.

### Quantitative RT-PCR

Quantitative RT-PCR was carried out as described in El Kayal et al. (2011). Total RNA extractions (∼100 mg tissue per extraction) were carried out according to Pavy et al. (2008), quantified with a NanoDrop 1000 (Thermo Fisher Scientific, Waltham, MA, USA) and assessed with a 2100 Bioanalyzer (Agilent, Mississauga, ON, Canada). Two micrograms of total RNA was treated with DNaseI (Invitrogen; Life Technologies, Burlington, ON, Canada) prior to cDNA synthesis using Superscript II reverse transcriptase, following the manufacturer's protocols (Invitrogen).

Primer Express (v3.0, Applied Biosystems, Mississauga, ON, Canada) was used to design primers for qRT-PCR (see Table S1 available as Supplementary Data at *Tree Physiology* Online). Quantitative RT-PCR reactions (10 μl) consisting of a master mix (0.2 mM dNTPs, 0.3 U Platinum Taq Polymerase (Invitrogen), 0.25× SYBR Green, and 0.1× ROX), 20 ng diluted cDNA and 0.8 μM primers were analyzed as described in El Kayal et al. (2011). Three technical replicates were run for each biological replicate. Transcript abundance was quantified using standard curves generated from serial dilutions of *P. contorta* PCR products. *Eukaryotic translation initiation factor 5A-1* (*eIF-5A*; accession number KF322083) was used as the reference gene, since transcript abundance corresponding to this gene was not significantly different across treatments (*P* = 0.34).

### Statistical data analysis

Analysis of variance (ANOVA) was performed using the *Proc Mixed* procedure of SAS (9.1) (SAS Institute, Cary, NC, USA, 2002). One-way ANOVA was performed to test for variation in lesion length data. Two-way ANOVA was used to test for variation in gas exchange and gene expression under water level and pathogen treatment combinations. A Kenward–Roger option was used to calculate denominator degrees of freedom. Least squared means were separated using the pdiff option, and *P* ≤ 0.05 was used to determine significances between treatments. To meet the assumptions of normality and homogeneity of variance, log transformation was applied to the gene expression values for all but two genes.

Principal component analysis (PCA) was used to assess the relationship between genes with similar expression profiles. The PCA was performed using the Vegan package from R version 2.11.0 (R Development Core Team 2010; http://www.R-project.org). Significant contribution to the principal components was determined using the broken-stick distribution.

## Results

### Soil water status in the field experiment

**Figure 1.**
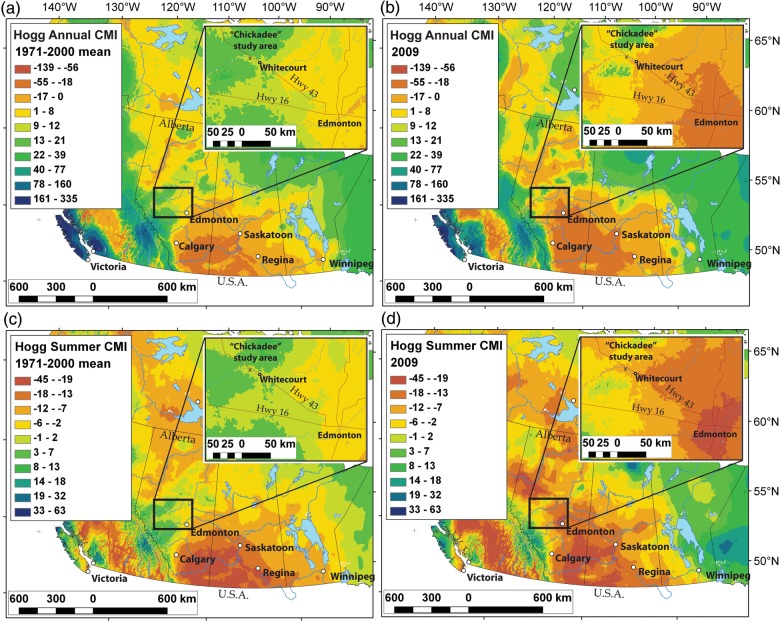
A comparison of normal (1971–2000) average CMI values for western Canada with those for 2009, the year during which the study was conducted. The inset maps depict the area of central Alberta in which the study site was located. The CMI was computed according to Hogg (1997), implemented within BioSIM (Régnière 1996) to generate spatially interpolated maps. (a) Annual average CMI for 1971–2000. (b) Annual CMI for 2009. (c) Average CMI for the months of May–August 1971–2000. (d) Average CMI for the months of May–August 2009, the period during which the study was conducted. Negative CMI values signify that mean precipitation is less than the estimated mean potential evapotranspiration, indicative of a predicted moisture deficit (Hogg 1997).

**Figure 2.**
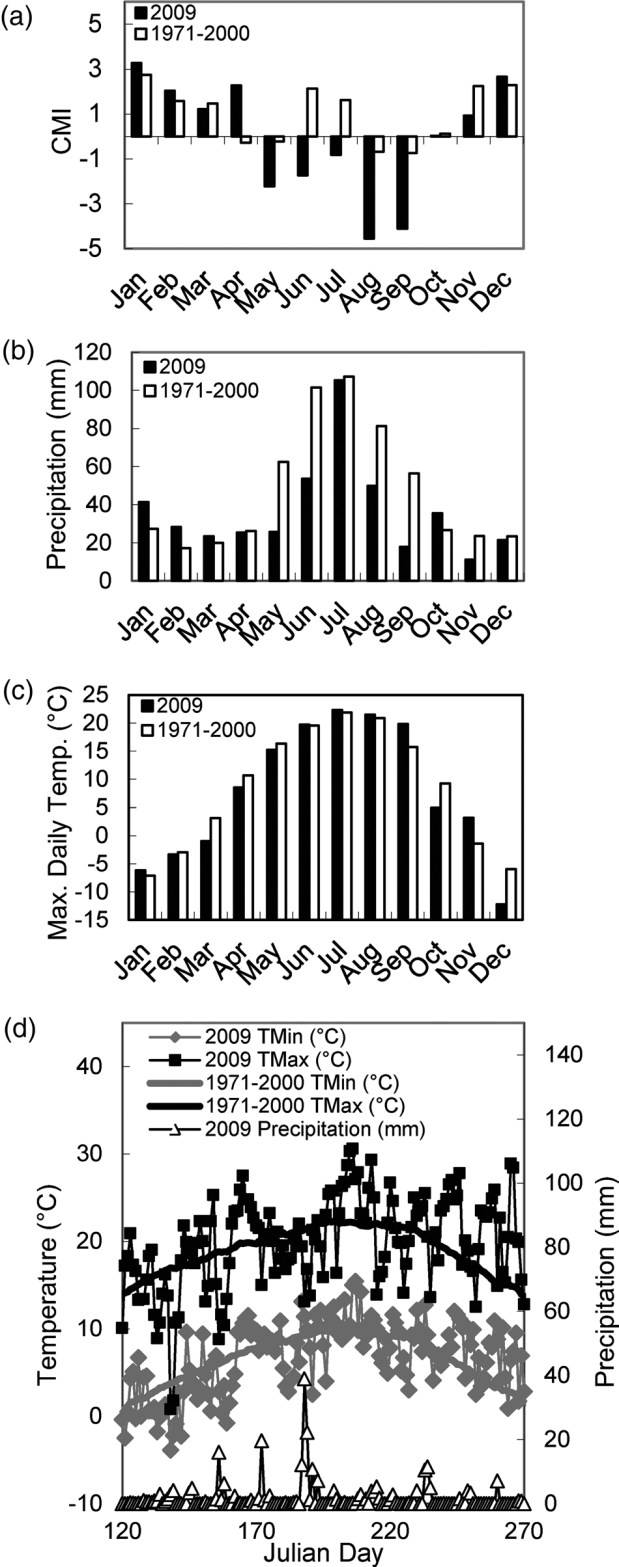
Comparison of 2009 to 1971–2000 average monthly and daily environmental data for the study site district. Data were obtained from Environment Canada's Whitecourt weather station, ∼37 km from the study site. (a) Climate Moisture Index calculated on a monthly basis for 2009 and the 1971–2000 average. Negative CMI values indicate a predicted moisture deficit. (b) Precipitation expressed as a monthly average, (c) maximum daily temperature expressed as a monthly average, (c) climate moisture index using the average of 1971–2000 and recorded data from 2009 in Whitecourt (AB). (d) Minimum and maximum temperature and precipitation averaged for 1971–2000 and the year of 2009. In a–c, black bars illustrate 2009 data, and white bars illustrate 1971–2000 data. In d, gray diamonds indicate daily temperature minima for 2009, black squares indicate daily temperature maxima for 2009, the gray thick line indicates average daily temperature minima for 1971–2000, the black thick line indicates average daily temperature maxima for 1971–2000, and white triangles indicate 2009 daily precipitation.

The period during which the field experiment was conducted—May to August 2009—was drier than normal in much of Alberta, particularly in the central region of the province where the study was conducted. BioSIM (Régnière 1996) was used to generate spatially interpolated maps of Hogg's CMI, which takes into account both precipitation and potential evapotranspiration (Hogg 1997). Figure 1 illustrates the reduced moisture availability in the region of the study site in 2009 relative to the 1971–2000 mean, particularly for the summer months (May to August) when the experiment took place. The CMI at the Chickadee study site was negative throughout the duration of the study—indicating a moisture deficit—and was lower than the CMI for the 1971–2000 mean (Figure 2a). Precipitation in the study area was lower than the 1971–2000 mean throughout much of the experiment except for July, when near-normal precipitation levels were due mainly to a single weather event (Figure 2). In contrast, monthly temperature averages for 2009 were very similar to those for the 1971–2000 mean, particularly during the experiment (Figure 2). Daily maximum temperatures were at or below normal for much of the duration of the experiment, notably from mid-June to mid-July (Julian Days 170–196) and during data and sample collection in August (Julian Days 222–226).

**Figure 3.**
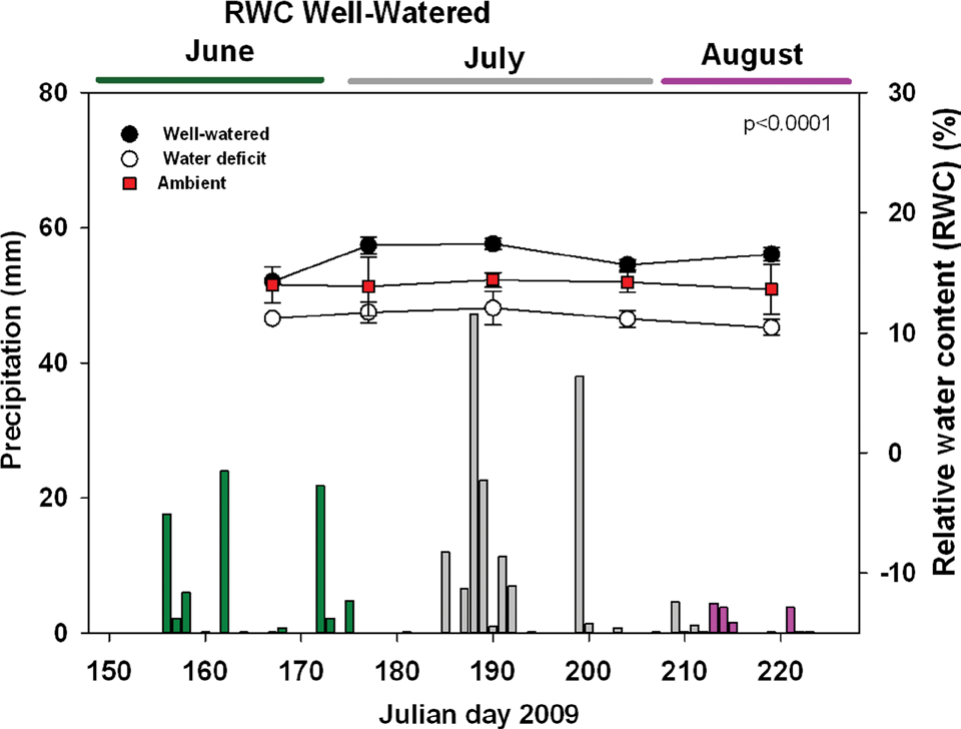
Soil RWC corresponding to well-watered and water-deficit trees over the course of the study. Mean values (±SE) for 20 trees across the field site, measured at several time points, are shown. Measurements were taken at 90 cm below the soil surface. RWC data obtained at 30 and 60 cm below the soil surface are not shown. *P* < 0.0001 indicates significance at the 5% level between RWC of well-watered and water-deficit treatments. Precipitation over the duration of the experiment, as measured at the Environment Canada Whitecourt station, is also shown.

Soil relative water content values at a depth of 90 cm averaged 17 ± 1.3% and 11 ± 0.6% for well-watered and water-deficit trees (Figure 3), respectively, demonstrating that water availability differed between well-watered and water-deficit treatments (*P* < 0.0001). These values bracketed those obtained for ambient soil RWC obtained at the study site. Relative water content values were relatively constant in well-watered trees at each of the three measured depths. Rain events did not have substantive effects on the RWC.

### Physiological and anatomical responses to plant water and fungal inoculation status

**Figure 4.**
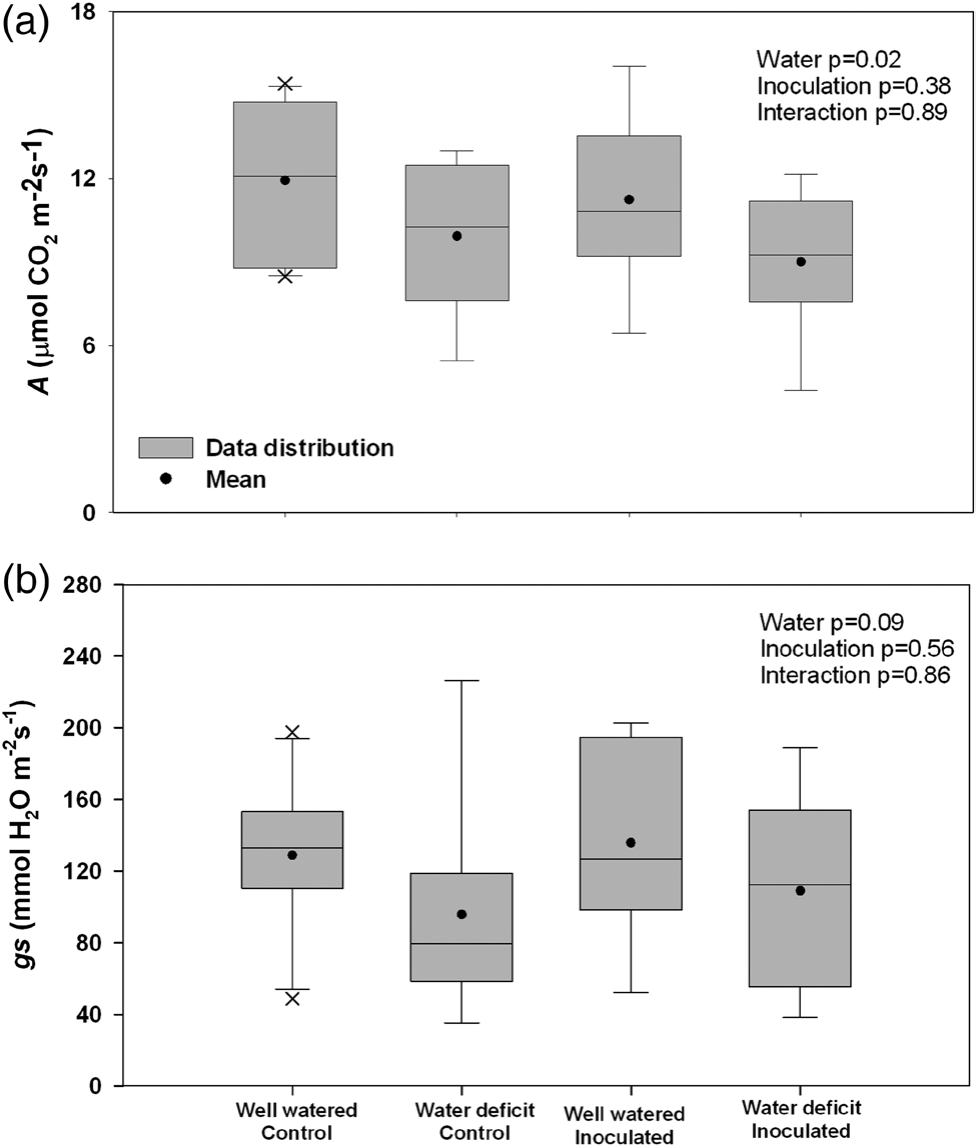
Water-deficit-induced changes to *A* and *g*_s_. (a) Photosynthesis (μmol CO_2_ m^−2^ s^−1^) and (b) *g*_s_ (mmol m^−2^ s^−1^) in mature *P. contorta* × *banksiana* trees subjected to well-watered or water-deficit conditions, either as unioculated controls or 5 weeks following *G. clavigera* inoculation. For each box plot, the upper and lower limits of the box indicate the 25th and 75th percentiles; the line through the box is the median; the capped lines indicate the 10th and 90th percentiles, and the black circle is the mean (*n* = 8–10). Analysis of variance results are indicated in the top right corner of each panel.

Stomatal conductance and *A* are key physiological indicators of water limitation stress for isohydric species such as pines (Klein et al. 2011). Mature *P. contorta* × *banksiana* hybrids trees subjected to water deficit exhibited significantly reduced *A* (*P* = 0.02) and nearly significantly reduced *g*_s_ (*P* = 0.09) after 11 weeks of treatment (Figure 4). Inoculation with *G. clavigera* did not significantly affect either *A* (*P* = 0.38) or *g*_s_ (*P* = 0.56). Similarly, the interaction terms for both *A* and *g*_s_ were not significant (*P* = 0.89 and *P* = 0.86, respectively).

**Figure 5.**
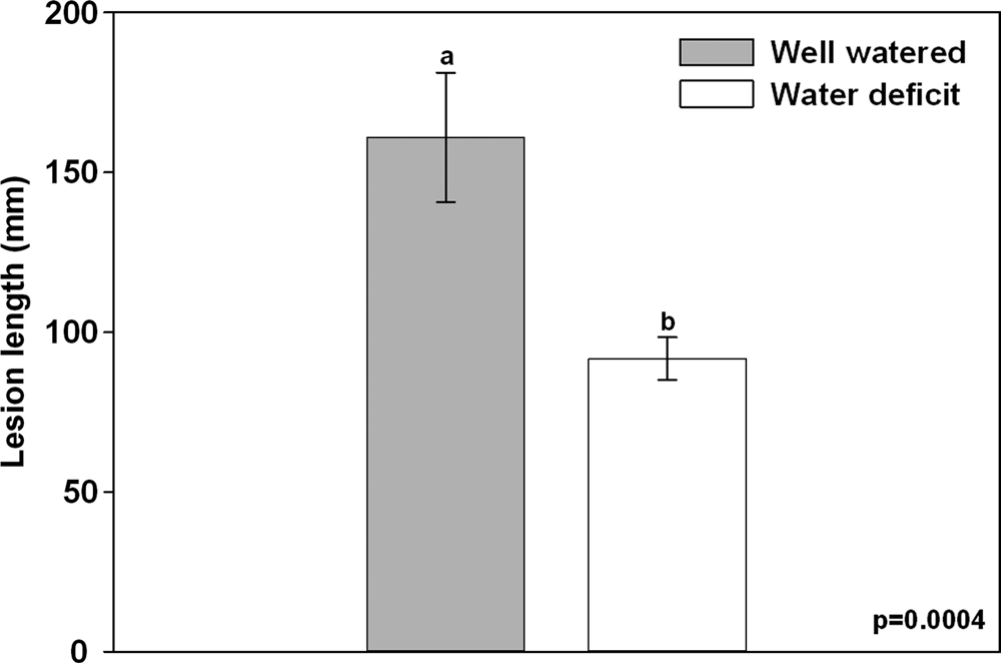
Lesion lengths of mature *P. contorta* × *banksiana* trees 5 weeks post-inoculation with *G. clavigera.* Different letters denote statistical differences at *P* = 0.05. Values are the mean ± SE for 10 trees. Ten lesions were measured per tree.

At 5 weeks post-inoculation, lesions surrounding each inoculation point were generally of a uniform elongated oval shape extending equally above and below the inoculation point. Lesion lengths were significantly greater in well-watered trees than in water-deficit trees (*P* = 0.0004; Figure 5).

**Figure 6.**
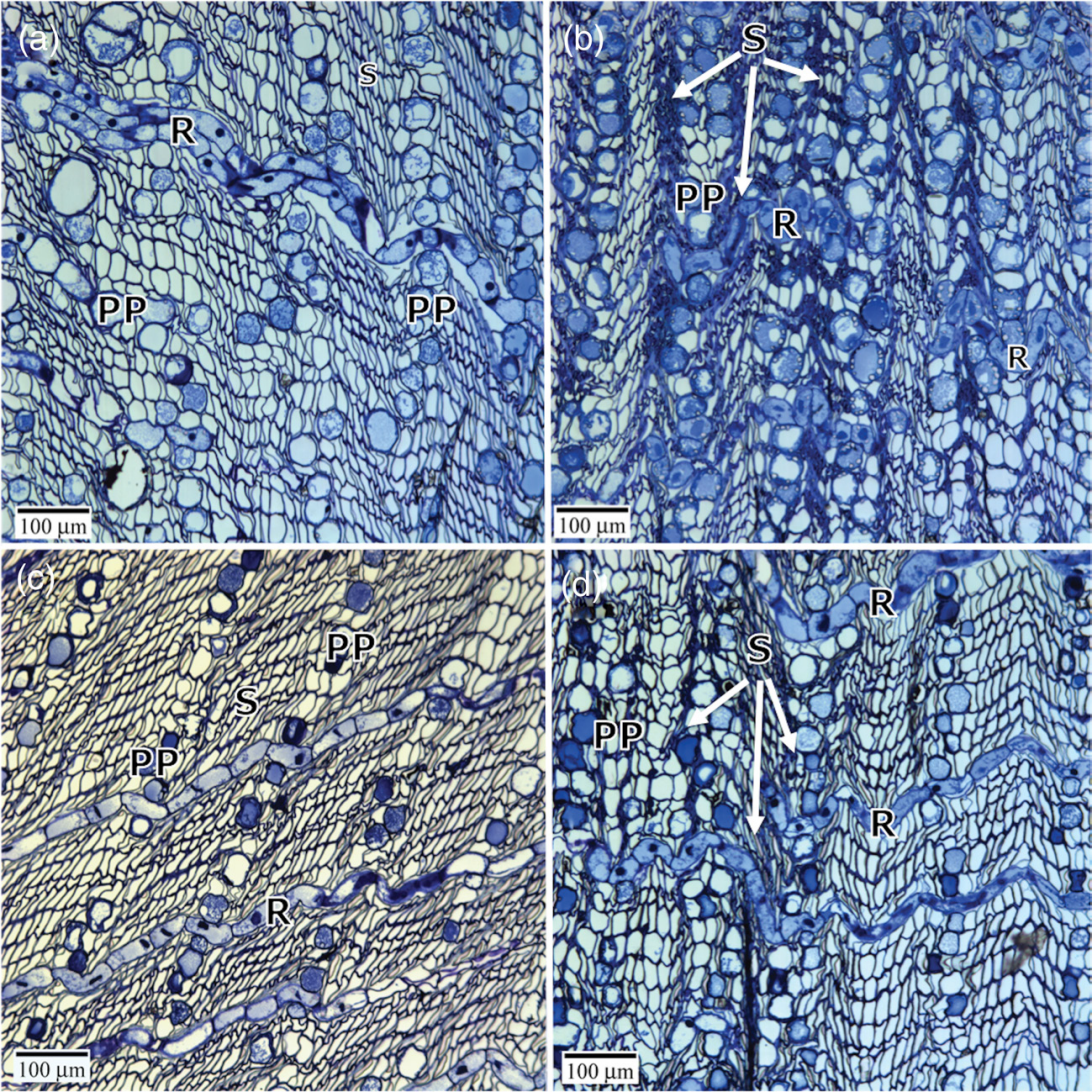
Effect of water deficit and *G. clavigera* on the secondary phloem cellular structure of *P. contorta* × *banksiana* mature trees. Transverse sections were stained with Richardson's stain. (a) Well-watered, uninoculated control tree. (b) Well-watered tree, 5 weeks following inoculation with *G. clavigera*. (c) Water deficit, uninoculated tree. (d) Water-deficit tree, 5 weeks following inoculation with *G. clavigera*. Polyphenolic parenchyma cells are more densely stained in well-watered inoculated trees relative to the other treatments. Arrows indicate collapsed sieve cell layers that are more evident in inoculated trees (b and d), but were also observed in some water-deficit uninoculated trees. PP, polyphenolic parenchyma cells; R, ray parenchyma; S, sieve cells. Scale bar = 100 μm.

Comparison of phloem anatomy between the four treatments revealed slight swelling of the polyphenolic parenchyma cells (PP cells) in inoculated trees, concomitant with compressed sieve cells (Figure 6). Polyphenolic parenchyma cells exhibited more densely staining phenolic bodies under water deficit compared with well-watered conditions. In addition, more abundant bodies, assumed to be starch grains due to preferential staining with PAS, were apparent around the PP cell periphery in inoculated well-watered trees. The PP cell phenolic bodies were mostly lightly stained with PAS, with a few of the PP cells in the outer layers having darker stained phenolic bodies (see Figure S1 available as Supplementary Data at *Tree Physiology* Online).

### Water-deficit- and fungal-induced changes in transcript abundance profiles of putative drought-associated genes

To determine whether the water stress imposed upon the trees was sensed at the molecular level and what effect *G. clavigera* inoculation had on this response, we generated transcript abundance profiles corresponding to several genes belonging to the *AQP* and *DREB* families. Members of the *AQP* and *DREB* families are classically associated with responses to water deficit and other abiotic stresses, and as such represent appropriate reporters for detecting plant responses to water deficit (Ozturk et al. 2002, Agarwal et al. 2006, Almeida-Rodriguez et al. 2010). Since no sequence resources were available for *P. contorta* × *banksiana*, we identified AQP- and DREB-like sequences for transcript abundance profiling by mining Sanger sequence data for pure *P. contorta* and *P. banksiana*. The *P. contorta* and *P. banksiana* sequences were characterized by phylogenetic analyses together with the corresponding *Arabidopsis thaliana* gene family and selected functionally characterized genes from other species, as well as by identification of conserved motifs typical of AQPs and DREBs (see Figures S2–S5 available as Supplementary Data at *Tree Physiology* Online). Because *P. contorta* × *banksiana* hybrids in this region typically demonstrate a greater proportion of *P. contorta* ancestry (Cullingham et al. 2012), we elected to choose *P. contorta* genes as the basis for designing qRT-PCR assays.

**Figure 7.**
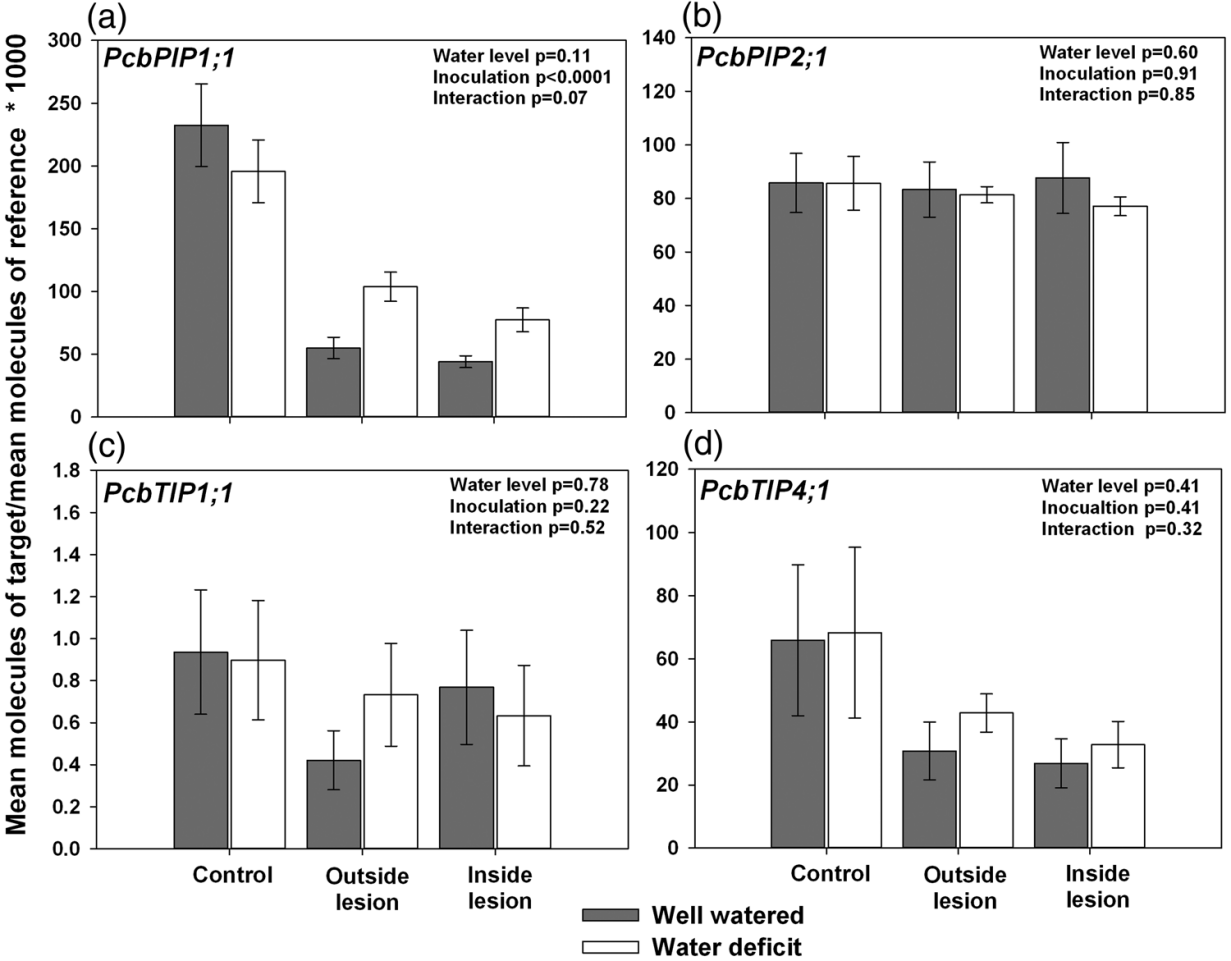
*AQP* transcript profiling in secondary phloem of mature *P. contorta* × *banksiana* trees subjected to water deficit and/or inoculation with *G. clavigera* for 35 days. Phloem from trees subjected to *G. clavigera* inoculation were sampled both outside and within the lesion zone for expression analyses. *eIF-5A* was used as the reference gene. Values represent the mean ± SE, *n* = 7–10. Analysis of variance results are indicated in the top right corner of each panel. For statistical analyses, data for most genes were log transformed to meet the assumptions of normality and homogeneity of variance.

Four pine *AQP* genes of the tonoplast intrinsic protein (*TIP*) and plasma membrane intrinsic protein (*PIP*) subfamilies were selected for transcript abundance profiling (see Figures S2 and S3 available as Supplementary Data at *Tree Physiology* Online). Members of these subfamilies have been shown to function as water channels and some are involved in water-deficit responses (Alexandersson et al. 2005, Zhang et al. 2007, Kaldenhoff et al. 2008). The *P. contorta* deduced amino acid sequences included typical MIP features such as a highly conserved asparagine–proline–alanine (NPA) motif and histidine residues involved in pH sensing (see Figure S2 available as Supplementary Data at *Tree Physiology* Online; Shelden et al. 2009, Park et al. 2010). The selected *P. contorta* genes were named *PcPIP1;1*, *PcPIP2;1*, *PcTIP1;1* and *PcTIP4;1* to reflect their placement in the phylogenetic tree and AQP naming conventions (see Figure S2 available as Supplementary Data at *Tree Physiology* Online). Transcript abundance profiles for the corresponding genes in *P. contorta* × *banksiana* hybrids indicated that water deficit had no significant effect on transcript abundance in control samples for any of the four *AQP*s that were examined. Similarly, there were no significant differences in transcript levels of *PIP2;1 TIP1;1* and *TIP4;1* (designated *PcbPIP2;1, PcbTIP1;1* and *PcbTIP4;1* for the hybrids) across water level and fungal inoculation treatments (Figure 7b–d). However, there was a significant decrease in transcript abundance of *PcbPIP1;1* in fungus-inoculated samples (*P* < 0.0001) both inside and outside of the lesion, although the interaction between water and inoculation treatments was nearly significant (*P* = 0.07; Figure 7a).

No pine sequences were identified in the Sanger dataset with similarity to the *A. thaliana DREB1* or *DREB2*, the best characterized of the dehydration-responsive *DREB* family members. Accordingly, five other members of the family were selected for transcript profiling, and named *PcTINY-like1*, *PcTINY-like2*, *PcDREB-like*, *PcRAP2.4-like* and *PcERF61-like* to reflect their relative placement on the tree. In silico analyses of these five *P. contorta* sequences indicated conservation of key amino acids necessary for DREB function (see Figure S5 available as Supplementary Data at *Tree Physiology* Online), including conserved tryptophan and arginine residues that interact with the GCC box cis-acting promoter element (Allen et al. 1998), an A38 residue important for stabilizing binding of the AP2 domain (Liu et al. 2006), either a serine or alanine at position 15 important for binding of DREB family transcription factors to ethylene-responsive elements (ERE) (Sun et al. 2008), and conservation of valine (V14) and glutamic acid (E19) residues, important for DREB binding to DRE elements (Sakuma et al. 2002).

**Figure 8.**
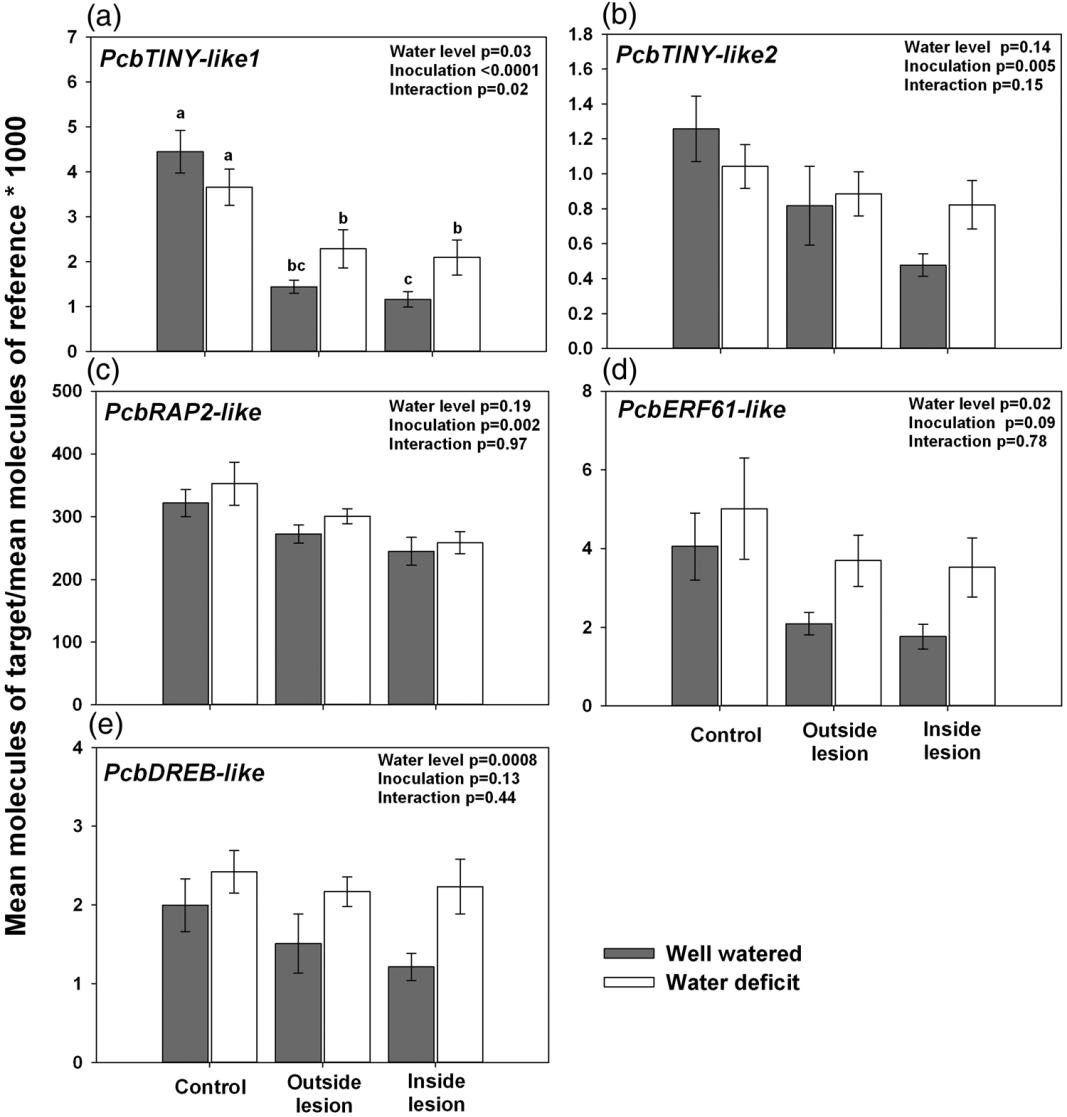
*DREB* transcript profiling in secondary phloem of mature *P. contorta* × *banksiana* trees subjected to water deficit and/or inoculation with *G. clavigera* for 35 days. Phloem from trees subjected to *G. clavigera* inoculation were sampled both outside and within the lesion zone for expression analyses. *eIF-5A* was used as the reference gene. Values represent the mean ± SE, *n* = 7–10. Analysis of variance results are indicated in the top right corner of each panel. For statistical analyses, data were log transformed to meet the assumptions of normality and homogeneity of variance. Different letters denote statistical difference at *P* < 0.05.

As with the *AQP*s, none of the *DREB* family members that were examined showed significant differences in transcript abundance between control well-watered and control water- deficit treatments in mature *P. contorta* × *banksiana* (Figure 8). However, both water deficit and fungal inoculation resulted in significantly decreased transcript abundance of *PcbTINY-like1* (*P* = 0.03 and *P* < 0.0001, respectively), with the interaction term also being significant (*P* = 0.02; Figure 8a). Fungal inoculation was associated with a significant decrease in transcript levels of *PcbTINY-like2* and *PcbRAP2-like* (*P* = 0.005 and *P* = 0.002, respectively), although water treatment did not have a significant effect on transcript abundance for these two genes, and the interaction term was not significant (Figure 8b and c). Conversely, water deficit resulted in significantly increased transcript abundance for *PcbERF61-like* and *PcbDREB-like*, compared with well-watered trees (*P* = 0.02 and *P* = 0.0008, respectively; Figure 8d and e). Decreases in transcript abundance on *PcbERF61-like* were observed due to fungal inoculation, although the effect was marginally significant (*P* = 0.09). A similar trend was observed in *PcbDREB-like* under well-watered conditions (Figure 8d and e).

### Water-deficit- and fungal-induced changes in transcript abundance profiles of defense-associated genes

Chitinases and TPSs are frequently associated with both constitutive and induced defense responses in conifers (e.g., Davis et al. 2002, Fossdal et al. 2005, Ralph et al. 2006, Zulak et al. 2009, Islam et al. 2010). Chitinases have been traditionally grouped into Classes I–VII based on biochemical and structural characterizations (Hamel et al. 1997, Brunner et al. 1998, Kasprzewska 2003), while more recent phylogenetic analyses group members into six clusters designated Clusters 1–6 (Xu et al. 2007). Phylogenetic analyses showed that the four *P. contorta CHIs* selected for transcript profiling were positioned in Clusters 1 and 3, which contain well-characterized pathogen-response *CHIs* belonging to Class IV and Classes I/II, respectively (see Figure S6 available as Supplementary Data at *Tree Physiology* Online). The *P. contorta CHIs* were named *PcCHI1.1, PcCHI2.1, PcCHI2.2* and *PcCHI4.1*, based on their placement in the phylogenetic tree. Placement within the tree enabled identification of *PcCHI4.1* as a Class IV CHI, while placement of the other three CHIs suggested that they could belong to either Class I or II. Multiple alignment of the deduced amino acid sequences was performed to determine the presence of different CHI domains used to distinguish different classes (see Figure S7 available as Supplementary Data at *Tree Physiology* Online). The presence of a chitin-binding domain, a complete catalytic domain and a carboxy-terminal extension showed that *PcCHI1.1* was a Class I CHI. *PcCHI2.1* and *PcCHI2.2* exhibited a deletion in the catalytic domain but no chitin-binding domain or carboxy-terminal extension, typical of Class II CHIs. *PcCHI4.1* exhibited a chitin-binding domain, no carboxy-terminal extension, and two deletions on the catalytic binding domain, providing further evidence that *PcCHI4.1* encodes a Class IV CHI (see Table S2 available as Supplementary Data at *Tree Physiology* Online).

**Figure 9.**
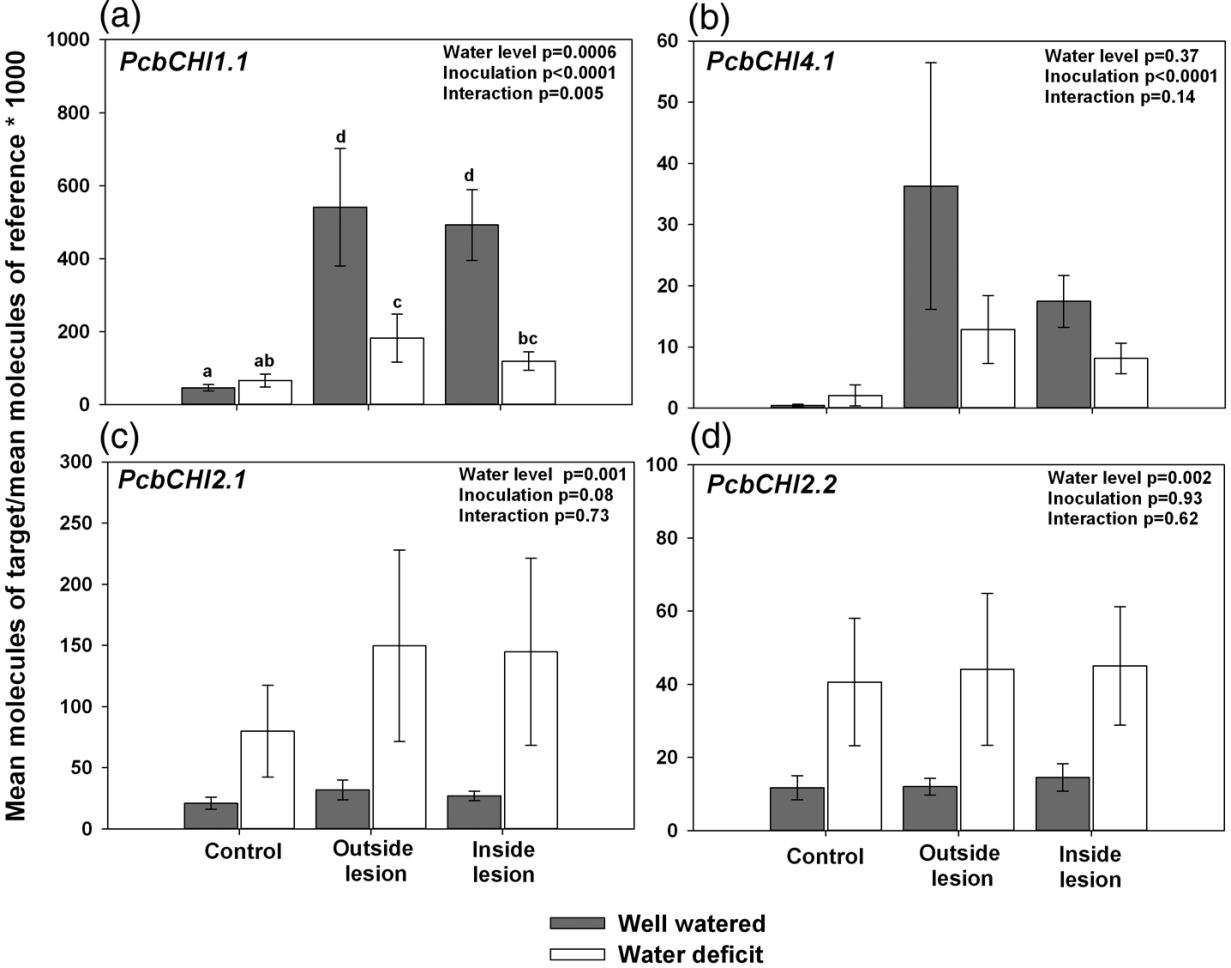
Chitinase transcript profiling in secondary phloem of mature *P. contorta* × *banksiana* trees subjected to water deficit and/or inoculation with *G. clavigera* for 35 days. Phloem from trees subjected to *G. clavigera* inoculation were sampled both outside and within the lesion zone for expression analyses. *eIF-5A* was used as the reference gene. Values represent the mean ± SE, *n* = 7–10. Analysis of variance results are indicated in the top right corner of each panel. For statistical analyses, data were log transformed to meet the assumptions of normality and homogeneity of variance. Different letters denote statistical difference at *P* < 0.05.

Quantitative RT-PCR transcript analysis revealed that most of the *CHIs* profiled responded to at least one of the two treatment factors (Figure 9). *PcbCHI1.1* transcript abundance significantly increased under both water availability and fungal inoculation treatments (*P* = 0.0006 and *P* < 0.0001); the interaction term was also significant (*P* = 0.005; Figure 9a). *PcbCHI4.1* showed significant increases in transcript levels due to fungal inoculation, but expression was unaffected by water deficit (*P* < 0.0001; Figure 9b). *PcbCHI2.1* and *PcbCHI2.2* both exhibited significantly increased transcript abundance under water deficit (*P* = 0.001 and *P* = 0.002, respectively; Figure 9c and d), but while *PcbCHI2.1* transcript abundance was marginally significantly greater in fungus-inoculated versus control samples (*P* = 0.08), fungal inoculation had no significant effect on *PcbCHI2.2* transcript abundance (Figure 9c and d). *PcbCHI5.1-like* did not show any significant changes in transcript abundance (Figure 9e).

Several lodgepole pine TPS genes have recently been cloned and the corresponding enzyme functions characterized (Hall et al. 2013*a*, 2013*b*). Four TPS genes were profiled: *PcTPS-(+)*α *pin1*, *PcTPS-3car1*, *PcTPS-LAS1* and *Pc(E)-*β*-farnesene synthase-like* (see Figures S8 and S9 available as Supplementary Data at *Tree Physiology* Online; Hall et al. 2013*a*, 2013*b*). Except for *Pc(E)-*β*-farnesene synthase-like*, substrate specificity consistent with their naming has been demonstrated for each of these TPSs (Hall et al. 2013*a*, 2013*b*).

**Figure 10.**
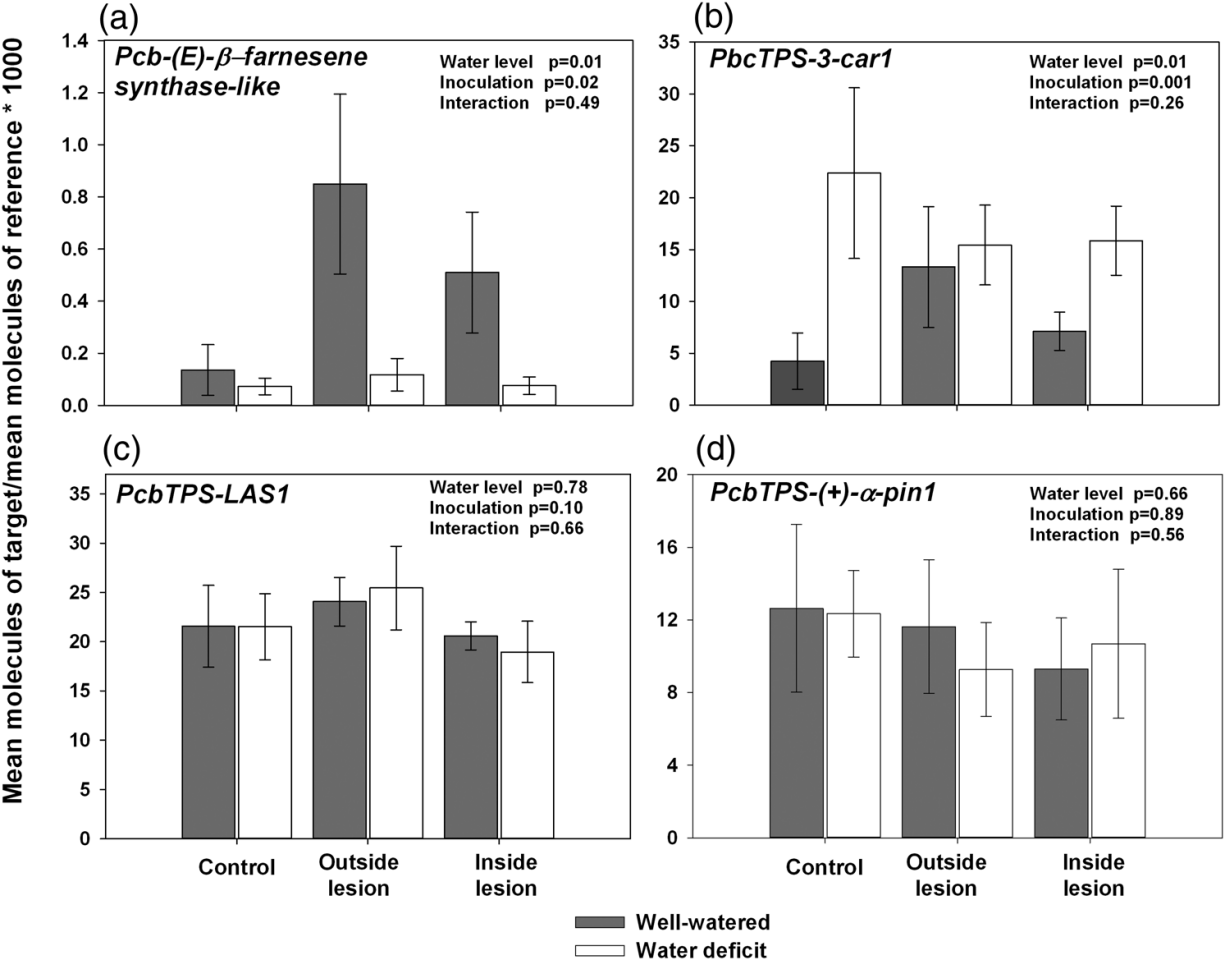
Terpene synthases transcript profiling in secondary phloem of mature *P. contorta* × *banksiana* trees subjected to water deficit and/or inoculation with *G. clavigera* for 35 days. Phloem from trees subjected to *G. clavigera* inoculation were sampled both outside and within the lesion zone for expression analyses. *eIF-5A* was used as the reference gene. Values represent the mean ± SE, *n* = 7–10. Analysis of variance results are indicated in the top right corner of each panel. For statistical analyses, data were log transformed to meet the assumptions of normality and homogeneity of variance.

The four TPSs displayed diverse transcript profiles in response to water deficit and fungal inoculation (Figure 10). *Pcb(E)-*β*-farnesene synthase-like* transcript levels showed significant differences under water availability (*P* = 0.01) and fungal inoculation (*P* = 0.02; Figure 10a). Although the interaction was not significant, increases in transcript abundance were observed under well-watered but not water-deficit conditions (Figure 10a). *PcbTPS-3car1* also showed significant differences in transcript abundance under water availability (*P* = 0.01) and fungal inoculation treatments (*P* = 0.0018), but the interaction term was not significant (Figure 10b). *PcbTPS-LAS1*and *PcbTPS-(+)*α *pin1* showed no significant differences between any of the treatments (Figure 10c and d).

### Multivariate analyses of transcript profiles

**Figure 11.**
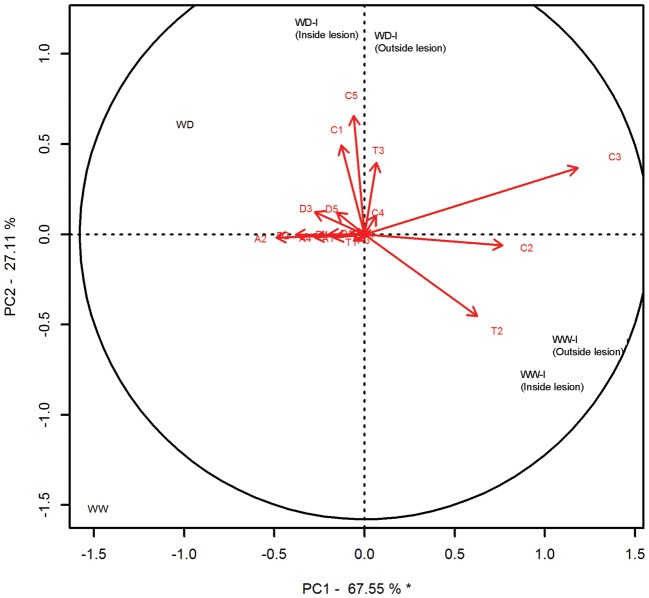
Principal component analysis to identify relationships between *AQP*, *DREB*, *TPS* and *CHI* phloem transcript abundance profiles, water availability and *G. clavigera* inoculation. The percentage of variation in the data explained by each principal component is provided on the graph axes, and principal components that are found to be significant based on the broken-stick distribution are noted with an asterisk. Gene expression profiles are shown as eigenvectors (arrows) and those that exceed the equilibrium circle contribute significantly to the principal components. WW, well-watered uninoculated trees; WD, water-deficit uninoculated trees; WW-I, well-watered fungus-inoculated trees; WD-I, water-deficit fungus-inoculated trees. Sampling of phloem inside or outside of the lesion is additionally indicated. A2, *PcbPIP1*;*1*; C1, *Pcbchi2.2*; C2, *Pcbchi4.1*; C3, *Pcbchi1.1*; C5, *Pcbchi2.1*; T2, *Pcb(E)- ± -farnesene synthase-like*; T3, *Pcb(+)-3-carene synthase*.

Principal component analysis was conducted on the transcript abundance profiles to identify the gene(s) explaining the greatest proportion of the variance between treatments (Figure 11). The first principal component, which was significant, accounted for 67.55% of the variability. Uninoculated treatments, water-deficit inoculated treatments and well-watered inoculated treatments separated into three distinct groups along PC1. The biplot showed that the inoculated/well-watered treatments were associated with increased transcript abundance of *Pcbchi1.1*, *Pcbchi4.1* and *Pcb(E)-*β*-farnesene synthase-like*, while the control uninoculated treatments were associated with increased transcript abundance for *PcbPIP1;1*, although none was statistically significant according to the broken-stick model.

The second principal component accounted for 27.11% of the variability. Water-deficit treatments were separated from well-watered treatments along PC2, which was driven mainly by increased expression of *PcbTPS-3car1*, *Pcbchi2.1-like* and *Pcbchi2.2-like* in water-deficit treatments and increased expression of *Pcb(E)-*β*-farnesene synthase-like* in well-watered treatments, although none was considered significant.

## Discussion

### Water deficit significantly reduced A in mature P. contorta × banksiana

Bark beetles such as MPB are able to overcome the critical threshold of resistance for physiologically stressed trees at lower attack densities than that of healthy and vigorously growing trees (Berryman 1982, Kolb et al. 1998, Wallin and Raffa 2002, Raffa et al. 2005). Our analyses establish that water- deficit conditions were experienced at the study site in 2009, the year in which the present study took place. The degree of water deficit to which the water-limited trees in the study site were exposed was augmented via water exclusion from the root area extending to the tree's drip line, while well-watered treatments were not subjected to this water deficit through active watering. Pine species exhibit a relatively conservative water-use strategy, and exert strong regulation over *g*_s_ for control of transpiration under water-deficit conditions (Andrews et al. 2012). As in previous studies with pines (e.g., Guérard et al. 2007, Domec et al. 2009, Lusebrink et al. 2011), the degree of water limitation imposed by the combination of environmental conditions and experimental manipulation was sufficient to cause decreased *g*_s_ and *A* in the water-deficit *P. contorta* × *banksiana* hybrids, although the former was not statistically significant at α = 0.05. Consequently, we can infer that carbon gain was reduced in water-deficit trees relative to well-watered trees.

There are at least three possible explanations for why greater differences in *g*_s_ were not observed. First, there is considerable tree to tree variation in both photosynthesis and *g*_s_ values, as illustrated by the box plot in Figure 4. This likely reflects genetic variation between biological replicates—the study was conducted in a naturally regenerated stand rather than a plantation—as well as micro-site variation. Second, temperatures were at or below normal during the week in which *A* and *g*_s_ were measured. We speculate that under these average temperatures, evapotranspiration may have been insufficient to promote greater stomatal closure. If the water-limiting conditions had been accompanied by high temperatures, as is often the case under field conditions, it is possible that *g*_s_ would have been further reduced in water-deficit trees. Third, pure jack pine seedlings from Saskatchewan show greater *g*_s_ under water-deficit conditions than lodgepole pine seedlings from western Alberta (Lusebrink et al. 2011). Jack pine is also more likely to be found on drier sites than lodgepole pine (Cullingham et al. 2012). Accordingly, it is possible that lodgepole × jack pine hybrids are more tolerant to water deficit than to lodgepole pine, although this remains to be tested.

### Water deficit alters P. contorta × banksiana defense responses to G. clavigera infection

Lesion length is a commonly used measure in studies of bark beetle–fungal associate–pine host interactions. In some studies, lesion length is considered an indicator of fungal pathogenicity and a measure of fungal success (e.g., Wong and Berryman 1977, Plattner et al. 2008, Rice and Langor 2008), with longer lesions generally interpreted to indicate greater fungal virulence (e.g., Lu et al. 2010). Lesion length is also considered an indicator of tree defense capacity (Paine and Stephen 1987), with longer lesions reported to be a reflection of increased release of toxic and/or inhibitory substances by trees in a defense response (e.g., Raffa and Berryman 1983, Christiansen et al. 1987, Raffa and Smalley 1995, Witzell and Martín 2010). In this study, inoculation with *G. clavigera* resulted in significantly larger lesions in well-watered trees compared with water-deficit trees after 5 weeks. Given that lesion length can be interpreted as an enhanced defense response and/or as a proxy for the extent of fungal invasion of the plant tissue, it is possible that reduced lesion length in water-deficit trees resulted from (i) induction of a defense response reduced either in total magnitude or delayed temporally and/or (ii) reduced fungal growth in the tree due to a less hospitable environment. Although not measured in this experiment, PCR detection of *G. clavigera* in inoculated lodgepole and jack pine seedlings suggest that the extent of *G. clavigera* growth is similar to the extent of lesion formation over time (A. Arango-Velez and J.E.K. Cooke, unpublished data), implying that these two possible explanations are not mutually exclusive. Further investigations of lesion development dynamics and the growth of fungi within the developing lesion are warranted to better understand how this measure reflects plant defense capacity.

Since measures of lesion length cannot be used to unambiguously assess the tree's defense response to inoculation with bark beetle fungal associates, particularly as a single time point measurement (Wallin and Raffa 2001), we turned to investigate the impact of water limitation and *G. clavigera* inoculation on transcript abundance profiles for *P contorta* × *banksiana* genes putatively involved in responses to biotic and abiotic stress. Several genes profiled by qRT-PCR showed significant changes in transcript profiles in response to inoculation with *G. clavigera*, suggesting a role for these genes in the response of *P. contorta* × *banksiana* to attack by this MPB fungal associate. Given that *P. contorta* × *banksiana* is beyond the historic range of MPB, it is considered a naïve host to MPB and by extension to *G. clavigera* (Cudmore et al. 2010, Safranyik et al. 2010). Consequently, it is likely that the molecular defense responses examined here represent more of a generalized response to phloem- and sapwood-invading fungal pathogens than an evolutionarily co-evolved adaptive or specialized response by the tree to *G. clavigera*. Historical exposure of trees in the *P. contorta*–*P. banksiana* complex zone to other Ophiostomoid fungi vectored by secondary bark beetles such as *Ips pini*, *Ips grandicollis* and *Dendroctonus valens* may also be reflected in the response patterns.

We observed that a subset of the defense-associated genes—*PcbCHI2.1*, *PcbCHI2.2* and *PcbTPS-3car1—*were up-regulated under water deficit in the absence of inoculation, while another subset of defense-associated genes—*PcbCHI1.1*, *PcbCHI4.1* and *Pcb(E)- ± -farnesene synthase-like*—showed attenuated expression upon challenge with *G. clavigera* under water deficit. These results are consistent with previously published reports in conifers that water limitation augments constitutive defenses but diminishes induced defenses (Lorio et al. 1995, Lombardero et al. 2000). They are also consistent with the findings of Lusebrink et al. (2011), who reported that water limitation resulted in a transient increase and subsequent decrease in total monoterpene levels, in both lodgepole and jack pine seedlings. Here, we use a finer-scale approach to demonstrate that both monoterpene-based and protein-based defenses are impacted by water limitation. The anatomical and cellular-level differences observed between *G. clavigera*-induced lesions of well-watered and water-deficit trees suggest that additional defense-associated metabolic pathways such as phenolic biosynthesis and cell wall biosynthesis and/or modification are also affected by water limitation.

Analysis of *PcTPS-3car1* demonstrated that this sequence encodes a functional 3-carene synthase (Hall et al. 2013*b*). 3-Carene synthases have a well-documented role in defense against herbivores in conifers (Fäldt et al. 2003, Robert et al. 2010, Hall et al. 2011). Interestingly, 3-carene makes up a greater proportion of the monoterpene profile of jack pine phloem than that of lodgepole pine (Lusebrink et al. 2011). Substrate specificity and product profiles for *Pcb-(E)-*β*-farnesene-like* have yet to be determined. Bohlmann et al. (1998) reported that a similar sesquiterpene synthase shows an inducible response upon wound treatments in *Abies grandis*. These TPS synthesize less of the oleoresin than of mono- or di-terpenes and are also involved in the transformation of terpenes into insect hormone mimics (Phillips and Croteau 1999).

*PcCHI1.1* encodes a Class I CHI, *PcbCHI4.1* encodes a Class IV CHI, and *PcbCHI2.1* and *PcbCHI2.2* exhibit the hallmarks of Class II CHI. The Class I CHI, *PcbCHI1.1*, appears to contain the carboxy-terminal extension required for vacuolar localization (Neuhaus et al. 1991), while the Class IV CHI, *PcbCHI4.1*, is most likely localized to the apoplast. In general, apoplastic CHI show activity in early stages of the pathogenic infection to generate elicitors that increase the signal and activate defense mechanisms, while vacuolar CHI are activated later and to greater levels, acting to degrade chitin of penetrating fungal hyphae (Kasprzewska 2003). Chitinases have often been reported in defense responses of conifers to pathogens. For example, Class II and Class IV CHI have been implicated in defense responses of Norway spruce to *Heterobasidion annosum* (Hietala et al. 2004) and of Douglas-fir to *Phellinus sulphurascens* (Islam et al. 2010), while Class I, Class II and Class IV CHI have been described in defense of slash pine to *Fusarium subglutinans* f.sp. *pini* (Davis et al. 2002). In the cases of Norway spruce–*H. annosum* and slash pine–*F. subglutinans* f.sp. *pini* interactions, there is an early, lower intensity induction of Class II and Class IV CHI in resistant tree genotypes, whereas there is a late, higher intensity induction of the CHI in susceptible tree genotypes. In these studies, the authors concluded that the early response exhibited by resistant genotypes constituted part of an effective containment strategy, while the high magnitude late response of the susceptible genotypes likely represented a delayed and ultimately unsuccessful attempt to contain the invading pathogen (Davis et al. 2002, Hietala et al. 2004). Similarly, Fossdal et al. (2007) demonstrated that drought resulted in an earlier induction of some defense-related genes in Norway spruce seedlings inoculated with *Rhizoctonia* sp. These studies reinforce the importance of the timing of the defense response. Due to the scale of this field study with mature trees in a natural stand, we were not able to include a temporal component; however, future studies will incorporate a time course to address the rate and magnitude of induction for CHI and other defense-associated genes.

Although originally selected as a candidate to assess abiotic (water limitation) stress, *PcbTINY1-like* was down-regulated in response to *G. clavigera* challenge, with greater decreases in transcript abundance exhibited by well-watered versus water- deficit trees. *PcbTINY1-like* exhibits greater similarity to the *ERF* subfamily of *DREB*s than to the *DREB* subfamily. While *DREB*s are usually induced by abiotic stresses and bind to DRE elements, *ERF*s are induced by biotic stresses and bind to ERE elements (Ohme-Takagi et al. 2000, Agarwal et al. 2006). AtTINY from *A. thaliana* binds to both DRE and ERE elements and is induced by abiotic stresses (drought and cold), and defense signaling molecules (ethylene and methyl jasmonate; Sun et al. 2008). Although *PcbTINY1-like* shows down-regulation rather than up-regulation in response to *G. clavigera*, the interaction observed between water deficit and *G. clavigera* inoculation hints that *PcbTINY1-like* could also play a role in mediating crosstalk between abiotic and biotic stress signaling.

Interestingly, most of the *G. clavigera*-responsive genes showed similar transcript abundance in phloem tissues within lesions and phloem tissues distinct from but surrounding the lesions, suggestive of a systemic response. This is consistent with previous cellular-level analyses demonstrating that production of both phenolic- and terpenoid-based compounds occurs some distance away from the site of attack, and can be transported to the defense zone (Franceschi et al. 2000, 2005, Hudgins et al. 2003). Induction of defenses in the tissues immediately surrounding the lesion may act to further constrain fungal growth. In the context of an MPB attack, this corresponds to the beetle's entry point into the host tree. If the tree is able to rapidly perceive the presence of *G. clavigera* upon attack by the first arriving MPB, these data suggest that the tree could mount a systemic induction of defense responses along the length of the trunk in time to defend against *G. clavigera* vectored by MPB that join the mass attack. Such systemic acquired resistance has been demonstrated in Norway spruce inoculated with *Ceratocystis polonica*, a fungal associate of the bark beetle *Ips typographus* (Christiansen et al. 1999). If this is true, we would predict that pine populations sharing a co-evolutionary history with MPB would be likely to perceive MPB-vectored *G. clavigera* more rapidly and more specifically than evolutionarily naïve pine populations, and be able to mount a systemic response in time to render greater protection against a mass attack. Over longer time frames, if the tree were successfully able to defend against an MPB mass attack, it is possible that the tree may acquire systemic induced resistance, a greater level of basal resistance providing greater protection against attacks in subsequent years (Bonello et al. 2006). Systemic induced resistance, also referred to as priming, has been demonstrated in Monterey pine inoculated with the fungus causing pitch canker, *Fusarium circinatum* (Bonello et al. 1991), and in Norway spruce inoculated with the root and butt rot pathogen *Heterobasidion parviporum* (Fossdal et al. 2012).

### How does water deficit alter P. contorta × banksiana defense response profiles?

Given that transcript abundance for genes belonging to both the *TPS* and *CHI* families of defense-associated genes are up-regulated under water-limiting conditions in the absence of *G. clavigera* inoculation, but that different genes belonging to the *TPS* and *CHI* families show attenuated expression under water-limiting conditions following *G. clavigera* inoculation, we infer that both carbon- and nitrogen-based defenses are impacted by water deficit. If the major effect of water limitation reduced photoassimilate availability for allocation to carbon-based defenses (Hodges and Lorio 1975, Lorio 1986, Christiansen et al. 1987, Byers 1995, Croisé et al. 2001), then we might have expected to observe a greater decrease in carbon-based defense gene expression than in nitrogen-based defense gene expression. This is not the case in this study. This finding is inconsistent with one of the predictions of the carbon-nutrient balance hypothesis (Bryant et al. 1983), which states that a change in resource conditions that decreases the carbon: nutrient ratio will lead to a decrease in non-nitrogen- (i.e., carbon-) based defenses, and a concomitant increase in nitrogen-based defenses. However, the finding is consistent with those of Guérard et al. (2007), who used radioisotope studies to demonstrate that carbon resources used in local defenses are derived mainly from locally stored carbon reserves rather than newly assimilated carbon.

Another effect of water limitation is acceleration of the seasonal transition from active growth to growth cessation, i.e., dormancy (Lorio 1986, Gruber et al. 2010). Although we did not explicitly examine growth cessation in this study, there are lines of evidence to suggest that water limitation promoted an early transition to growth cessation, particularly in *G. clavigera*-inoculated water-deficit trees. First, we observed that the bark was consistently much more difficult to remove from water- deficit trees than well-watered trees, particularly if trees had been inoculated, suggesting that the number of cell layers making up the cambial zone and recent derivatives was reduced. This could not be examined directly in this study because the cambial zone was destroyed during sampling. However, the compressed appearance of sieve cells in phloem of water-deficit trees—exacerbated in inoculated trees—hints that these trees were progressing toward growth cessation more quickly. Second, *PcbPIP1;1* was significantly down-regulated in *G. clavigera*-inoculated trees, and somewhat down-regulated in water-deficit control trees. A subset of PIPs mediate cell expansion in plant tissues (Maurel et al. 2002), suggesting that expression of *PcbPIP1;1* decreased as cell expansion of developing cells was completed and cells proceeded with maturation. Third, the Class II CHIs *PcbCHI2.1* and *PcbCHI2.2* exhibited up-regulation under water-limited conditions regardless of inoculation treatment, while *G. clavigera* inoculation had little effect on transcript abundance. Conifers synthesize a diverse array of abiotic and biotic defense-associated proteins, including CHIs, during autumnal acclimation, concomitant with the transition to growth cessation and well in advance of autumn-like environmental conditions (Holliday et al. 2008, Jarzabek et al. 2009, Cooke et al. 2012, Galindo González et al. 2012). Seasonally accumulated CHIs, including those from conifers, have been shown to exhibit chitinolytic activity, antifreeze activity or both (e.g., Zamani et al. 2003, Griffith and Yaish 2004, Stressmann et al. 2004, Jarzabek et al. 2009).

It is tempting to speculate that the effects of water deficit on patterns of constitutive defenses (Lombardero et al. 2000, Lusebrink et al. 2011) are due at least in part to contrasting phenologies between well-watered and water-deficit plants. Accelerated phenology of water-deficit plants may augment constitutive defenses as described above, providing some degree of generalized protection against pests and pathogens, while curtailing the capacity to mount inducible defenses. While seasonal effects on key conifer defenses such as oleoresin terpenoids have received relatively little attention, seasonal variation in oleoresin terpenoid composition has been documented in *Pinus halepensis* and *Pinus pinea* (Mita et al. 2002). The diminished capacity to mount an induced defense under water-limited conditions may in part be due to reduced photoassimilate availability that is a function of reduced photosynthesis as described above. In trees that are proceeding more quickly to growth cessation, we would also predict that carbon resources would be preferentially allocated in the stem to resource-intensive processes that accompany growth cessation such as cell wall biosynthesis and modification. We would further predict that carbon resources would be preferentially partitioned to perennating parts—particularly roots—for storage during the transition to growth cessation (Scarascia-Mugnozza et al. 1999), further reducing the availability of carbon skeletons for synthesis of defense-associated compounds.

The notion that water deficit accelerates the transition from active growth to growth cessation, thereby influencing the array of constitutive and inducible defenses manifested by the tree, is consistent with the growth–differentiation hypothesis (Lorio 1986, Herms and Mattson 1992). The central tenet of the growth–differentiation hypothesis is that an environmental factor that reduces growth (i.e., cell division and cell expansion) more than photosynthesis will result in a shift in allocation priority, so that a greater proportion of carbon resources are allocated to differentiation-related processes. Differentiation-related processes comprise a myriad of cellular, biochemical and molecular changes that occur as cells reach maturation, including physical and chemical defenses (Lorio 1986). Lorio (1986) invoked the growth–differentiation hypothesis to explain how seasonally correlated developmental events and defense processes impacted loblolly pine–southern pine beetle interactions, and how water deficit could impact these interactions via promoting a shift from early to late season development in the stem, and the reductions in resin production that accompany this developmental transition. Here, we extend Lorio's model to the developmental transition from latewood production to cessation of cambial growth, and the numerous changes that occur to defense capacity as a result of this transition. While the evidence presented in this study is circumstantial for the effect of drought being primarily one of accelerating phenology to promote early growth cessation, with concomitant effects on constitutive and inducible defense, we propose this framework as a testable model to form the foundation for future experimentation.

## Conclusions

Here, we identified a suite of genes from *P. contorta* × *banksiana* that respond to water deficit, *G. clavigera* inoculation or to both stressors in mature trees. The identification of *G. clavigera*-responsive genes provides us with the means to more accurately assess the defense responses of *P. contorta* × *banksiana* trees to an MPB attack than the traditionally used measure of lesion length. The expression profiles for this suite of genes demonstrated that, under water deficit, constitutive expression of some defense-associated genes was enhanced under water deficit, while the induction of other defense-associated genes upon *G. clavigera* inoculation was attenuated. That some genes respond to both stressors is suggestive of molecular crosstalk between water stress and defense responses in pines. The data also indicate that not all components of the constitutive and induced defenses are affected equally, suggesting that the effect of water deficit on the tree's complex defense machinery is not an ‘all-or-none’ response, but rather is quantitative in nature. The results suggest that if MPB and its fungal associates are able to overcome the constitutive defenses of a water-stressed tree, they are more likely to overcome the induced defenses.

In recent years, MPB has undergone considerable range expansion across northern Alberta (Cullingham et al. 2011). The pines of this landscape are considered naïve hosts, and as such may not have acquired specialized defenses against MPB and their fungal associates. Consequently, the threshold density for a successful MPB attack may be lower for hosts in these novel habitats. Our results suggest that water deficit could further lower the critical threshold at which MPB successfully attacks pines in the region by reducing key aspects of the induced defense response. Given the recent periods of drought experienced in the region (Hanesiak et al. 2011), this environmental factor could serve as a positive feedback on MPB dynamics and consequently continued range expansion (Raffa et al. 2008). Furthermore, the region is predicted to receive less precipitation in the future under scenarios of climate change (Schindler and Donahaue 2006). If the gene expression pattern and lesion development differences reported here translate into differences in defense capability of novel hosts against an attack by MPB and their fungal associates, further investigation into how water deficit affects pine defense capacity against MPB and its pathogenic fungal associates will be important to ascertain stand susceptibility in the face of this unprecedented MPB epidemic that continues to expand into novel territory in concert with changing climate conditions.

## Supplementary data

Supplementary data for this article are available at *Tree Physiology* Online.

## Conflict of interest

None declared.

## Funding

Funding for this research has been provided through grants from Genome Canada, The Government of Alberta through Genome Alberta, and The Government of British Columbia through Genome BC (The TRIA Project, http://www.thetriaproject.ca) to J.E.K.C. and B.J.C.; Alberta Innovates Bio Solutions to J.E.K.C. and B.J.C.; and The Canadian Foundation for Innovation to J.E.K.C.

## Supplementary Material

Supplementary Data
