# Supplementary material for: Influence of water deficit on the molecular responses of Pinus contorta × Pinus banksiana mature trees to infection by the mountain pine beetle fungal associate, Grosmannia clavigera
Source: Tree Physiol. 2013 Dec 5;34(11):1220–39. doi: 10.1093/treephys/tpt101 (PMC4277265; doi:10.1093/treephys/tpt101)
Supplement: Supplementary Data [file supp_34_11_1220__index.html]

Influence of water deficit on the molecular responses of Pinus contorta × Pinus banksiana mature trees to infection by the mountain pine beetle fungal associate, Grosmannia clavigera — Influence of water deficit on the molecular responses of Pinus contorta × Pinus banksiana mature trees to infection by the mountain pine beetle fungal associate, Grosmannia clavigera — Supplementary Data 

# Influence of water deficit on the molecular responses of *Pinus contorta* × *Pinus banksiana* mature trees to infection by the mountain pine beetle fungal associate, *Grosmannia clavigera*

## Supplementary Data

Supplementary Data

**Files in this Data Supplement:**

- Supplementary Data - Doc file
- Supplementary Figure 1 - tif file
- Supplementary Figure 2 - pptx file
- Supplementary Figure 3 - docx file
- Supplementary Figure 4 - pptx file
- Supplementary Figure 5 - docx file
- Supplementary Figure 6 - pptx file
- Supplementary Figure 7 - docx file
- Supplementary Figure 8 - pptx file
- Supplementary Figure 9 - docx file
- Supplementary File1 - docx file
- Supplementary Table 1 - docx file
- Supplementary Table 2 - docx file
