# Supplementary material for: Influence of water deficit on the molecular responses of Pinus contorta × Pinus banksiana mature trees to infection by the mountain pine beetle fungal associate, Grosmannia clavigera
Source: Tree Physiol. 2013 Dec 5;34(11):1220–39. doi: 10.1093/treephys/tpt101 (PMC4277265; doi:10.1093/treephys/tpt101)
Supplement: Supplementary Data [file supp_tpt101_tpt101supp.doc]

**Supplemental Files**

**Supplemental File S1.** Sequences used for phylogenetic analyses.

**Supplemental Table S1.** Gene name, EST pine database and sequences of primers used for qRT-PCR.

**Supplemental Table S2**. Characteristics of the major chitinase classes (Glycosyl hydrolase family 19) according to the domain structure as shown by Hamel et al. (1997).

**Supplemental Fig. S1**: Secondary phloem radial section of *P. contorta* x *banksiana* mature trees after five weeks of inoculation with *G. clavigera.*

**Supplemental Fig. S2**. Phylogenetic analysis of AQPs.

**Supplemental Fig. S3.** Multiple alignment of AQP deduced amino acid sequences*.*

**Supplemental Fig. S4.** Phylogenetic analysis of DREBs.

**Supplemental Fig. S5.** Multiple alignment of DREB deduced amino acid sequences.

**Supplemental Fig. S6**. Phylogenetic analysis of chitinases.

**Supplemental Fig. S7.** Multiple alignment of chitinase deduced amino acid sequences.

**Supplemental Fig. S8.** Phylogenetic analysis of TPS.

**Supplemental Fig. S9.** Multiple alignment of TPS deduced amino acid sequences.
