# Supplementary material for: Influence of water deficit on the molecular responses of Pinus contorta × Pinus banksiana mature trees to infection by the mountain pine beetle fungal associate, Grosmannia clavigera
Source: Tree Physiol. 2013 Dec 5;34(11):1220–39. doi: 10.1093/treephys/tpt101 (PMC4277265; doi:10.1093/treephys/tpt101)
Supplement: Supplementary Data [file supp_tpt101_tpt101supp_fig2.pptx]

## Slide 1
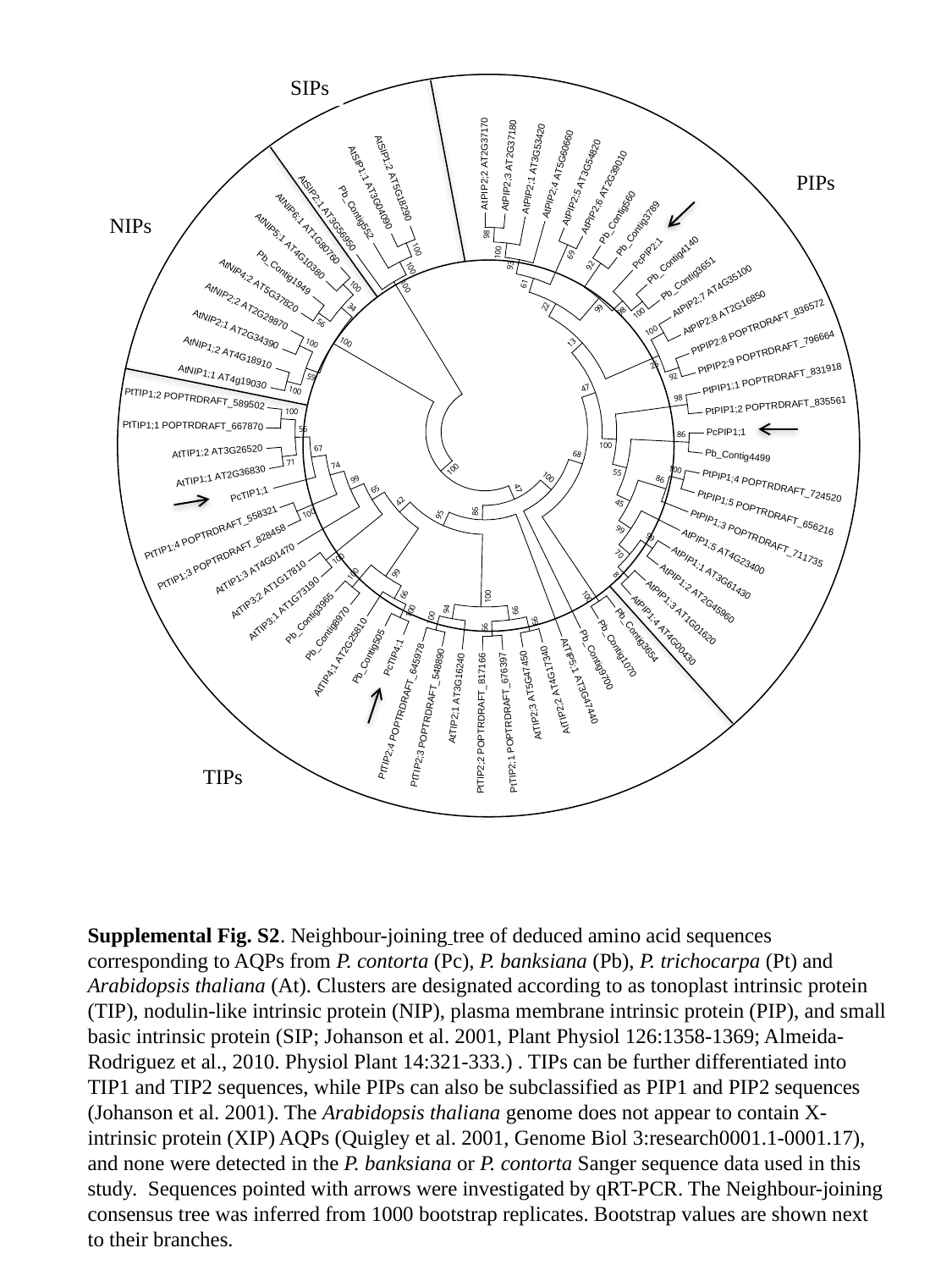

SIPs
PIPs
NIPs
TIPs
Supplemental Fig. S2. Neighbour-joining tree of deduced amino acid sequences corresponding to AQPs from P. contorta (Pc), P. banksiana (Pb), P. trichocarpa (Pt) and Arabidopsis thaliana (At). Clusters are designated according to as tonoplast intrinsic protein (TIP), nodulin-like intrinsic protein (NIP), plasma membrane intrinsic protein (PIP), and small basic intrinsic protein (SIP; Johanson et al. 2001, Plant Physiol 126:1358-1369; Almeida-Rodriguez et al., 2010. Physiol Plant 14:321-333.) . TIPs can be further differentiated into TIP1 and TIP2 sequences, while PIPs can also be subclassified as PIP1 and PIP2 sequences (Johanson et al. 2001). The Arabidopsis thaliana genome does not appear to contain X-intrinsic protein (XIP) AQPs (Quigley et al. 2001, Genome Biol 3:research0001.1-0001.17), and none were detected in the P. banksiana or P. contorta Sanger sequence data used in this study. Sequences pointed with arrows were investigated by qRT-PCR. The Neighbour-joining consensus tree was inferred from 1000 bootstrap replicates. Bootstrap values are shown next to their branches.
