# Supplementary material for: Influence of water deficit on the molecular responses of Pinus contorta × Pinus banksiana mature trees to infection by the mountain pine beetle fungal associate, Grosmannia clavigera
Source: Tree Physiol. 2013 Dec 5;34(11):1220–39. doi: 10.1093/treephys/tpt101 (PMC4277265; doi:10.1093/treephys/tpt101)
Supplement: Supplementary Data [file supp_tpt101_tpt101supp_fig3.docx]

1 90

PcPIP1;1 (1) ----MEEVGVGTSQYSERQPLGTSAQTDRESKDYYEPEPAPLFEPEELRSWSFWRAGIAEFMATFMLVYIT-LLTIMGVK-RSPTMCDSV

PtPIP1;1 POPTRDRAFT_831918 (1) MEEGEEDVKVGANRYGEGQPIGTAAQTQHG-KDYTEPPPAPLYQPGEWLSWSFYRAGIAEFVATFLFLYIT-VLTVMGVA-RSSTKCSTV

PcPIP2;1 (1) ------------------------------------------------------------------------------------------

PtPIP2;8 POPTRDRAFT_836572 (1) --------------------------MSTGGKDYRDPPPAPLLDMEELKQWSFYRALIAEFVATFLFLYIG-VGTVVGYKGVHNNLCDGA

PcTIP1;1 (1) ----------------------------------MPFGGIAVGRPEEATHPEALKAALAEGISTLIFVFAGEGSGMAFDKLTSDASTTPA

PtTIP1;2 POPTRDRAFT_589502 (1) ----------------------------------MPIRNIAVGHYHEATQPDALRAALAEFISTLIFVFAGEGSGMAFAKLTDGAANTPA

PtTIP2;1 POPTRDRAFT_676397 (1) ------------------------------------MVKIAFGSLGDSFSVGSLKAYLSEFIATLLFVFAGVGSAIAYSKLTTDAALDPP

PcTIP4;1 (1) ------------------------------------MAKIALGNRDEPARPDCVRAVFVELICTFLFVFAGVGSAMAMDQMSVPAN-SPA

91 180

PcPIP1;1 (85) GIQGIAWSFGAAVFALVYCTAGISGGHINPAITFGLFLARKLSLTRTVFYMICQCLGAIFGAGVVKGMQKGMYEVEGGGANIVAHGYTKG

PtPIP1;1 POPTRDRAFT_831918 (88) GIQGIAWAFGGMIFVLVYCTAGISGGHINPAVTFGLLLARKLTLTRAVFYMIMQCLGAICGAGVVKGFQKSPYEILGGGANTVSTGYSKG

PcPIP2;1 (1) -----------MIFVLVYCTAGS-GGHINPAVTFGLFLARKVSLPRAVLYMIAQCLGAICGTGLVKAFQKSFYDKYGGGANYVHNGYTKG

PtPIP2;8 POPTRDRAFT_836572 (64) GYLGVAWAFGGMIFVLVYCTAGISGGHINPAVTFGLFVARKVSLIRAVAYMMAQCLGAMLGVWMVMILTGIHYDQAGGAVNVVAPGYSKG

PcTIP1;1 (57) GLVAVALAHALGLFVAVAVGANISGGHVNPAVTFGAFVGGHITLLRGILYWFAQLIGATVACLLLKFTTGG---L-STSAFSLSSGVGVG

PtTIP1;2 POPTRDRAFT_589502 (57) GLIAAAIAHAFALFVAVSVGANISGGHVNPAVTFGAFIGGNITLLRGILYWIAQLLGSTVACLLLKFTTGG---L-ETSAFALSSGVGVW

PtTIP2;1 POPTRDRAFT_676397 (55) GLVAVAVAHAFALFVGVSIAANISGGHLNPAVTFGLAIGGNITILTGLLYWIAQCLGSIAACLLLKFATSA---E-SIPTHGVASGMSAV

PcTIP4;1 (54) GLTVVALTHAFVVFAMISAGFSISGGHLNPAVTLGLAVGGHISLIRSLLYWIAQLLASALACFLLKFLTGG---S-ATPVHTLSSGMTYF

181 270

PcPIP1;1 (175) DGLGAEIMGTFVLVYTVCSATDAKRKSRDSHIPVLAPLPIGFAVFLVHLATIPITGTGINPAKSLAVAIIYDRSHAWDDQWIFWVGPLVG

PtPIP1;1 POPTRDRAFT_831918 (178) SGLGVEILGTFVLVYTVFSATDAKRSARDSHVPVLAPLPIGFAVFLVHLATIPITGTGINPARSLGAALIYNKDKAWDDHWIFWVGPFIG

PcPIP2;1 (79) VGLAAEIIGTFVLVYTVFSATDPKRSARDSHVPVLAPLPIGFAVFMVHLATIPITGTGINPARSFGAAVIYGHKQPWNDQWIFWVGPFCG

PtPIP2;8 POPTRDRAFT_836572 (154) TALGAEIIGTFVLVYTVLAATDPKRMARDSHVPVLAPLPIGFAVFVVHLALIPITGTGINPARSLGAAVVKNAKEIWDDHWIFWVGPFVG

PcTIP1;1 (143) NAVVFEIVMTFGLVYTVYATAVDPKKG---NLGTIAPICIGFIVGANILAGGAFDGASMNPARAFGPALVS---WTWENHWIYWVGPLLG

PtTIP1;2 POPTRDRAFT_589502 (143) NAFVLEIVMTFGLVYTVYATAVDPKKG---NLGIIAPIAIGFIVGANILAGGAFDGASMNPAVSFGPALVS---WTWTNHWVYWAGPLIG

PtTIP2;1 POPTRDRAFT_676397 (141) EGVVMEIVITFALVYTVYATAADPKKG---SIGIIAPIAIGFIVGANILAAGPFSGGSMNPARSFGPAVVS---GDFSQNWIYWLGPLIG

PcTIP4;1 (140) QGVIMEIVLTFSLLFTVYATAVDPKKG---NVGITAPLCVGLVVGANIFAGGPFSGASMNPARSFGPALVT---GIWKDHWVYWVGPLVG

271 300

PcPIP1;1 (265) AALASLYHMLIIRAIPFKSRI---------

PtPIP1;1 POPTRDRAFT_831918 (268) AALASLYHQIVIRAIPFKSK----------

PcPIP2;1 (169) AALAAAYHQYILRAAAIKALGSFRSNPRN-

PtPIP2;8 POPTRDRAFT_836572 (244) ALAAAVYHQYILGSGAAKALASFRSNPTS-

PcTIP1;1 (227) GGLAGVIYELFMISPEPTHQPLPSNEY---

PtTIP1;2 POPTRDRAFT_589502 (227) GGLAGLIYEFFFIG-FGNHEQLPTADY---

PtTIP2;1 POPTRDRAFT_676397 (225) GGLAGLVYGDIFIG---SYTAAPVSEDYA-

PcTIP4;1 (224) GGLAGFVYENIFIY--ETHTPLPDVEY---

**Supplemental Fig. S2b.** Multiple alignment of deduced amino acid AQP sequences of Aquaporin of *Populus trichocarpa* with *Pinus contorta.* Vector NTI (AlignX) was used to generate multiple sequence alignments. Identical residues in all sequences are shaded in dark grey, while similar residues are shaded in light grey. Conserved NPA motif is boxed. Histidine residues involved in pH sensing are pointed by arrows (Shelden *et al*., 2009).
