# Supplementary material for: Influence of water deficit on the molecular responses of Pinus contorta × Pinus banksiana mature trees to infection by the mountain pine beetle fungal associate, Grosmannia clavigera
Source: Tree Physiol. 2013 Dec 5;34(11):1220–39. doi: 10.1093/treephys/tpt101 (PMC4277265; doi:10.1093/treephys/tpt101)
Supplement: Supplementary Data [file supp_tpt101_tpt101supp_fig4.pptx]

## Slide 1
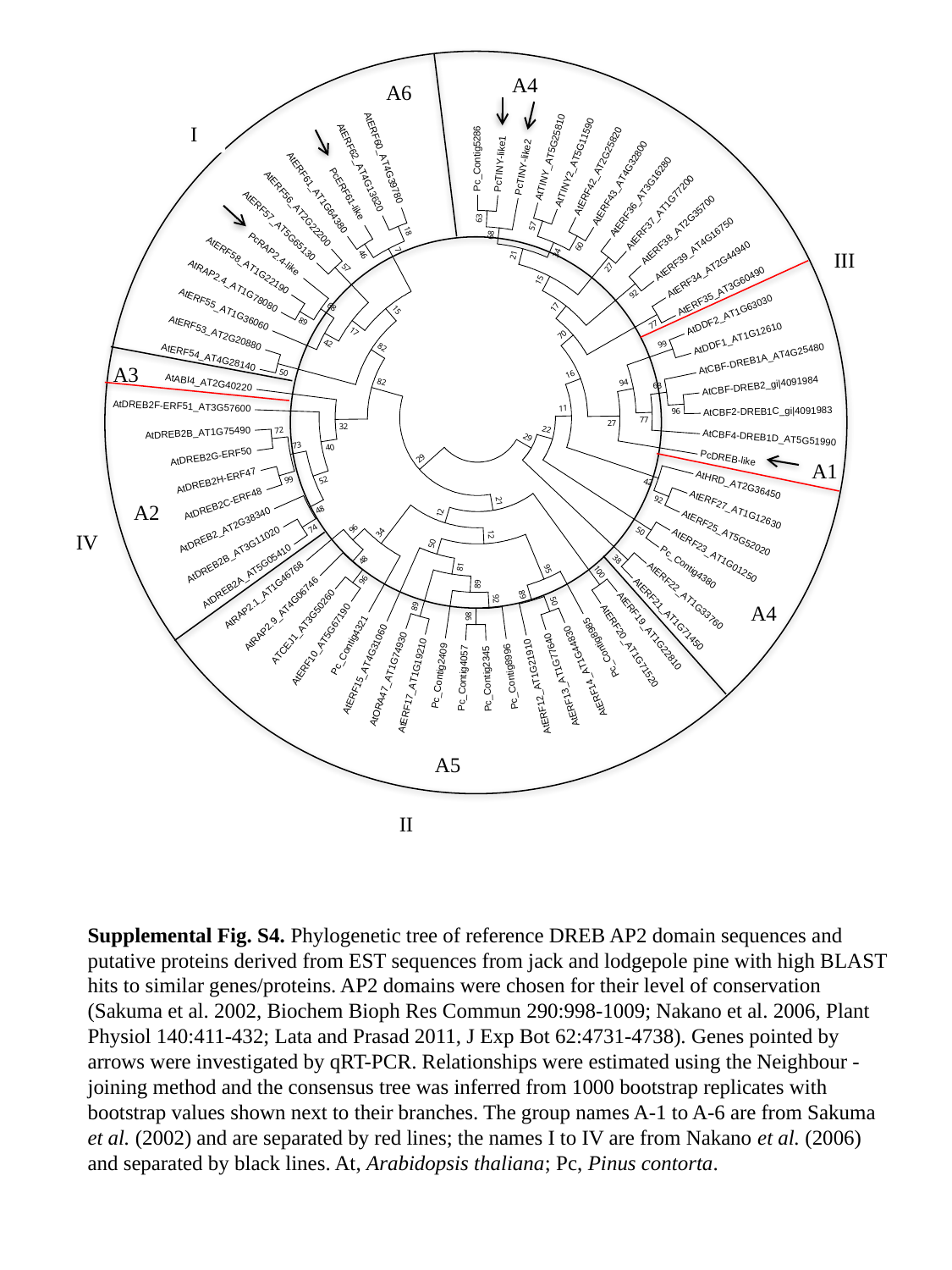

A4
A6
I
III
A3
A1
A2
IV
A4
A5
II
Supplemental Fig. S4. Phylogenetic tree of reference DREB AP2 domain sequences and putative proteins derived from EST sequences from jack and lodgepole pine with high BLAST hits to similar genes/proteins. AP2 domains were chosen for their level of conservation (Sakuma et al. 2002, Biochem Bioph Res Commun 290:998-1009; Nakano et al. 2006, Plant Physiol 140:411-432; Lata and Prasad 2011, J Exp Bot 62:4731-4738). Genes pointed by arrows were investigated by qRT-PCR. Relationships were estimated using the Neighbour -joining method and the consensus tree was inferred from 1000 bootstrap replicates with bootstrap values shown next to their branches. The group names A-1 to A-6 are from Sakuma et al. (2002) and are separated by red lines; the names I to IV are from Nakano et al. (2006) and separated by black lines. At, Arabidopsis thaliana; Pc, Pinus contorta.
