# Supplementary material for: Influence of water deficit on the molecular responses of Pinus contorta × Pinus banksiana mature trees to infection by the mountain pine beetle fungal associate, Grosmannia clavigera
Source: Tree Physiol. 2013 Dec 5;34(11):1220–39. doi: 10.1093/treephys/tpt101 (PMC4277265; doi:10.1093/treephys/tpt101)
Supplement: Supplementary Data [file supp_tpt101_tpt101supp_fig5.docx]

1 90

AtCBF1_DREB1B_gi_4091982 (1) ------------------------------------------------------------------------------------------

AtCBF3_DREB1A_gi_4091984 (1) ------------------------------------------------------------------------------------------

AtCBF2_DREB1C_gi_4091983 (1) ------------------------------------------------------------------------------------------

PcTINY-like1 (1) ------------------------------------------------------------------------------------------

PcTINY-like2 (1) ------------------------------------------------------------------------------------------

AtDREB2A_AT5G05410 (1) ------------------------------------------------------------------------------------------

AtDREB2B_AT3G11020 (1) ------------------------------------------------------------------------------------------

AtRAP2.4_AT1G78080 (1) ---------------------------------------MAAAMNLYTCSRSFQDSGGELMDALVPFIKSVSDSPSSSSAASASAFLHPS

PcRAP2.4-like (1) ------------------------------------------------------------------------------------------

PcERF61-like (1) MVGKMDLITDPQNSEGPDRQGFRFPSLVSNQNLGVFNASVQDVFEDSASSSSSPSNGDARLEGLDAWQNLRMPEASLFSGNSEVLCSSSS

AtTINY_AT5G25810 (1) ------------------------------------------------------------------------------------------

PcDREB-like (1) ------------------------------------------------------------------------------------------

91 180

AtCBF1_DREB1B_gi_4091982 (1) ------------------------------------MNSFSAFSEMFGSDYEP---QGGDYCPTLATSCPK-------------KPAGRK

AtCBF3_DREB1A_gi_4091984 (1) ------------------------------------MNSFSAFSEMFGSDYESSVSSGGDYIPTLASSCPK-------------KPAGRK

AtCBF2_DREB1C_gi_4091983 (1) ------------------------------------MNSCSAFSEMFGSDYESPVSSGGDYSPKLATSCPK-------------KPAGRK

PcTINY-like1 (1) ---------------------------------------------MRKRRGRK-INNAGEPGEVESAVCNG-------------DEKQGV

PcTINY-like2 (1) ---------------------------------------------MPKRRAEE-VNNADESGKVESAACNG-------------DEEQWV

AtDREB2A_AT5G05410 (1) -------------------MAVYDQSGDRNRTQIDTSRKRKSRSRGDGTTVAERLKRWKEYNETVEEVSTK-----KRKVPAKGSKKGCM

AtDREB2B_AT3G11020 (1) -------------------MAVYEQTGTE------QPKKRKSRARAGGLTVADRLKKWKEYNEIVEASAVKEGEKPKRKVPAKGSKKGCM

AtRAP2.4_AT1G78080 (52) AFSLPPLPGYYPDSTFLTQPFSYGSDLQQTGSLIGLNNLSSSQIHQIQSQIHHPLPPTHHNNNNSFSNLLSPK--PLLMKQSGVAGSCFA

PcRAP2.4-like (1) ------------------------------------------------------------------------------------MERRDQ

PcERF61-like (91) FFPNLPSNGFGHSDGLVWRPSFQGQNQYSGESIESAMVLYELLHVQQIQQIQQQQFQLQQHQTSAAASIHHMG---RN--PLGPRAQPMK

AtTINY_AT5G25810 (1) -------------------------------------------------------MIASESTKSWEASAVR-----------Q-ENEEEK

PcDREB-like (1) --------------------------------------------------MAEKQFVPNSPNGMVELESKK--------------RKNRA

181 270

AtCBF1_DREB1B_gi_4091982 (39) KFR---ETRHPIYRGVRQRNSGKWVSEVREPNKKTRIWLGTFQTAEMAARAHDVAALALRGRSACLNFADSAWRLRI------------P

AtCBF3_DREB1A_gi_4091984 (42) KFR---ETRHPIYRGVRRRNSGKWVCEVREPNKKTRIWLGTFQTAEMAARAHDVAALALRGRSACLNFADSAWRLRI------------P

AtCBF2_DREB1C_gi_4091983 (42) KFR---ETRHPIYRGVRQRNSGKWVCELREPNKKTRIWLGTFQTAEMAARAHDVAAIALRGRSACLNFADSAWRLRI------------P

PcTINY-like1 (32) RIK---EKRHRVYRGVRMRSWGRWVSEIREPKKKSRIWLGTFPTPEMAARAHDVAALSIKGKSAFLNFPHMASSLPR------------P

PcTINY-like2 (32) KIK---GKRHRRYRGVRMRSWGKWVSEIREPKKKSRIWLGTFSTPEMAARAHDVAALSIKGKSAFLNFPHIASSLPR------------P

AtDREB2A_AT5G05410 (67) KGKGGPENSRCSFRGVRQRIWGKWVAEIREPNRGSRLWLGTFPTAQEAASAYDEAAKAMYGPLARLNFPRSDASEVT------------S

AtDREB2B_AT3G11020 (66) KGKGGPDNSHCSFRGVRQRIWGKWVAEIREPKIGTRLWLGTFPTAEKAASAYDEAATAMYGSLARLNFPQSVGSEFT------------S

AtRAP2.4_AT1G78080 (140) YGSGVPSKPTKLYRGVRQRHWGKWVAEIRLPRNRTRLWLGTFDTAEEAALAYDKAAYKLRGDFARLNFPNLRHNGSHIG-----G---DF

PcRAP2.4-like (7) SPVAARHPMRKHYRGVRQRQWGKWVAEIRLPQNRTRLWLGTFDTAEAAALAYDRAAYRWRGECARLNFPHLFSKRYQ------------N

PcERF61-like (176) LHGSSLSKPAKLYRGVRQRHWGKWVAEIRLPRNRTRLWLGTFDTAEEAALAYDKAAYRLRGDYARLNFPHLKHHLEANSFAPWTGNSVLP

AtTINY_AT5G25810 (24) KKPVKDSGKHPVYRGVRKRNWGKWVSEIREPRKKSRIWLGTFPSPEMAARAHDVAALSIKGASAILNFPDLAGSFPR------------P

PcDREB-like (27) CDEAEGAGSYPIYRGVRRRRWGKWVSEIREPRKKKRIWLGSYDTPQMAARAHDVAALCLRGKAACLNFPDLVGMFPR------------P

271 360

AtCBF1_DREB1B_gi_4091982 (114) ESTCAKDIQKAAA-----EAALAFQDETCDTTTT------DHGLDMEET------MVEAIYTPEQSEG----------------------

AtCBF3_DREB1A_gi_4091984 (117) ESTCAKDIQKAAA-----EAALAFQDEMCDATT-------DHGFDMEET------LVEAIYTAEQSEN----------------------

AtCBF2_DREB1C_gi_4091983 (117) ESTCAKEIQKAAA-----EAALNFQDEMCHMTTD------AHGLDMEET------LVEAIYTPEQSQD----------------------

PcTINY-like1 (107) ATLCPKDIQAAAA-----LAAAEFHMPSEEDCSE------DRSLDPVGTSNAIPSDSHTVIPAESGNKESAAVVCASN--FVSSFLSGNE

PcTINY-like2 (107) ATLSPKDIQAAAA-----VAAAEFHMPSEEDCSE------DRSLDPVETNNVILSHADTVIPADSGNNEDAAAVCASNSVWVSSFLSENE

AtDREB2A_AT5G05410 (145) TSSQSEVCTVETP----GCVHVKTEDPDCESKPF------SGGVEP------MYCLENGAEEMKRGVKADKHWLSEFEHNYWSDILKEKE

AtDREB2B_AT3G11020 (144) TSSQSEVCTVENKAVVCGDVCVKHEDTDCESNPF------SQILDVREESCGTRPDSCTVGHQDMNSSLNYDLLLEFEQQYWGQVLQEKE

AtRAP2.4_AT1G78080 (222) GEYKPLHSSVDAKLEAICKSMAETQKQDKSTKSS-------KKREKK-------VSSPDLSEKVKAEEN---------------------

PcRAP2.4-like (85) SSPSSTNGRIPRLSCEKSDQKYAYNGDPVHTNVYKGPPIRITAYNGDPVPIDVYRSDPVRVSAYNGDPVRISAYSGDPVGNTVTLAESEL

PcERF61-like (266) SSVDAKLQAICQSLKQPLESMSKTEESEEISCAYENSGSLGSVRDEDAKKNDVVSVKSETCDSDSSDDS------------------TIT

AtTINY_AT5G25810 (102) SSLSPRDIQVAALKAAHMETSQSFSSSSSLTFSS------SQSSSS---------LESLVSSSATGSEE----------------LGEIV

PcDREB-like (105) SSLDPSDIQSAAA-----EAARAFNGETFSLQSS------SAHGSYRDQQNYYNALSESLRESLGPDHAEN-------------------

361 450

AtCBF1_DREB1B_gi_4091982 (165) AFYMDEETMFGMPTLLDN---MAEGMLLPPPSVQWNHNYDGEG---------DGDVSLWSY-----------------------------

AtCBF3_DREB1A_gi_4091984 (167) AFYMHDEAMFEMPSLLAN---MAEGMLLPLPSVQWNHNHEVDGD--------DDDVSLWSY-----------------------------

AtCBF2_DREB1C_gi_4091983 (168) AFYMDEEAMLGMSSLLDN---MAEGMLLPSPSVQWNYNFDVEG---------DDDVSLWSY-----------------------------

PcTINY-like1 (184) WMSIEDDIVLDLPNIVGN---MAEGLLVPPPWMLEHDNYSAADNFFDENASISAETSLWNYS----------------------------

PcTINY-like2 (186) CMTIYDDILFDLPNIVGN---MAEGLLVTPPWMVEQDGYSASDNFYDVNGSISAETSLWNYS----------------------------

AtDREB2A_AT5G05410 (219) K--QKEQGIVETCQQQQQ-----DSLSVADYGWPND----VDQSHLDSSDMFDVDELLRDLNGDDVFAGLNQDRYPGNSVANGSYRPESQ

AtDREB2B_AT3G11020 (228) KPKQEEEEIQQQQQEQQQQQLQPDLLTVADYGWPWSNDIVNDQTSWDPNECFDINELLGDLN------------EPG---------P-HQ

AtRAP2.4_AT1G78080 (277) SVSIGGSPPVTEFEESTAGSSPLSDLTFADPEEPPQ-WNETFSLEKYPSYEIDWDSILA-------------------------------

PcRAP2.4-like (175) ESSCSHESPNTKVERFIWEEEEDENWLNDLPVLEADMTWDVLSGCSDIDTEVSQTRTCHLW-----------------------------

PcERF61-like (338) ALNSAGESESRSASKSETQAETETDTLCSMPSFSASSIWAELDDYLLSIPPLDMDINWDVLS----------------------------

AtTINY_AT5G25810 (161) ELPSLGSSYDGLTQLGNEFIFSDSADLWPYPPQWSEGDYQMIPASLSQDWDLQGLYNY--------------------------------

PcDREB-like (165) -SVHEQQATGNSASTPCTELELRLNLWWPPPKIIQEKL----------------------------------------------------

451 488

AtCBF1_DREB1B_gi_4091982 (214) --------------------------------------

AtCBF3_DREB1A_gi_4091984 (217) --------------------------------------

AtCBF2_DREB1C_gi_4091983 (217) --------------------------------------

PcTINY-like1 (243) --------------------------------------

PcTINY-like2 (245) --------------------------------------

AtDREB2A_AT5G05410 (298) QSGFDPLQSLNYGIPPFQLEGKDGNGFFDDLSYLDLEN

AtDREB2B_AT3G11020 (296) SQDQNHVNSGSYDLHPLHLEPHDG-HEFNGLSSLDI--

AtRAP2.4_AT1G78080 (335) --------------------------------------

PcRAP2.4-like (236) --------------------------------------

PcERF61-like (400) --------------------------------------

AtTINY_AT5G25810 (219) --------------------------------------

PcDREB-like (202) --------------------------------------

**Supplemental Fig. S3b.** Multiple alignment of deduced amino acid sequences for selected characterized *A. thaliana* DREBs and *P. contorta* DREB-like sequences used in this study. Vector NTI (AlignX) was used to generate multiple sequence alignments. Identical residues in all sequences are shaded in dark grey, while similar residues are shaded in light grey. Amino acids interacting with GCC box are pointed by a black arrow (Allen *et al*. 1998, EMBO J. 17:5484-5496). Amino acids important for determining DRE binging specificity are pointed by a red arrow (Cao *et al*. 2001, Biochem 66:623-627; Sakuma *et al*. 2002, Biochem Biophys Res Commun 290:998-1009). Amino acids important for DNA binding/AP2 domain stability are pointed by a green arrow (Allen *et al*., 1998). The AP2 domain is indicated by a black line over the alignment.
