# Supplementary material for: Influence of water deficit on the molecular responses of Pinus contorta × Pinus banksiana mature trees to infection by the mountain pine beetle fungal associate, Grosmannia clavigera
Source: Tree Physiol. 2013 Dec 5;34(11):1220–39. doi: 10.1093/treephys/tpt101 (PMC4277265; doi:10.1093/treephys/tpt101)
Supplement: Supplementary Data [file supp_tpt101_tpt101supp_fig6.pptx]

## Slide 1
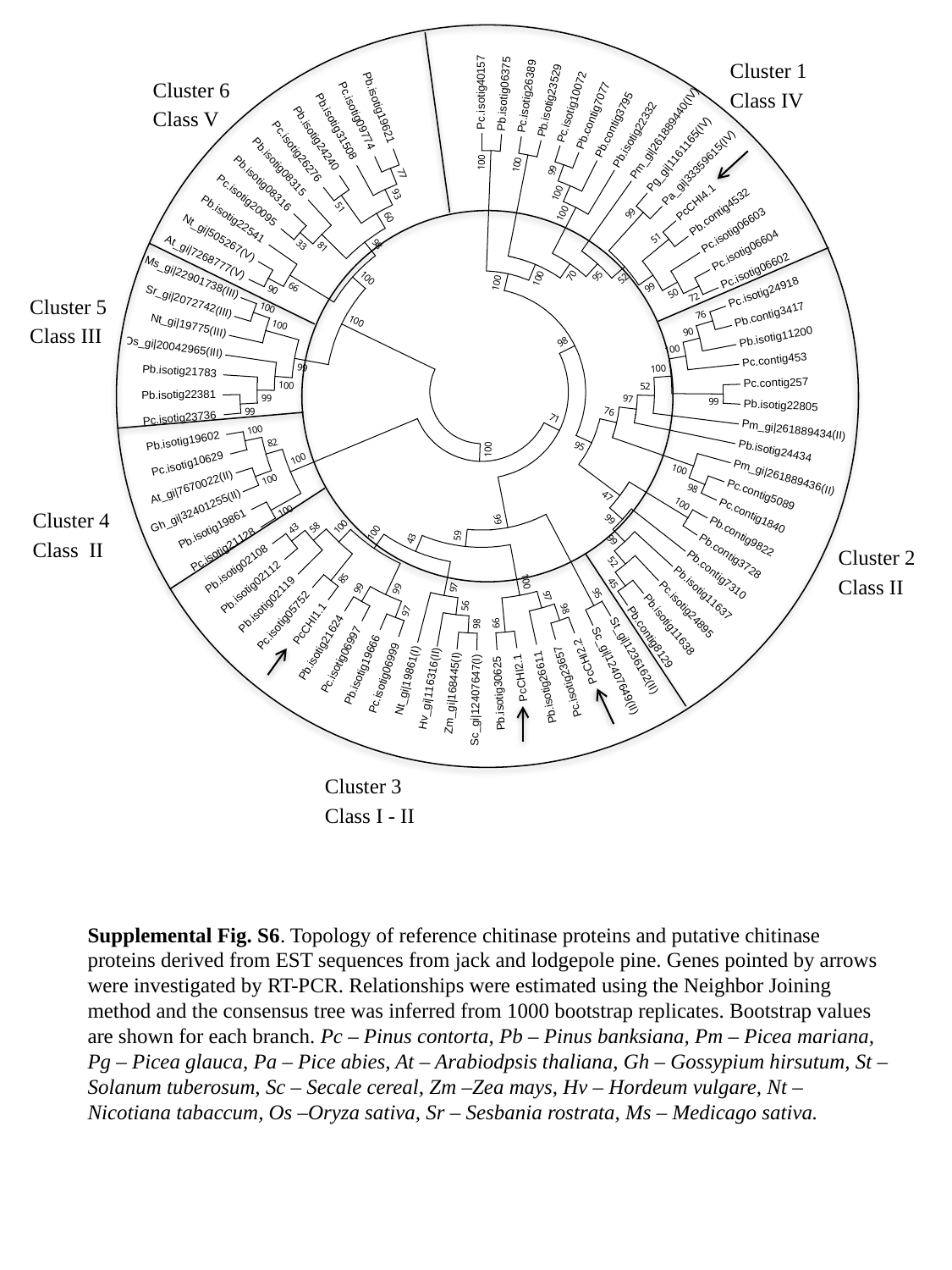

Cluster 1
Class IV
Cluster 6
Class V
Cluster 5
Class III
Cluster 4
Class II
Cluster 2
Class II
Cluster 3
Class I - II
Supplemental Fig. S6. Topology of reference chitinase proteins and putative chitinase proteins derived from EST sequences from jack and lodgepole pine. Genes pointed by arrows were investigated by RT-PCR. Relationships were estimated using the Neighbor Joining method and the consensus tree was inferred from 1000 bootstrap replicates. Bootstrap values are shown for each branch. Pc – Pinus contorta, Pb – Pinus banksiana, Pm – Picea mariana, Pg – Picea glauca, Pa – Pice abies, At – Arabiodpsis thaliana, Gh – Gossypium hirsutum, St – Solanum tuberosum, Sc – Secale cereal, Zm –Zea mays, Hv – Hordeum vulgare, Nt – Nicotiana tabaccum, Os –Oryza sativa, Sr – Sesbania rostrata, Ms – Medicago sativa.
