# Supplementary material for: Influence of water deficit on the molecular responses of Pinus contorta × Pinus banksiana mature trees to infection by the mountain pine beetle fungal associate, Grosmannia clavigera
Source: Tree Physiol. 2013 Dec 5;34(11):1220–39. doi: 10.1093/treephys/tpt101 (PMC4277265; doi:10.1093/treephys/tpt101)
Supplement: Supplementary Data [file supp_tpt101_tpt101supp_fig7.docx]

1 90

Nt_gi|19861(I) (1) ----MRLCKFTALSSLLFSLLLLSASAEQCGSQAGGARCPSGLCCSKFGWCGNTNDYCGPGNCQSQCPGGPTPTPPTPPGGGDLGSIISS

PcCHI1.1 (1) -MKSMKFCAMAIALLTMATMNMYFVSAEQCGQQAGGALCPGGLCCSKWGWCGNTDAHCG------QDCQSQCSGSTPTPGGQGVASIITE

PcCHI4.1 (1) ---------MVLVVVLVLLLAGVSVNAQNCGCASG-------LCCSKFGYCGTTSAYCGDGCQ--SGPCTSSGGGSPSGGGGSVGTIISQ

PcCHI2.1 (1) ------MARKMSMKLLLALAAVAIMS-TLCYVSEQ----------------------------------------------QGVGSIITE

PcCHI2.2 (1) MAYTNMGRRMSIMRLLLALTAVAIMSSLCCYVSAQ----------------------------------------------QGVASIISE

91 180

Nt_gi|19861(I) (87) SMFDQMLKHRNDNACQGKGFYSYNAFINAARSFPGFGTSGDTTARKREIAAFFAQTSHETTGGWATAPDGPYAWGYCWLREQGSP-GDYC

PcCHI1.1 (84) SIFNELLKHRNDAGCKASGFYTYSAFIAAANAFPSFGTTGDVATRKRELAAFFGQTSHETTGGWATAPDGAYAWGYCFKEEQGNPPAEYC

PcCHI4.1 (73) SFFNGLAGG-AASSCEGKGFYTYDAFIAAANAYSGFGTTGSADVTKRELAAFFANVMHET-GGMCYINERTPPMIYCMSSAT--------

PcCHI2.1 (38) DVFNEFLKHRNDAACQARDFYTYSAFIAATNSFSDFGNNGDLESRKRELAAFFGQTSQETTGGWATAPDGPYAWGYCFKEE---------

PcCHI2.2 (45) DVFNQFLKHRNDAACSAKGFYTYTAFIAAANSFPDFGNNGDLESRKRELAAFFGQTSQETTGGWATAPDGPYAWGYCFKDQV--------

181 270

Nt_gi|19861(I) (176) TPSGQWPCAPGRKYFGRGPIQISHNYNYGPCGRAIGVDLLNNPDLVATDPVISFKSALWFWMTPQSPKPSCHDVIIGRWQPSAGDRAANR

PcCHI1.1 (174) QATSQWSCASGKRYYGRGPVQLSWNYNYGPAGKAIGFDGINNPDIVASDATVSFKTAIWFWMTAQSPKPSCHDVMTGKWTPSGSDSAAGR

PcCHI4.1 (153) -----WPCASGKDYHGRGPLQLSWNYNYGAAGKNIGFDGVNNPEKVGQDSTISFKTAVWFWMDNSN----CHTAITSGQ-----------

PcCHI2.1 (119) --------NSGDKYHGRGPIQLTGDYNYKAAGDALGYDLINNPDLLVTDATVSFKTAVWFWMTAQAPKPSCHDVILGRWSPSNDDTAAGR

PcCHI2.2 (127) --------NSGDRYHGRGPIQLTGDYNYKAAGDALGYDLINNPDLVVNDATISFKTAVWFWMTAQSPKPSCHDVILGRWSPSATDTAAGR

271 342

Nt_gi|19861(I) (266) LPGFGVITNIINGGLECGRGTDSR-VQDRIGFYRRYCSILGVSPGDNLDCGNQRSFGNGLLVDTM-------

PcCHI1.1 (264) AAGYGAVTNIINGGLECGKGSDSR-QQDRIGFYKRYSDILGVSYGSNLDCNNQRPFGAAVQSEPRLIKTVV-

PcCHI4.1 (223) --GFGGTIKAINS-QECNGGNSGE-VNSRVNYYKNICSQLGVDPGANVSC----------------------

PcCHI2.1 (201) VPGYGLLTNIINGGMECGTGTISDRQQGRIGFYQRYCSLLGVDTGSNLDCQNQKHF----------------

PcCHI2.2 (209) VAGYGMVTDIINGGPECGTGTISDVQQGRIGFYQRYCNMLGVDVGSNLDCKNQKPFGT--------------

**Supplemental Fig. S4b.** Multiple alignment of amino acid sequence of Chitinases of *Pinus contorta and Nicotiana tabacum*. Vector NTI (AlignX) was used to generate multiple sequence alignments. Identical residues in all sequences are shaded in dark grey; while similar an conservative residues are shaded in light grey. Block lines above alignment represent regions in the tobacco chitinase used as reference: red (signal peptide), blue (chitin binding domain), orange (linker region), black (catalytic domain), green (carboxy terminal extension). Two glutamic acids involved in catalysis (Hart et al. 1995, J Mol Biol 248:402-413) are indicated by arrows.
