# Supplementary material for: Influence of water deficit on the molecular responses of Pinus contorta × Pinus banksiana mature trees to infection by the mountain pine beetle fungal associate, Grosmannia clavigera
Source: Tree Physiol. 2013 Dec 5;34(11):1220–39. doi: 10.1093/treephys/tpt101 (PMC4277265; doi:10.1093/treephys/tpt101)
Supplement: Supplementary Data [file supp_tpt101_tpt101supp_fig8.pptx]

## Slide 1
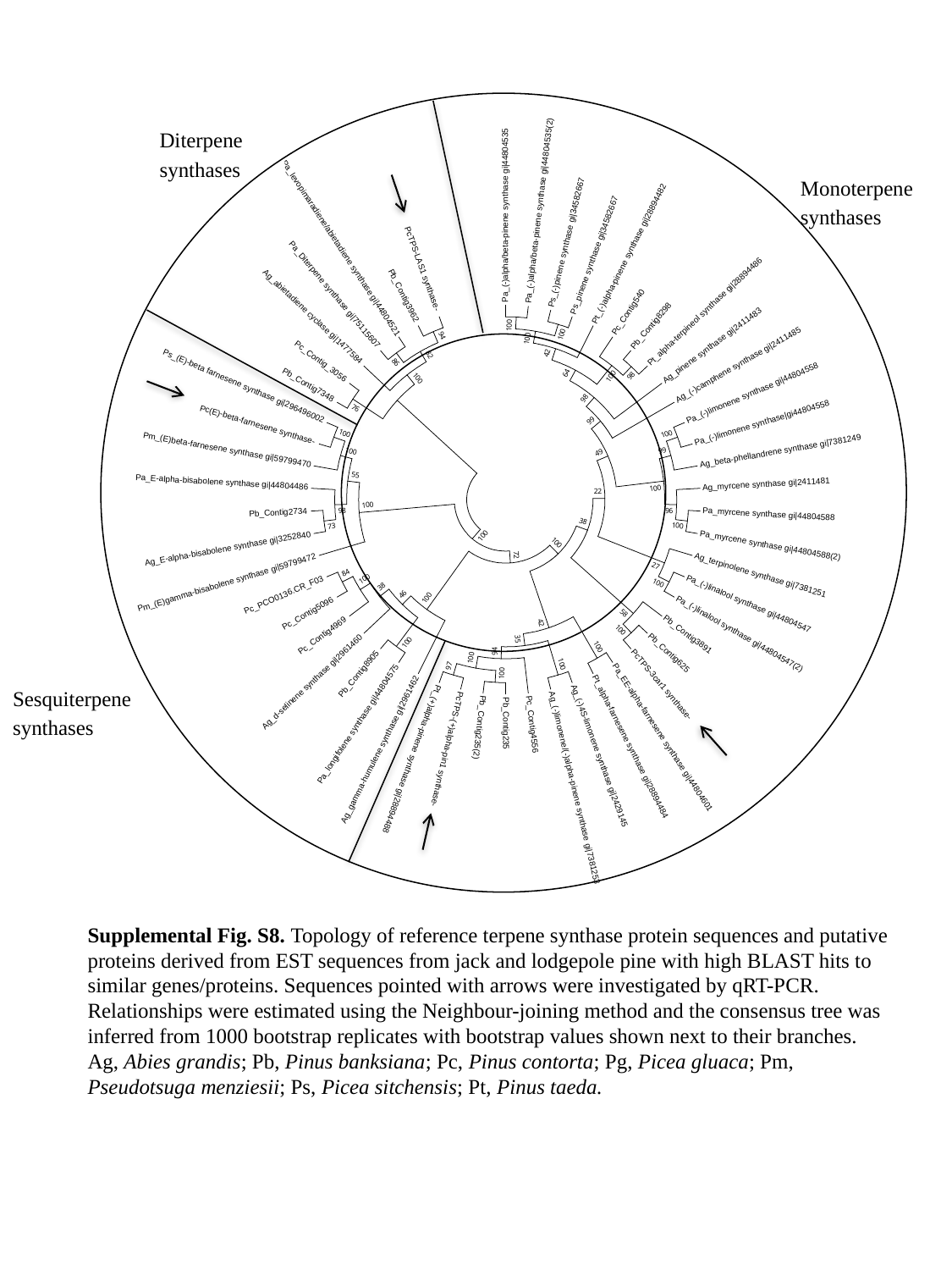

Diterpene synthases
Monoterpene synthases
Sesquiterpene synthases
Supplemental Fig. S8. Topology of reference terpene synthase protein sequences and putative proteins derived from EST sequences from jack and lodgepole pine with high BLAST hits to similar genes/proteins. Sequences pointed with arrows were investigated by qRT-PCR. Relationships were estimated using the Neighbour-joining method and the consensus tree was inferred from 1000 bootstrap replicates with bootstrap values shown next to their branches. Ag, Abies grandis; Pb, Pinus banksiana; Pc, Pinus contorta; Pg, Picea gluaca; Pm, Pseudotsuga menziesii; Ps, Picea sitchensis; Pt, Pinus taeda.
