# Supplementary material for: Influence of water deficit on the molecular responses of Pinus contorta × Pinus banksiana mature trees to infection by the mountain pine beetle fungal associate, Grosmannia clavigera
Source: Tree Physiol. 2013 Dec 5;34(11):1220–39. doi: 10.1093/treephys/tpt101 (PMC4277265; doi:10.1093/treephys/tpt101)
Supplement: Supplementary Data [file supp_tpt101_tpt101supp_fig9.docx]

1 80

Pa_(+)-3-carene_synthase_gi_29293034 (1) --------------------------------------------------------------------------------

Pt_(+)alpha-pinene_synthase_gi_28894488 (1) --------------------------------------------------------------------------------

Pc_TPS-(+)alpha-pin1_synthase-like (1) --------------------------------------------------------------------------------

Pa_Diterpene_synthase_gi_75115607 (1) MALLSSSLSSQIPTGAHHLTLNAYANTQCIPHFFSTLNAGTSAGKRSSLYLRWGKGSNKIIACVGEDSLSAPTLVKREFP

Pc_TPS-LAS1_synthase-like (1) --------------------------------------------------------------------------------

Ps_(E)-beta_farnesene_synthase_gi_296496002 (1) -------------------------------------------------MASASVASSTLPSGLSSSSSSSVIRRTANPH

Pc_(E)-beta-farnesene_synthase-like (1) --------------------------------------------------------------------------------

Pc_TPS-3car1_synthase-like (1) --------------------------------------------------------------------------------

81 160

Pa_(+)-3-carene_synthase_gi_29293034 (1) --------------------------------------------------------------------------------

Pt_(+)alpha-pinene_synthase_gi_28894488 (1) --------------------------------------------------------------------------------

Pc_TPS-(+)alpha-pin1_synthase-like (1) --------------------------------------------------------------------------------

Pa_Diterpene_synthase_gi_75115607 (81) PGFWKDHVIDSLTSSHKVAASDEKRIETLISEIKNMFRSMGYGDTNPSAYDTAWVARIPAVDGSEQPEFPETLEWILQNQ

Pc_TPS-LAS1_synthase-like (1) --------------------------------------------------------------------------------

Ps_(E)-beta_farnesene_synthase_gi_296496002 (32) PNVWDYDFVQSLQSPYTDSCYGERAETLISEIKLVLTGEGDALMITPSAYDTAWVARVPAIDGSSRPQFPQTVDWILKNQ

Pc_(E)-beta-farnesene_synthase-like (1) --------------------------------------------------------------------------------

Pc_TPS-3car1_synthase-like (1) --------------------------------------------------------------------------------

161 240

Pa_(+)-3-carene_synthase_gi_29293034 (1) ----------------------------------------------------------MSVISILPLASKSCLYKSLMSS

Pt_(+)alpha-pinene_synthase_gi_28894488 (1) ----------------------------------------------------------MALVSAVPLNSKLCLRRTLFGF

Pc_TPS-(+)alpha-pin1_synthase-like (1) --------------------------------------------------------------------------------

Pa_Diterpene_synthase_gi_75115607 (161) LKDGSWGEGFYFLAYDRILATLACIITLTLWRTGEIQVQKGIEFFKTQAG-KIEDEADSHRPSGFEIVFPAMLKEAKVLG

Pc_TPS-LAS1_synthase-like (1) --------------------------------------------------------------------------------

Ps_(E)-beta_farnesene_synthase_gi_296496002 (112) LKDGSWGTESHFLLSDRLLATLSCVLALLKWKVGHVQVEHGIEFIKSNLEAIKDESNQDSCVTDFEIIFPSLIGEAQSLH

Pc_(E)-beta-farnesene_synthase-like (1) --------------------------------------------------------------------------------

Pc_TPS-3car1_synthase-like (1) --------------------------------------------------------------------------------

241 320

Pa_(+)-3-carene_synthase_gi_29293034 (23) THELKALCRPIATLGMCRRGKSVMAS--KSTSLTTAVS-DDGVQRRIGDHHSNLWDDNFIQSLSSPYGASSYGERAERLI

Pt_(+)alpha-pinene_synthase_gi_28894488 (23) SHELKAIHSTVPNLGMCRGGKSIAPS--MSMSSTTSVSNEDGVPRRIAGHHSNLWDDDSIASLSTSYEAPSYRKRADKLI

Pc_TPS-(+)alpha-pin1_synthase-like (1) --------------------------------------------------------------------------------

Pa_Diterpene_synthase_gi_75115607 (240) LDLPYELPFIKQIIEKREAKLERLPTNILYALPTTLLYSLEGLQEIVDWQKIIKLQSKDGSFLSSPASTAAVFMRTGNKK

Pc_TPS-LAS1_synthase-like (1) --------------------------------------------------------------------------------

Ps_(E)-beta_farnesene_synthase_gi_296496002 (192) LGLPYNLPYVRMLQMKRREKLANLPR--DEIHGGTLLSSLEGIQDTVEWERIMEVQSQDGSFSGSPASTACVFMHTGDMK

Pc_(E)-beta-farnesene_synthase-like (1) --------------------------------------------------------------------------------

Pc_TPS-3car1_synthase-like (1) --------------------------------------------------------------------------------

321 400

Pa_(+)-3-carene_synthase_gi_29293034 (100) GEVKEIFNSLSRTDGELVSHVDDLLQHLSMVDNVERLGIDRHFQTEIKVSLDYVYSYWSEKGIGSGRDIVCTDLNTTALG

Pt_(+)alpha-pinene_synthase_gi_28894488 (101) GEVKNIFDLMSVEDGVFTSPLSDLHHRLWMVDSVERLGIDRHFKDEINSALDHVYSYWTEKGIGRGRESGVTDLNSTALG

Pc_TPS-(+)alpha-pin1_synthase-like (1) --------------------------------------------------------------------------------

Pa_Diterpene_synthase_gi_75115607 (320) CLEFLNFVLKKFGNHVPCHYPLDLFERLWAVDTIERLGIDRHFKEEIKDALDYVYSHWDERGIGWARENPVPDIDDTAMG

Pc_TPS-LAS1_synthase-like (1) --------------------------------------------------------------------------------

Ps_(E)-beta_farnesene_synthase_gi_296496002 (270) CLQFLNSVLTKFGISVPFLYPVDLLEGLLMVDNIVRLGIDRHFEKEIKEILDCVYRHWNES------LNPVADLEITALG

Pc_(E)-beta-farnesene_synthase-like (1) --------------------------------------------------------------------------------

Pc_TPS-3car1_synthase-like (1) -----------------------------MVDNVERLGIDRHFQTEIKVALDYVYRYWSEEGIGCGRDSAFTDLNTTALA

401 480

Pa_(+)-3-carene_synthase_gi_29293034 (180) FRILRLHGYTVFPDVFEHFKDQMGRIACSDNHTERQISSILNLFRASLIAFPGEKVMEEAEIFSATYLKEALQT------

Pt_(+)alpha-pinene_synthase_gi_28894488 (181) LRTLRLHGYTVSSHVLDHFKNEKGQFTCSAIQTEGEIRDVLNLFRASLIAFPGEKIMEAAEIFSTMYLKDALQK------

Pc_TPS-(+)alpha-pin1_synthase-like (1) --------------------------------------------------------------------------------

Pa_Diterpene_synthase_gi_75115607 (400) LRILRLHGYNVSSDVLKTFRDENGEFFCFLGQTQRGVTDMLNVNRCSHVAFPGETIMEEAKTCTERYLRNALEDVGAFDK

Pc_TPS-LAS1_synthase-like (1) --------------------------------------------------------------------------------

Ps_(E)-beta_farnesene_synthase_gi_296496002 (344) FRLLRLHRYSVTPAVFENFKDADGHFFGSTSQFNKNVASMLSLYRASQLAFPGETILDEARDFATKYLREALEKSEIFTA

Pc_(E)-beta-farnesene_synthase-like (1) ---------------------------------------MLSLYRASQLAFPGETILDEARDFATKYLREALEKSEIFTA

Pc_TPS-3car1_synthase-like (52) FRIFRLHGYTVSSDVFEHFKDQKGQFAASANDTELQTRSVFNLFRASLIAFPEEKVLEEAEKFAAAYLKAALQT------

481 560

Pa_(+)-3-carene_synthase_gi_29293034 (254) -IPVSSLSQEIQYVLQYRWHSNLPRLEARTYIDILQEN------TKNQMLDVNTKKVLELAKLEFNIFHSLQQNELKSVS

Pt_(+)alpha-pinene_synthase_gi_28894488 (255) -IPPSGLSQEIEYLLEFGWHTNLPRMETRMYIDVFGED------TTFETPYLIREKLLELAKLEFNIFHSLVKRELQSLS

Pc_TPS-(+)alpha-pin1_synthase-like (1) --------------------------------------------------------------------------------

Pa_Diterpene_synthase_gi_75115607 (480) WALKKNIRGEVEYALKYPWHRSMPRLEARSYIEHYGPNDVWLGKTMYMMPYISNEKYLELAKLDFNHVQSLHQKELRDLR

Pc_TPS-LAS1_synthase-like (1) --------------------------------------------------------------------------------

Ps_(E)-beta_farnesene_synthase_gi_296496002 (424) WNNKQNLSQEIQYELENSWHASVSRVEAKRYCQGYSSDYARLAKSVYKLPRANNQKILELAKLDFNIIQAIHQKEMKNVT

Pc_(E)-beta-farnesene_synthase-like (42) WNNKHNLSQEIQYELENSWHASVSRVEAKRYCQGYSSDYARLAKSVHKLPRVS-QNILELAKLDFNIIQAIHQKEMKNVT

Pc_TPS-3car1_synthase-like (126) -LPVSGLSREIQYVFDYRWHSNLPRLEARSYIDILADN------TISGTPDANTKKLLELAKLEFNIFHSVQQKELQCLW

561 640

Pa_(+)-3-carene_synthase_gi_29293034 (327) RWWKESGFPDLNFIRHRHVEFYTLVSGIDMEPKHCTFRLSFVKMCHLITVLDDMYDTFGTIDELRLFTAAVKRWDPSTTE

Pt_(+)alpha-pinene_synthase_gi_28894488 (328) RWWKDYGFPEITFSRHRHVEYYTLAACIANDPKHSAFRLGFGKISHMITILDDIYDTFGTMEELKLLTAAFKRWDPSSIE

Pc_TPS-(+)alpha-pin1_synthase-like (1) --------------------------------------------------------------------------------

Pa_Diterpene_synthase_gi_75115607 (560) RWWTSSGFTELKFTRERVTEIYFSPASFMFEPEFATCRAVYTKTSNFTVILDDLYDAHGTLDDLKLFSDSVKKWDLSLVD

Pc_TPS-LAS1_synthase-like (1) -------------------------------------RAVYTKTSNFTVILDDLYDAHGTLDNLKLFSESVKRWDLSLVD

Ps_(E)-beta_farnesene_synthase_gi_296496002 (504) SWFKHSEFPLLPFGRERPVECFFLVAAGTYEPQYAKCRFLFSKVACLNTVLDDMYDTYGTLDELKLFTEAVRRWDLSLTE

Pc_(E)-beta-farnesene_synthase-like (121) TWFKHSEFPLLPFGRERPVECFFIVAAGTYEPQYAKCRFLFSKVACLNTVLDDMYDTYGTL-ELKLFTEAVRRWDLSLTE

Pc_TPS-3car1_synthase-like (199) RWWKEWGCPELTFIRHRYVEFYTLVSGIDMVPEHATFRLSCVKTCHLITILDDMYDTFGTIDELRLFTAAVKRWDPSATE

641 720

Pa_(+)-3-carene_synthase_gi_29293034 (407) CLPEYMKGVYTVLYETVNEMAQEAQKSQGRDTLSYVRQALEAYIGAYHKEAEWISSGYLPTFDEYFENGKVSSGHRIATL

Pt_(+)alpha-pinene_synthase_gi_28894488 (408) CLPDYMKGVYMAVYDNINEMAREAQKIQGWDTVSYARKSWEAFIGAYIQEAKWISSGYLPTFDEYLENGKVSFGSRITTL

Pc_TPS-(+)alpha-pin1_synthase-like (1) -----------------------------------------------------------------------------GTL

Pa_Diterpene_synthase_gi_75115607 (640) RMPQDMKICFMGFYNTFNEIAEEGRKRQGRDVLGYIRNVWEIQLEAYTKEAEWSAARYVPSFDEYIDNASVSIALGTVVL

Pc_TPS-LAS1_synthase-like (44) QMPQDMKICFKGFYNTFNEIAEEGRKRQGRDVLGYIQKVWEVQLEAYTKEAEWSAVRYVPSYDEYIGNASVSIALGTVVL

Ps_(E)-beta_farnesene_synthase_gi_296496002 (584) SLPDYMKLCYKIFYEIVHEVVREAEKLQGRELLSFFRKGWEEYLLGYYEEAEWLASEYVPSLEEYIRNGIISIGQRILLV

Pc_(E)-beta-farnesene_synthase-like (200) SLPDYMKLCYKIFYEIVHEVVREAEKLQGRELLSFFRKGWEEYLLGYYEEAEWLASEYVPSLEEYIRNGIISIGQRILLV

Pc_TPS-3car1_synthase-like (279) CLPEYMKGVYMVLYETVNEMAKEAQKSQGRDTLGYVRQALEDYIGSYLKEAEWIATGYVPTFQEYFENGKLSSGHRIATL

721 800

Pa_(+)-3-carene_synthase_gi_29293034 (487) QPTFMLD-IPFPHHVLQEIDFPSK--FNDFACSILRLRGDTRCYQADRARGEEASCISCYMKDNPGSTQEDALNHINNMI

Pt_(+)alpha-pinene_synthase_gi_28894488 (488) EPMLTLG-FPLPPRILQEIDFPSK--FNDLICAILRLKGDTQCYKADRARGEEASAVSCYMKDHPGITEEDAVNQVNAMV

Pc_TPS-(+)alpha-pin1_synthase-like (4) EPMLTLG-FPLPPRILQEIDFPSK--FNDLTCAILRLKGDTQCYKADRARGEEASAVSCYMKDHPGITEEDAVNQVNAMV

Pa_Diterpene_synthase_gi_75115607 (720) ISALFTG-EILTDDVLSKIGRGSR--FLQLMGLTGRLVNDTKTYEAERGQGEVASAVQCYMKDHPEISEEEALKHVYTVM

Pc_TPS-LAS1_synthase-like (124) ISALFTG-EILTDDILSKIGRDSR--FLYLMGLTGRLVNDTKTYQAERGQGEVASAVQCYMKDHPEISEEEALKHVYTIM

Ps_(E)-beta_farnesene_synthase_gi_296496002 (664) SGVLLMEGQILSQEALEKLDYPGRRVLTELNCIITRLADDIHTYKAEKARGELASSIECYMKEHPGSTEEVAVNYMYSLL

Pc_(E)-beta-farnesene_synthase-like (280) SGVLLMEGQILSQEALEELDYPGRRVLTELNCIITRLADDIHTYKAEKARGELASSIECYMKEHPGSTEEVAVNYMYSLL

Pc_TPS-3car1_synthase-like (359) QPILTLS-IPFPHHILQEIDFPSK--FNDYAASILRLRGDTRCYKADSARGEEASCISCYMRDNPGSTQEDALNHINGMI

801 869

Pa_(+)-3-carene_synthase_gi_29293034 (564) EETIKKLNWELLKPDN----NVPISSKKHAFDINRGLHHFYNYRDGYTVAS-NETKNLVIKTVLEPVPM

Pt_(+)alpha-pinene_synthase_gi_28894488 (565) DNLTKELNWELLRPDS----GVPISYKKVAFDICRVFHYGYKYRDGFSVAS-IEIKNLVTRTVVETVPL

Pc_TPS-(+)alpha-pin1_synthase-like (81) DNLTKELNWELLRPDS----GVPISYKKVAFDICRVFHYGYKYRDGFSVAS-VEIKNLVTRTVVETVPL

Pa_Diterpene_synthase_gi_75115607 (797) ENALDELNREFVNNRE-----VPDSCRRLVFETARIMQLFYMDGDGLTLSHETEIKEHVKNCLFQPVA-

Pc_TPS-LAS1_synthase-like (201) DNALDELNREFVNNRD-----VPDTCRRLVFETARIMQLFYMDGDGLTLSHNMEIKEHVKNCLFQPVA-

Ps_(E)-beta_farnesene_synthase_gi_296496002 (744) EPAVKELTWEFLKPDDTSDVDIPFQCKKMLMEETRVTMVIFKEGDGFGISK-TKIKDYIKECLIEPLPL

Pc_(E)-beta-farnesene_synthase-like (360) EPAVKELTWEFLKPHDTADVDIPFQCKKMLMEETRVTMVIFKEGDGFGIFK-TKIKDYIKECLIEPLPL

Pc_TPS-3car1_synthase-like (436) EDMIKKLNWEFLRPDN----NAPISSKKHAFNISRGLHHFYNYRDGYSVAS-KETKDLVIKTVLEPVLM

**Supplemental Fig. S5b.** Multiple alignment of amino acid sequence of Terpene synthases of *Picea abies, Picea taeda, Pinus sylvestris* and *Pinus contorta*. Vector NTI (AlignX) was used to generate multiple sequence alignments. Most conservative region identified between all the terpense synthases is boxed. Identical residues in all sequences are shaded in dark grey, while similar residues are shaded in light grey.
