# Supplementary material for: Influence of water deficit on the molecular responses of Pinus contorta × Pinus banksiana mature trees to infection by the mountain pine beetle fungal associate, Grosmannia clavigera
Source: Tree Physiol. 2013 Dec 5;34(11):1220–39. doi: 10.1093/treephys/tpt101 (PMC4277265; doi:10.1093/treephys/tpt101)
Supplement: Supplementary Data [file supp_tpt101_tpt101supp_File1.docx]

Supplemental File S1. Sequences used in phylogenetic analyses. GenBank accession numbers are included for sequences from other species retrieved from NCBI.

>PcPIP1;1

MEEVGVGTSQYSERQPLGTSAQTDRESKDYYEPEPAPLFEPEELRSWSFWRAGIAEFMATFMLVYITLLTIMGVKRSPTMCDSVGIQGIAWSFGAAVFALVYCTAGISGGHINPAITFGLFLARKLSLTRTVFYMICQCLGAIFGAGVVKGMQKGMYEVEGGGANIVAHGYTKGDGLGAEIMGTFVLVYTVCSATDAKRKSRDSHIPVLAPLPIGFAVFLVHLATIPITGTGINPAKSLAVAIIYDRSHAWDDQWIFWVGPLVGAALASLYHMLIIRAIPFKSRI

>PcTIP1;1

MPFGGIAVGRPEEATHPEALKAALAEGISTLIFVFAGEGSGMAFDKLTSDASTTPAGLVAVALAHALGLFVAVAVGANISGGHVNPAVTFGAFVGGHITLLRGILYWFAQLIGATVACLLLKFTTGGLSTSAFSLSSGVGVGNAVVFEIVMTFGLVYTVYATAVDPKKGNLGTIAPICIGFIVGANILAGGAFDGASMNPARAFGPALVSWTWENHWIYWVGPLLGGGLAGVIYELFMISPEPTHQPLPSNEY

>PcTIP4;1

MAKIALGNRDEPARPDCVRAVFVELICTFLFVFAGVGSAMAMDQMSVPANSPAGLTVVALTHAFVVFAMISAGFSISGGHLNPAVTLGLAVGGHISLIRSLLYWIAQLLASALACFLLKFLTGGSATPVHTLSSGMTYFQGVIMEIVLTFSLLFTVYATAVDPKKGNVGITAPLCVGLVVGANIFAGGPFSGASMNPARSFGPALVTGIWKDHWVYWVGPLVGGGLAGFVYENIFIYETHTPLPDVEY

>PcPIP2;1

MIFVLVYCTAGSGGHINPAVTFGLFLARKVSLPRAVLYMIAQCLGAICGTGLVKAFQKSFYDKYGGGANYVHNGYTKGVGLAAEIIGTFVLVYTVFSATDPKRSARDSHVPVLAPLPIGFAVFMVHLATIPITGTGINPARSFGAAVIYGHKQPWNDQWIFWVGPFCGAALAAAYHQYILRAAAIKALGSFRSNPRN

>AtPIP1;1 AT3G61430

MEGKEEDVRVGANKFPERQPIGTSAQSDKDYKEPPPAPFFEPGELSSWSFWRAGIAEFIATFLFLYITVLTVMGVKRSPNMCASVGIQGIAWAFGGMIFALVYCTAGISGGHINPAVTFGLFLARKLSLTRALYYIVMQCLGAICGAGVVKGFQPKQYQALGGGANTVAHGYTKGSGLGAEIIGTFVLVYTVFSATDAKRNARDSHVPILAPLPIGFAVFLVHLATIPITGTGINPARSLGAAIIYNKDHSWDDHWVFWVGPFIGAALAALYHVVVIRAIPFKSRS

>At PIP1;2 AT2G45960

MEGKEEDVRVGANKFPERQPIGTSAQSDKDYKEPPPAPLFEPGELASWSFWRAGIAEFIATFLFLYITVLTVMGVKRSPNMCASVGIQGIAWAFGGMIFALVYCTAGISGGHINPAVTFGLFLARKLSLTRAVYYIVMQCLGAICGAGVVKGFQPKQYQALGGGANTIAHGYTKGSGLGAEIIGTFVLVYTVFSATDAKRNARDSHVPILAPLPIGFAVFLVHLATIPITGTGINPARSLGAAIIFNKDNAWDDHWVFWVGPFIGAALAALYHVIVIRAIPFKSRS

>At PIP1;3 AT1G01620

MEGKEEDVRVGANKFPERQPIGTSAQTDKDYKEPPPAPFFEPGELSSWSFYRAGIAEFIATFLFLYITVLTVMGVKRAPNMCASVGIQGIAWAFGGMIFALVYCTAGISGGHINPAVTFGLFLARKLSLTRAVFYIVMQCLGAICGAGVVKGFQPNPYQTLGGGANTVAHGYTKGSGLGAEIIGTFVLVYTVFSATDAKRSARDSHVPILAPLPIGFAVFLVHLATIPITGTGINPARSLGAAIIYNKDHAWDDHWIFWVGPFIGAALAALYHQLVIRAIPFKSRS

>At PIP1;4 AT4G00430

MEGKEEDVRVGANKFPERQPIGTSAQSTDKDYKEPPPAPLFEPGELSSWSFYRAGIAEFIATFLFLYITVLTVMGVKRAPNMCASVGIQGIAWAFGGMIFALVYCTAGISGGHINPAVTFGLFLARKLSLTRAVFYMIMQCLGAICGAGVVKGFQPTPYQTLGGGANTVAHGYTKGSGLGAEIIGTFVLVYTVFSATDAKRSARDSHVPVWTPLLVPILAPLPIGFAVFLVHLATIPITGTGINPARSLGAAIIYNKDHSWDDHWIFWVGPFIGAALAALYHQIVIRAIPFKSKS

>At PIP1;5 AT4G23400

MEGKEEDVNVGANKFPERQPIGTAAQTESKDYKEPPPAPFFEPGELKSWSFYRAGIAEFIATFLFLYVTVLTVMGVKRAPNMCASVGIQGIAWAFGGMIFALVYCTAGISGGHINPAVTFGLFLARKLSLTRALFYIVMQCLGAICGAGVVKGFQPGLYQTNGGGANVVAHGYTKGSGLGAEIVGTFVLVYTVFSATDAKRSARDSHVPILAPLPIGFAVFLVHLATIPITGTGINPARSLGAAIIYNKDHAWDDHWIFWVGPFIGAALAALYHQIVIRAIPFKSKT

>At PIP2;1 AT3G53420

MAKDVEAVPGEGFQTRDYQDPPPAPFIDGAELKKWSFYRAVIAEFVATLLFLYITVLTVIGYKIQSDTDAGGVDCGGVGILGIAWAFGGMIFILVYCTAGISGGHINPAVTFGLFLARKVSLPRALLYIIAQCLGAICGVGFVKAFQSSYYTRYGGGANSLADGYSTGTGLAAEIIGTFVLVYTVFSATDPKRSARDSHVPVLAPLPIGFAVFMVHLATIPITGTGINPARSFGAAVIYNKSKPWDDHWIFWVGPFIGAAIAAFYHQFVLRASGSKSLGSFRSAANV

>At PIP2;2 AT2G37170

MAKDVEGPEGFQTRDYEDPPPTPFFDADELTKWSLYRAVIAEFVATLLFLYITVLTVIGYKIQSDTKAGGVDCGGVGILGIAWAFGGMIFILVYCTAGISGGHINPAVTFGLFLARKVSLIRAVLYMVAQCLGAICGVGFVKAFQSSYYDRYGGGANSLADGYNTGTGLAAEIIGTFVLVYTVFSATDPKRNARDSHVPVLAPLPIGFAVFMVHLATIPITGTGINPARSFGAAVIYNKSKPWDDHWIFWVGPFIGAAIAAFYHQFVLRASGSKSLGSFRSAANV

>At PIP2;3 AT2G37180

MAKDVEGPDGFQTRDYEDPPPTPFFDAEELTKWSLYRAVIAEFVATLLFLYVTVLTVIGYKIQSDTKAGGVDCGGVGILGIAWAFGGMIFILVYCTAGISGGHINPAVTFGLFLARKVSLIRAVLYMVAQCLGAICGVGFVKAFQSSHYVNYGGGANFLADGYNTGTGLAAEIIGTFVLVYTVFSATDPKRNARDSHVPVLAPLPIGFAVFMVHLATIPITGTGINPARSFGAAVIFNKSKPWDDHWIFWVGPFIGATIAAFYHQFVLRASGSKSLGSFRSAANV

>At PIP2;4 AT5G60660

MAKDLDVNESGPPAARDYKDPPPAPFFDMEELRKWPLYRAVIAEFVATLLFLYVSILTVIGYKAQTDATAGGVDCGGVGILGIAWAFGGMIFVLVYCTAGISGGHINPAVTVGLFLARKVSLVRTVLYIVAQCLGAICGCGFVKAFQSSYYTRYGGGANELADGYNKGTGLGAEIIGTFVLVYTVFSATDPKRNARDSHVPVLAPLPIGFAVFMVHLATIPITGTGINPARSFGAAVIYNNEKAWDDQWIFWVGPMIGAAAAAFYHQFILRAAAIKALGSFGSFGSFRSFA

>At PIP2;5 AT3G54820

MTKEVVGDKRSFSGKDYQDPPPEPLFDATELGKWSFYRALIAEFIATLLFLYVTIMTVIGYKSQTDPALNPDQCTGVGVLGIAWAFGGMIFILVYCTAGISGGHINPAVTFGLLLARKVTLVRAVMYMVAQCLGAICGVALVKAFQSAYFTRYGGGANGLSDGYSIGTGVAAEIIGTFVLVYTVFSATDPKRSARDSHVPVLAPLPIGFAVFIVHLATIPITGTGINPARSLGAAIIYNKDKAWDHHWIFWVGPFAGAAIAAFYHQFVLRAGAIKALGSFRSQPHV

>At PIP2;6 AT2G39010

MTKDELTEEESLSGKDYLDPPPVKTFEVRELKKWSFYRAVIAEFIATLLFLYVTVLTVIGFKSQTDINAGGGACASVGLLGISWAFGGMIFILVYCTAGISGGHINPAVTFGLFLASKVSLVRAVSYMVAQCLGATCGVGLVKVFQSTYYNRYGGGANMLSDGYNVGVGVGAEIIGTFVLVYTVFSATDPKRNARDSHIPVLAPLPIGFSVFMVHLATIPITGTGINPARSFGAAVIYNNQKAWDDQWIFWVGPFVGAAIAAFYHQFVLRAGAMKAYGSVRSQLHELHA

>At PIP2;7 AT4G35100

SKEVSEEGKTHHGKDYVDPPPAPLLDMGELKSWSFYRALIAEFIATLLFLYVTVATVIGHKKQTGPCDGVGLLGIAWAFGGMIFVLVYCTAGISGGHINPAVTFGLFLARKVSLVRALGYMIAQCLGAICGVGFVKAFMKTPYNTLGGGANTVADGYSKGTALGAEIIGTFVLVYTVFSATDPKRSARDSHIPVLAPLPIGFAVFMVHLATIPITGTGINPARSFGAAVIYNNEKAWDDQGIFWVGPFLGALAAAAYHQYILRASAIKALGSFRSNATN

>At PIP2;8 AT2G16850

MSKEVSEEGRHGKDYVDPPPAPLLDMAELKLWSFYRAIIAEFIATLLFLYVTVATVIGHKNQTGPCGGVGLLGIAWAFGGMIFVLVYCTAGISGGHINPAVTFGLFLARKVSLPRAVAYMVAQCLGAICGVGLVKAFMMTPYKRLGGGANTVADGYSTGTALGAEIIGTFVLVYTVFSATDPKRSARDSHVPVLAPLPIGFAVFMVHLATIPITGTGINPARSFGAAVIYNNEKAWDDHWIFWVGPFVGALAAAAYHQYILRAAAIKALASFRSNPTN

>At TIP1;1 AT2G36830

MPIRNIAIGRPDEATRPDALKAALAEFISTLIFVVAGSGSGMAFNKLTENGATTPSGLVAAAVAHAFGLFVAVSVGANISGGHVNPAVTFGAFIGGNITLLRGILYWIAQLLGSVVACLILKFATGGLAVPAFGLSAGVGVLNAFVFEIVMTFGLVYTVYATAIDPKNGSLGTIAPIAIGFIVGANILAGGAFSGASMNPAVAFGPAVVSWTWTNHWVYWAGPLVGGGIAGLIYEVFFINTTHEQLPTTDY

>At TIP1;2 AT3G26520

MPTRNIAIGGVQEEVYHPNALRAALAEFISTLIFVFAGSGSGIAFNKITDNGATTPSGLVAAALAHAFGLFVAVSVGANISGGHVNPAVTFGVLLGGNITLLRGILYWIAQLLGSVAACFLLSFATGGEPIPAFGLSAGVGSLNALVFEIVMTFGLVYTVYATAVDPKNGSLGTIAPIAIGFIVGANILAGGAFSGASMNPAVAFGPAVVSWTWTNHWVYWAGPLIGGGLAGIIYDFVFIDENAHEQLPTTDY

>AtTIP1;3 AT4G01470

MPINRIAIGTPGEASRPDAIRAAFAEFFSMVIFVFAGQGSGMAYGKLTGDGPATPAGLVAASLSHAFALFVAVSVGANVSGGHVNPAVTFGAFIGGNITLLRAILYWIAQLLGAVVACLLLKVSTGGMETAAFSLSYGVTPWNAVVFEIVMTFGLVYTVYATAVDPKKGDIGIIAPLAIGLIVGANILVGGAFDGASMNPAVSFGPAVVSWIWTNHWVYWVGPFIGAAIAAIVYDTIFIGSNGHEPLPSNDF

>At TIP2;1 AT3G16240

MAGVAFGSFDDSFSLASLRAYLAEFISTLLFVFAGVGSAIAYAKLTSDAALDTPGLVAIAVCHGFALFVAVAIGANISGGHVNPAVTFGLAVGGQITVITGVFYWIAQLLGSTAACFLLKYVTGGLAVPTHSVAAGLGSIEGVVMEIIITFALVYTVYATAADPKKGSLGTIAPLAIGLIVGANILAAGPFSGGSMNPARSFGPAVAAGDFSGHWVYWVGPLIGGGLAGLIYGNVFMGSSEHVPLASADF

>At TIP2;2 AT4G17340

MVKIEIGSVGDSFSVASLKAYLSEFIATLLFVFAGVGSALAFAKLTSDAALDPAGLVAVAVAHAFALFVGVSIAANISGGHLNPAVTLGLAVGGNITVITGFFYWIAQCLGSIVACLLLVFVTNGESVPTHGVAAGLGAIEGVVMEIVVTFALVYTVYATAADPKKGSLGTIAPIAIGFIVGANILAAGPFSGGSMNPARSFGPAVVSGDFSQIWIYWVGPLVGGALAGLIYGDVFIGSYAPAPTTESYP

>At TIP2;3 AT5G47450

MVKIEVGSVGDSFSVSSLKAYLSEFIATLLFVFAGVGSAVAFAKLTSDGALDPAGLVAIAIAHAFALFVGVSIAANISGGHLNPAVTLGLAIGGNITLITGFFYWIAQCLGSIVACLLLVFVTNGKSVPTHGVSAGLGAVEGVVMEIVVTFALVYTVYATAADPKKGSLGTIAPIAIGFIVGANILAAGPFSGGSMNPARSFGPAVVSGDLSQIWIYWVGPLVGGALAGLIYGDVFIGSYEAVETREIRV

>At TIP3;1 AT1G73190

MATSARRAYGFGRADEATHPDSIRATLAEFLSTFVFVFAAEGSILSLDKLYWEHAAHAGTNTPGGLILVALAHAFALFAAVSAAINVSGGHVNPAVTFGALVGGRVTAIRAIYYWIAQLLGAILACLLLRLTTNGMRPVGFRLASGVGAVNGLVLEIILTFGLVYVVYSTLIDPKRGSLGIIAPLAIGLIVGANILVGGPFSGASMNPARAFGPALVGWRWHDHWIYWVGPFIGSALAALIYEYMVIPTEPPTHHAHGVHQPLAPEDY

>At TIP3;2 AT1G17810

MATSARRAYGFGRADEATHPDSIRATLAEFLSTFVFVFAGEGSILALDKLYWDTAAHTGTNTPGGLVLVALAHALALFAAVSAAINVSGGHVNPAVTFAALIGGRISVIRAIYYWVAQLIGAILACLLLRLATNGLRPVGFHVASGVSELHGLLMEIILTFALVYVVYSTAIDPKRGSIGIIAPLAIGLIVGANILVGGPFDGASMNPARAFGPALVGWRWSNHWIYWVGPFIGGALAALIYEYMIIPSVNEPPHHSTHQPLAPEDY

>At TIP4;1 AT2G25810

MKKIELGHHSEAAKPDCIKALIVEFITTFLFVFAGVGSAMATDSLVGNTLVGLFAVAVAHAFVVAVMISAGHISGGHLNPAVTLGLLLGGHISVFRAFLYWIDQLLASSAACFLLSYLTGGMGTPVHTLASGVSYTQGIIWEIILTFSLLFTVYATIVDPKKGSLDGFGPLLTGFVVGANILAGGAFSGASMNPARSFGPALVSGNWTDHWVYWVGPLIGGGLAGFIYENVLIDRPHVPVADDEQPLLN

>At TIP5;1 AT3G47440

MRRMIPTSFSSKFQGVLSMNALRCYVSEFISTFFFVLAAVGSVMSSRKLMAGDVSGPFGVLIPAIANALALSSSVYISWNVSGGHVNPAVTFAMAVAGRISVPTAMFYWTSQMIASVMACLVLKVTVMEQHVPIYKIAGEMTGFGASVLEGVLAFVLVYTVFTASDPRRGLPLAVGPIFIGFVAGANVLAAGPFSGGSMNPACAFGSAMVYGSFKNQAVYWVGPLLGGATAALVYDNVVVPVEDDRGSSTGDAIGV

>At NIP1;1 AT4g19030

MADISGNGYGNAREEVVMVNLKDEVEHQQEMEDIHNPRPLKKQDSLLSVSVPFLQKLIAEFLGTYFLVFTGCASVVVNMQNDNVVTLPGIAIVWGLTIMVLIYSLGHISGAHINPAVTIAFASCGRFPLKQVPAYVISQVIGSTLAAATLRLLFGLDHDVCSGKHDVFIGSSPVGSDLQAFTMEFIVTFYLMFIISGVATDNRAKLNIGTKCCNIQIGELAGLAIGSTVLLNVLIAAPVSSASMNPGRSLGPALVYGCYKGIWIYLVAPTLGAIAGAWVYNTVRYTDKPLREITKSGSFLKTVRIGST

>At NIP1;2 AT4G18910

MAEISGNGGDARDGAVVVNLKEEDEQQQQQQAIHKPLKKQDSLLSISVPFLQKLMAEVLGTYFLIFAGCAAVAVNTQHDKAVTLPGIAIVWGLTVMVLVYSLGHISGAHFNPAVTIAFASCGRFPLKQVPAYVISQVIGSTLAAATLRLLFGLDQDVCSGKHDVFVGTLPSGSNLQSFVIEFIITFYLMFVISGVATDNRAIGELAGLAVGSTVLLNVIIAGPVSGASMNPGRSLGPAMVYSCYRGLWIYIVSPIVGAVSGAWVYNMVRYTDKPLREITKSGSFLKTVRNGSSR

>At NIP2;1 AT2G34390

MDDISVSKSNHGNVVVLNIKASSLADTSLPSNKHESSSPPLLSVHFLQKLLAELVGTYYLIFAGCAAIAVNAQHNHVVTLVGIAVVWGIVIMVLVYCLGHLSAHFNPAVTLALASSQRFPLNQVPAYITVQVIGSTLASATLRLLFDLNNDVCSKKHDVFLGSSPSGSDLQAFVMEFIITGFLMLVVCAVTTTKRTTEELEGLIIGATVTLNVIFAGEVSGASMNPARSIGPALVWGCYKGIWIYLLAPTLGAVSGALIHKMLPSIQNAEPEFSKTGSSHKRVTDLPL

>At NIP2;2 AT2G29870

MMCAARNTMSSSGSSPSGSDLQAFVMEFIITGFLMLVVCAVTTTKRTTEELEGLIIGATVTLNVIFVGEVSGASMNPARSIGPALVWGCYKGIWIYLLAPTLGAVSRALIHKMLPSIPNAEPKFSKTGSSHKRVSDLPL*

>AtNIP4;2 AT5G37820

MTSHGEEIEDEQISRIEKGNCKDSQGGMETAICSSPSIVCLTQKLIAEMIGTYFIIFSGCGVVVVNVLYGGTITFPGICVTWGLIVMVMIYSTGHISGAHFNPAVTVTFAVFRRFPWYQVPLYIGAQLTGSLLASLTLRLMFNVTPKAFFGTTPTDSSGQALVAEIIISFLLMFVISGVATDSRATGELAGIAVGMTIILNVFVAGPISGASMNPARSLGPAIVMGRYKGIWVYIVGPFVGIFAGGFVYNFMRFTDKPLRELTKSASFLRSVAQKDNASKSDG

>At NIP5;1 AT4G10380

MAPPEAEVGAVMVMAPPTPGTPGTPGGPLITGMRVDSMSFDHRKPTPRCKCLPVMGSTWGQHDTCFTDFPSPDVSLTRKLGAEFVGTFILIFTATAGPIVNQKYDGAETLIGNAACAGLAVMIIILSTGHISGAHLNPSLTIAFAALRHFPWAHVPAYIAAQVSASICASFALKGVFHPFMSGGVTIPSVSLGQAFALEFIITFILLFVVTAVATDTRAVGELAGIAVGATVMLNILVAGPSTGGSMNPVRTLGPAVASGNYRSLWVYLVAPTLGAISGAAVYTGVKLNDSVTDPPRPVRSFRR

>At NIP6;1 AT1G80760

MDHEEIPSTPSTPATTPGTPGAPLFGGFEGKRNGHNGRYTPKSLLKSCKCFSVDNEWALEDGRLPPVTCSLPPPNVSLYRKLGAEFVGTLILIFAGTATAIVNQKTDGAETLIGCAASAGLAVMIVILSTGHISGAHLNPAVTIAFAALKHFPWKHVPVYIGAQVMASVSAAFALKAVFEPTMSGGVTVPTVGLSQAFALEFIISFNLMFVVTAVATDTRAVGELAGIAVGATVMLNILIAGPATSASMNPVRTLGPAIAANNYRAIWVYLTAPILGALIGAGTYTIVKLPEEDEAPKERRSFRR

>At SIP1;1 AT3G04090

MMGVLKSAIGDMLMTFSWVVLSATFGIQTAAIISAGDFQAITWAPLVILTSLIFVYVSIFTVIFGSASFNPTGSAAFYVAGVPGDTLFSLAIRLPAQAIGAAGGALAIMEFIPEKYKHMIGGPSLQVDVHTGAIAETILSFGITFAVLLIILRGPRRLLAKTFLLALATISFVVAGSKYTGPAMNPAIAFGWAYMYSSHNTWDHIYVYWISSFVGALSAALLFRSIFPPPRPQKKKQKKA

>At SIP1;2 AT5G18290

MSAVKSALGDMVITFLWVILSATFGIQTAAIVSAVGFHGITWAPLVISTLVVFVSISIFTVIGNVLGGASFNPCGNAAFYTAGVSSDSLFSLAIRSPAQAIGAAGGAITIMEMIPEKYKTRIGGKPSLQFGAHNGAISEVVLSFSVTFLVLLIILRGPRKLLAKTFLLALATVSVFVVGSKFTRPFMNPAIAFGWAYIYKSHNTWDHFYVYWISSYTGAILSAMLFRIIFPAPPLVQKKQKKA

>At SIP2;1 AT3G56950

MGRIGLVVTDLVLSFMWIWAGVLVNILVHGVLGFSRTDPSGEIVRYLFSIISMFIFAYLQQATKGGLYNPLTALAAGVSGGFSSFIFSVFVRIPVEVIGSILAVKHIIHVFPEIGKGPKLNVAIHHGALTEGILTFFIVLLSMGLTRKIPGSFFMKTWIGSLAKLTLHILGSDLTGGCMNPAAVMGWAYARGEHITKEHLLVYWLGPVKATLLAVWFFKVVFKPLTEEQEKPKAKSE

>PtPIP1;1 POPTRDRAFT_831918

MEEGEEDVKVGANRYGEGQPIGTAAQTQHGKDYTEPPPAPLYQPGEWLSWSFYRAGIAEFVATFLFLYITVLTVMGVARSSTKCSTVGIQGIAWAFGGMIFVLVYCTAGISGGHINPAVTFGLLLARKLTLTRAVFYMIMQCLGAICGAGVVKGFQKSPYEILGGGANTVSTGYSKGSGLGVEILGTFVLVYTVFSATDAKRSARDSHVPVLAPLPIGFAVFLVHLATIPITGTGINPARSLGAALIYNKDKAWDDHWIFWVGPFIGAALASLYHQIVIRAIPFKSK*

>PtPIP1;2 POPTRDRAFT_835561

MEGREEDVRVGANKYGERQPIGTAAQAQDVKDYTDPPPAPLFEPGELSSWSFYRAGIAEFVATFLFLYITVLTVMGVAKSPTKCSTVGIQGIAWAFGGMIFALVYCTAGISGGHINPAVTFGLLLARKLSLTRAVFYMLMQCLGAICGAAVVKAFQKSQYEMLGGGANTVSTGYAKGSGLGAEIVGTFVLVYTVFSATDAKRNARDSHVPILAPLPIGFAVFLVHLATIPITGTGINPARSLGAALIYNKDQAWDDHWIFWVGPFIGAALASLYHQIVIRAIPFKSK*

>PtPIP1;3 POPTRDRAFT_711735

MEGKEEDVKLGANKFSERQPIGTSAQTDKDYKEAPPAPLFEPGELKSWSFYRAGIAEFIATFLFLYITVLTVMGVTKPGTSKCSTVGIQGIAWAFGGMIFALVYCTAGISGGHINPAVTFGLFLARKLSLTRAVFYIIMQCLGAICGAGVVKGLQGSHNYELQGGGANVVNHGYTKGDGLGAEIVGTFVLVYTVFSATDAKRNARDSHVPILAPLPIGFAVFLVHLATIPITGTGINPARSLGAAIIFNKDHAWDDHWIFWVGPFIGAALAAVYHQIVIRAIPFKSRA*

>PtPIP1;4 POPTRDRAFT_724520

MEGKEEDVRLGANKFNERQPLGTAAQSQDDKDYKEPPPAPLFEPSELTSWSFYRAGIAEFMATFLFLYITVLTVMGVFKDTTKCTTVGIQGIAWAFGGMIFALVYCTAGISGGHINPAVTFGLFLARKLSLTRAVFYMLMQCLGAICGAGVVKGFYGKKNYELLNGGANMVSPGYTKGDGLGAEIVGTFVLVYTVFSATDAKRSARDSHVPILAPLPIGFAVFLVHLATIPITGTGINPARSLGAAIIFNKDKAWDDHWIFWVGPFIGAALAALYHQVVIRAIPFKK*

>PtPIP1;5 POPTRDRAFT_656216

MEGKEEDVRLGANRFNERQPIGTAAQSLDDKDYKEPPPAPLFEPGELTSWSFYRAGIAEFMATFLFLYITVLTVMGVVKDQTKCTTVGIQGIAWAFGGMIFALVYCTAGISGGHINPAVTFGLFLARKLSLTRAVFYMVMQCLGAICGAGVVKGFYGKTNYELHNGGANMVAHGYTKGDGLGAEIVGTFILVYTVFSATDAKRSARDSHVPILAPLPIGFAVFLVHLATIPITGTGINPARSLGAAIIFNKDSAWDDHWIFWVGPFIGAALAALYHQVVIRAIPFKK*

>PtPIP2;8 POPTRDRAFT_836572

MSTGGKDYRDPPPAPLLDMEELKQWSFYRALIAEFVATFLFLYIGVGTVVGYKGVHNNLCDGAGYLGVAWAFGGMIFVLVYCTAGISGGHINPAVTFGLFVARKVSLIRAVAYMMAQCLGAMLGVWMVMILTGIHYDQAGGAVNVVAPGYSKGTALGAEIIGTFVLVYTVLAATDPKRMARDSHVPVLAPLPIGFAVFVVHLALIPITGTGINPARSLGAAVVKNAKEIWDDHWIFWVGPFVGALAAAVYHQYILGSGAAKALASFRSNPTS*

>PtPIP2;9 POPTRDRAFT_796664

MSSEERNIERQHGRDYHDPPPAPLLDMGELKQWSFYRAAIAEFIATFLFLFFSVSTVVNYKEPNYTDQCSRVGHLGIAWANGGMIFVLVYCTSGISGGHLNPAVTFGMLVARKMSLIRAAAYMLAQCLGAILGHLFVFLFMYADEQQSSVGVVNVVSRNYSKGAGLGAEFIGTFVLVYTVFSATDPKRNARDSHVPVLAPLPIGFAVFVVHLATIPITGTGINPARSLATNLLHRSTAEAMDDLWIFWVGPFLGALAAAVYHKYVLRAGAVKTLKSFRALGSFGSQPPV*

>PtTIP1;1 POPTRDRAFT_667870

MPIRNIAVGHYRETTQPDALKAALAEFISTLIFVFAGEGSGMAFSKLTDGASNTPAGLIAAAIAHAFALFVAVSVGANISGGHVNPAVTFGAFIGGNITLFRGILYWIAQLLGSTVACLLLKFVTGGLETSAFALSTGVGVWNAFVLEIVMTFGLVYTVYATAIDPKKGNLGIIAPIAIGFIVGANILVGGAFDGASMNPAVSFGPALVSWSWTNHWVYWAGPLVGGGLAGLIYELFFIGFGTHEQLPTTDY*

>PtTIP1;2 POPTRDRAFT_589502

MPIRNIAVGHYHEATQPDALRAALAEFISTLIFVFAGEGSGMAFAKLTDGAANTPAGLIAAAIAHAFALFVAVSVGANISGGHVNPAVTFGAFIGGNITLLRGILYWIAQLLGSTVACLLLKFTTGGLETSAFALSSGVGVWNAFVLEIVMTFGLVYTVYATAVDPKKGNLGIIAPIAIGFIVGANILAGGAFDGASMNPAVSFGPALVSWTWTNHWVYWAGPLIGGGLAGLIYEFFFIGFGNHEQLPTADY*

>PtTIP1;3 POPTRDRAFT_828458

MRNFIIIERITIGRVEDDFHSNAFKAALAEFISTLIFVFAGQGSTMAYNKLTSNAPTSPAGLIAVALAHAFGLFVGVAVSANISGGHVNPAVTFGAFIGGNISLLRGILYWIAQLLGSTVACLLLKYTTHHMTVSVFTLSPGVTVWNAFVFEIVMTFALVYTVYATAIDPKKGDVGVIAPLAIGFVLGANILVGGAFEGAALNPAVPFGPALVSWNWYHHWVYWAGPLIGGGLAGIVYELIFMSHSTHEPLPGGEF*

>PtTIP1;4 POPTRDRAFT_558321

MPNLIVIDRIAIGTVAADFHPNAFKAALAEFISTLIFVFAGQGSTMAYNKLTSNAPTSPAGLIAVALAHAFGLFVAVATSANISGGHCNPAVTFGAFLGGNITLLRGILYWIAQLLGSTVACLLLKFTTHYMTVSVFTLSPGVSVWNAFVFEIVMTFALVYTVYATAIDAKKGDVGVIAPLAIGFVLGANILAGGAFEGAALNPAVPFGPALVSWNWHHHWVYWAGPLIGGGLAGVVYELIFISHTHEPLAVVEY*

>PtTIP2;1 POPTRDRAFT_676397

MVKIAFGSLGDSFSVGSLKAYLSEFIATLLFVFAGVGSAIAYSKLTTDAALDPPGLVAVAVAHAFALFVGVSIAANISGGHLNPAVTFGLAIGGNITILTGLLYWIAQCLGSIAACLLLKFATSAESIPTHGVASGMSAVEGVVMEIVITFALVYTVYATAADPKKGSIGIIAPIAIGFIVGANILAAGPFSGGSMNPARSFGPAVVSGDFSQNWIYWLGPLIGGGLAGLVYGDIFIGSYTAAPVSEDYA*

>PtTIP2;2 POPTRDRAFT_817166

MAKIAFGSLGDSFSLASLKAYLSEFIATLLFVFAGVGSAIAYSKLTTDAALDPPGLVAVAVAHAFALFVGVSIAANISGGHLNPAVTFGLAIGGNITFLTGLLYWIAQCLGSIVACLLLKVVTSAEGIPTHGVASGMSAIEGVVMEIVITFALVYTVYATAADPKKGSLGIIAPIAIGFIVGANILAAGPFSGGSMNPARSFGPAVVSGDFSQNWIYWLGPLVGGGLAGLVYGGIFIGSYAPAPVSEDYA*

>PtTIP2;3 POPTRDRAFT_548890

MAGIAFGRFDDSFSLGSFKAYLAEFISTLLFVFAGVGSAMAYNKLTGDAALDPAGLVAIAVCHGFALFVAVSVGANISGGHVNPAVTFGLALGGQITILTGIFYWIAQLLGSIVACYLLKVATGGLAVPIHSVAAGVGAIEGVVMEIIITFALVYTVYATAADPKKGSLGTIAPIAIGFIVGANILAAGPFSGGSMNPARSFGPAVASGDFHDNWIYWAGPLVGGGIAGLIYGNVFITDHTPLSGDF*

>PtTIP2;4 POPTRDRAFT_645978

MARIAFGRFNDSFSLGSLKAYLAEFISTLLFVFAGVGSAMAYNKLTGDAALDPAGLVAIAVCHGFALFVAVAVGANISGGHVNPAVTLGLALGGQMTILTGIFYWIAQLLGSIVACYLLKVVTGGLAVPIHSVAAGVGAIEGVVMEIIITFALVYTVYATAADPKKGSLGTIAPIAIGFIVGANILAAGPFSGGSMNPARSFGPAVASGDFHDNWIYWVGPLIGGGLAGLIYGNLYITDHSPSSYEF*

>Pb_Contig9700

APKGKSSMAPMGIEGGEQESRDYEDHSPAPLLDSLELKRWSFYRATMAEFVATLLFLYITLTTVVENKRSKGTCGGVGLLGEASAFGGMISVLVYCISAISGGHVNPAATFALFLARKISLPRALLYIVAQCLGALCGTALVRGMQGSFYASTGGGSNSVSTGYSKGTALLAEIIGTFVLVYTVFSAADPKRKAHHSHIPVLAPIPIGLAVFLVHLATIPITGTGINPARSFGPAVIYGHQKSWDDLWIFWVGPLIGAAVAAAYHQYILRAGRFGQKNSASLKSHPTSAI*

>Pb_Contig8970

IAYIIAVDCRSRIGAEIFRAKRDLILRVHNMGKIAVGRVEEATQADSLRAIVAELVCTFLFVFAGVGSTMAVDSLSESSGLTPGGGLAIIALTHAFAVCGLVSAGFHLSGGHINPAVTFALAVGGHITILRSILYWIAQLLGSTIACYLLYFITGGMGTPVHTLAGGMGYIQGVVMEMVLTFSLLFTVYATVVDPRRGPMGVIMAPLCIGLVVGANVMAAGPFSSASMNPARSFGPAFVMWQWRDHWVYWVGPLVGGGLAAALYENFFIIRTYEPLPASL*

>Pb_Contig560

IKSSVGRETERAESFLRFVFSMAKEGGKDMQEQQQGFVAKDYKDPPPAALVDINEFKLWSFYRALIAEFIATLLFLYITIATVIGHSRTKADCGSVGVLGIAWSFGGMIFVLVYCTAGISGGHINPAVTFGLFLARKVSLPRAVLYMIAQCLGAICGAGLVKAFQKPYYDRYGGGANVVAHGYTKGVGLAAEIIGTFVLVYTVFSATDPKRSARDSHVPVLAPLPIGFAVFMVHLATIPITGTGINPARSFGAAVIYGHKHSWDDQWIFWVGPMVGAAAAAAYHQHILRATAIKALGSFRSNPQV*

>Pb_Contig552

EKGSSYQPPTHLHAWHHKFGILLQLQIHLSPRENMGIVELAIGDAAITFLWVFGASCLGAGTSVIASYLGVQGAMTLLITLSLLFLLVFLFSFLGEAMGGATWNPTALAAAFALGVGKDNLISLSIRFPAQAAGAVGGALAIMELMPASHKHMLGGPSLKVDLHKGAIAEGVLTFVISIMVFLIIMKGPKSSFWKSWLISLTTIILVLAGSGYTGPSMNPANAFGWAYVNNRHNTWEQLYVYWITPFIGSILAAWTLRLISPPNSSKKEKKA*

>Pb_Contig505

LVTRQLTEMAKIALGNRDEPARPDCVRAVFVELICTFLFVFAGVGSAMAMDQMSVPANSPAGLTVVALTHAFVVFAMISAGFSISGGHLNPAVTLGLAVGGHISLIRSLLYWIAQLLASALACFLLKFLTGGSATPVHTLSSGMTYFQGVIMEIVLTFSLLFTVYATAVDPKKGNVGITAPLCVGLVVGANIFAGGPFSGASMNPARSFGPALVTGIWKDHWVYWVGPLVGGGLAGFVYENIFIYETHTPLPDVEY*

>Pb_Contig4499

GDHTFIHSAFAALHYFGFISTKFRQSLNPLVLSVEMEGKEEDVKLGANKYSERQPLGTAAQTREKDYKEPGPAPLFEPGELASWSFWRAGIAEFMATFLFLYITILTVMGVKRADNVCTGSVGIQGIAWAFGGMIFCLVYCTAGISGGHINPAVTFGLFLARKLSLPRAVFYMICQCLGAICGAGVVKGFMEGEYQADGGGANSVAHGYTKGDGLGAEIVGTFVLVYTVFSATDAKRSARDSHVPLLAPLPIGFAVFLVHLATIPITGTGINPARSLGAAIIYNKSHVWDDHWIFWVGPFLGAGLAAIYHQMIIRAIPFKTRS*

>Pb_Contig4140

VSLTSPAILSFFEFISRSRSSVPAIMTKEERKELEQQGFTSKDYTDPPPAALIETSEFKLWSFYRALIAEFTATLLFLYITIATVIGHSRNSANCGSVGVLGIAWSFGGMIFVLVYCTAGISGGHINPAVTFGLFLARKVSLPRAILYMIAQCLGAICGTGLVKAFQKSFYDRYGGGANYVHHGYTKGVGLAAEIIGTFVLVYTVFSATDPKRSARDSHVPVLAPLPIGFAVFMVHLATIPITGTGINPARSFGAAVIYGRKQP*

>Pb_Contig3965

GAGGMGYIQGVVMEMVLTFSLLFTVYATVVDPRRGPMGVIMAPLCMGLVVGANVMAAGPFSSASMNPARSFGPAFVMWQWRDHWVYWVGPLVGGGLAAALYENFFIVRTYEPLPASL*

>Pb_Contig3789

MAKESGTDIEPPAKDYKDPPPAPFVDVREFTLWSFYRAVIAEFIATLLFLYITIATVIGHSRTKANCGSVGVLGIAWSFGGMIFILVYCTAGISGGHINPAVTFGLFLARKVSFPRAVCYMIAQCLGAICGVGLVKAFQKSYYDRYGGGANVVAHGYTKGVGLAAEIIGTFILVYTVFSATDPKRSARDSHVPVLAPLPIGFAVFIVHLGTIPITGTGINPARSFGAAVIYGHQKAWDDQWIFWVGPFIGAACAAAYHQYIVRAGAIKALGSFRSVPSI*

>Pb_Contig3654

SMEKEARSGKVEYEEPPSAPLLDTNEFYLWSFYRAIIAEFVATLLFLYVTIATVIGNANDKSPCGGVGTLGIAWSFGGMIFVLVYCTAGISGGHLNPAVTFGLFVARKVTLNRAALYMVAQCLGAICGVGMVKALQKTYYSSGGGGANSVSKGYGNETALTAEIVGTFVLVYTVFYATDIKSNAHDAHVPALAPLSIGFAVFMVHLATIPVTGTGINPARSFGAAVIYGHKKSWDDHWIFWVGPLIGATVAAAFHKYVIRASLKWHSKEPNHV*

>Pb_Contig3651

VSLTSPAILSFFEFISRSRSSVPAIMTKEERKELEQQGFPSKDYTDPPPAALIETSEFKLWSFYRALIAEFTATLLFLYITIATVIGHSRNSTNCGSVGVLGIAWSFGGMIFVLVYCTAGISGGHINPAVTFGLFLARKVSLPRAILYMIAQCLGAICGTGLVKAFQKSFYDTYGGGANYVHHGYTKGVGLAAEIIGTFVLVYTVFSATDPKRSARDSHVPVLAPLPIGFAVFMVHLATIPITGTGINPARSFGAAVIYGRKQPWNDQWIFWVGPFVGAALASAYHQYILRAAAIKALGSFRSNANV*

>Pb_Contig1949

ALVQGPIMALDNMPEQVNANSVRNIEEGRVESYVYTERTCGSFLPSVVFAQKVVAEIIGTFFLIFIGCGAVVIDKKTNGSITHLGVSLVWGLAVMIIIYSTGHVSGAHLNPAVTLAFAAVRRFPWTQVPAYIGAQVFAAICAGFVLRLMFGDVAHIGATVPSGSDMQSFVLEIFVTYLLMFVISAVATDTRAIGELAGMAVGATIAMNVAISGPISGASMNPARTLGSAVAGNKYTSIWIYMVAPILGAILGALSYNMIRLTDKPVREITQTGSFLKSQGPSRS*

>Pb_Contig1070

SMEKEARSGKVEYEEPPSAPLLDTNEFYLWLFYRAIIAEFVATLLFLYVTIATVIGNANDKSPCGGVGTLGIAWSFGGMIFVLVYCTAGISGGHLNPAVTFGLFVARKVTLNRAVLYMVAQCLGAICGVGMVKALQKTYYSSGGGGANSVSKGYGNETALTAEIVGTFVLVYTVFYATDIKSNARDAHVPALAPLSIGFAVFMVHLATIPVTGTGINPARSFGAAVIYGHKKSWDDHWIFWVGPLIGATIAAAFHKYVIRASLKWHSKEPNHV*

>AtCBF-DREB1A AT4G25480

MNSFSAFSEMFGSDYESSVSSGGDYIPTLASSCPKKPAGRKKFRETRHPIYRGVRRRNSGKWVCEVREPNKKTRIWLGTFQTAEMAARAHDVAALALRGRSACLNFADSAWRLRIPESTCAKDIQKAAAEAALAFQDEMCDATTDHGFDMEETLVEAIYTAEQSENAFYMHDEAMFEMPSLLANMAEGMLLPLPSVQWNHNHEVDGDDDDVSLWSY

>AtCBF-DREB2 gi|4091984

MNSCSAFSEMFGSDYESPVSSGGDYSPKLATSCPKKPAGRKKFRETRHPIYRGVRQRNSGKWVCELREPNKKTRIWLGTFQTAEMAARAHDVAAIALRGRSACLNFADSAWRLRIPESTCAKEIQKAAAEAALNFQDEMCHMTTDAHGLDMEETLVEAIYTPEQSQDAFYMDEEAMLGMSSLLDNMAEGMLLPSPSVQWNYNFDVEGDDDVSLWSY

>AtDREB2A AT5G05410

MAVYDQSGDRNRTQIDTSRKRKSRSRGDGTTVAERLKRWKEYNETVEEVSTKKRKVPAKGSKKGCMKGKGGPENSRCSFRGVRQRIWGKWVAEIREPNRGSRLWLGTFPTAQEAASAYDEAAKAMYGPLARLNFPRSDASEVTSTSSQSEVCTVETPGCVHVKTEDPDCESKPFSGGVEPMYCLENGAEEMKRGVKADKHWLSEFEHNYWSDILKEKEKQKEQGIVETCQQQQQDSLSVADYGWPNDVDQSHLDSSDMFDVDELLRDLNGDDVFAGLNQDRYPGNSVANGSYRPESQQSGFDPLQSLNYGIPPFQLEGKDGNGFFDDLSYLDLEN

>AtDREB2B AT3G11020

MAVYEQTGTEQPKKRKSRARAGGLTVADRLKKWKEYNEIVEASAVKEGEKPKRKVPAKGSKKGCMKGKGGPDNSHCSFRGVRQRIWGKWVAEIREPKIGTRLWLGTFPTAEKAASAYDEAATAMYGSLARLNFPQSVGSEFTSTSSQSEVCTVENKAVVCGDVCVKHEDTDCESNPFSQILDVREESCGTRPDSCTVGHQDMNSSLNYDLLLEFEQQYWGQVLQEKEKPKQEEEEIQQQQQEQQQQQLQPDLLTVADYGWPWSNDIVNDQTSWDPNECFDINELLGDLNEPGPHQSQDQNHVNSGSYDLHPLHLEPHDGHEFNGLSSLDI

>AtRAP2.4 AT1G78080

MAAAMNLYTCSRSFQDSGGELMDALVPFIKSVSDSPSSSSAASASAFLHPSAFSLPPLPGYYPDSTFLTQPFSYGSDLQQTGSLIGLNNLSSSQIHQIQSQIHHPLPPTHHNNNNSFSNLLSPKPLLMKQSGVAGSCFAYGSGVPSKPTKLYRGVRQRHWGKWVAEIRLPRNRTRLWLGTFDTAEEAALAYDKAAYKLRGDFARLNFPNLRHNGSHIGGDFGEYKPLHSSVDAKLEAICKSMAETQKQDKSTKSSKKREKKVSSPDLSEKVKAEENSVSIGGSPPVTEFEESTAGSSPLSDLTFADPEEPPQWNETFSLEKYPSYEIDWDSILA

>AtTINY AT5G25810

MIASESTKSWEASAVRQENEEEKKKPVKDSGKHPVYRGVRKRNWGKWVSEIREPRKKSRIWLGTFPSPEMAARAHDVAALSIKGASAILNFPDLAGSFPRPSSLSPRDIQVAALKAAHMETSQSFSSSSSLTFSSSQSSSSLESLVSSSATGSEELGEIVELPSLGSSYDGLTQLGNEFIFSDSADLWPYPPQWSEGDYQMIPASLSQDWDLQGLYNY

>AtDREB2 AT2G38340

MEKEDNGSKQSSSASVVSSRRRRRVVEPVEATLQRWEEEGLARARRVQAKGSKKGCMRGKGGPENPVCRFRGVRQRVWGKWVAEIREPVSHRGANSSRSKRLWLGTFATAAEAALAYDRAASVMYGPYARLNFPEDLGGGRKKDEEAESSGGYWLETNKAGNGVIETEGGKDYVVYNEDAIELGHDKTQNPMTDNEIVNPAVKSEEGYSYDRFKLDNGLLYNEPQSSSYHQGGGFDSYFEYFRF

>AtDREB2C-ERF48 AT2G40340

MPSEIVDRKRKSRGTRDVAEILRQWREYNEQIEAESCIDGGGPKSIRKPPPKGSRKGCMKGKGGPENGICDYRGVRQRRWGKWVAEIREPDGGARLWLGTFSSSYEAALAYDEAAKAIYGQSARLNLPEITNRSSSTAATATVSGSVTAFSDESEVCAREDTNASSGFGQVKLEDCSDEYVLLDSSQCIKEELKGKEEVREEHNLAVGFGIGQDSKRETLDAWLMGNGNEQEPLEFGVDETFDINELLGILNDNNVSGQETMQYQVDRHPNFSYQTQFPNSNLLGSLNPMEIAQPGVDYGCPYVQPSDMENYGIDLDHRRFNDLDIQDLDFGGDKDVHGST

>AtDREB2H-ERF47 AT2G40350

MPRKRKSRGTRDVAEILRKWREYNEQTEADSCIDGGGSKPIRKAPPKRSRKGCMKGKGGPENGICDYTGVRQRTWGKWVAEIREPGRGAKLWLGTFSSSYEAALAYDEASKAIYGQSARLNLPLLPLCQARLLHFLMNLKFVHVRIQMQDLVLVRSD

>AtDREB2F-ERF51 AT3G57600

MEKSSSMKQWKKGPARGKGGPQNALCQYRGVRQRTWGKWVAEIREPKKRARLWLGSFATAEEAAMAYDEAALKLYGHDAYLNLPHLQRNTRPSLSNSQRFKWVPSRKFISMFPSCGMLNVNAQPSVHIIQQRLEELKKTGLLSQSYSSSSSSTESKTNTSFLDEKTSKGETDNMFEGGDQKKPEIDLTEFLQQLGILKDENEAEPSEVAECHSPPPWNEQEETGSPFRTENFSWDTLIEMPRSETTTMQFDSSNFGSYDFEDDVSFPSIWDYYGSLD

>AtDREB2G-ERF50 AT5G18450

MEEEQPPAKKRNMGRSRKGCMKGKGGPENATCTFRGVRQRTWGKWVAEIREPNRGTRLWLGTFNTSVEAAMAYDEAAKKLYGHEAKLNLVHPQQQQQVVVNRNLSFSGHGSGSWAYNKKLDMVHGLDLGLGQASCSRGSCSERSSFLQEDDDHSHNRCSSSSGSNLCWLLPKQSDSQDQETVNATTSYGGEGGGGSTLTFSTNLKPKNLMSQNYGLYNGAWSRFLVGQEKKTEHDVSSSCGSSDNKESMLVPSCGGERMHRPELEERTGYLEMDDLLEIDDLGLLIGKNGDFKNWCCEEFQHPWNWF

>AtCBF2-DREB1C gi|4091983

MNSCSAFSEMFGSDYESPVSSGGDYSPKLATSCPKKPAGRKKFRETRHPIYRGVRQRNSGKWVCELREPNKKTRIWLGTFQTAEMAARAHDVAAIALRGRSACLNFADSAWRLRIPESTCAKEIQKAAAEAALNFQDEMCHMTTDAHGLDMEETLVEAIYTPEQSQDAFYMDEEAMLGMSSLLDNMAEGMLLPSPSVQWNYNFDVEGDDDVSLWSY

>AtCBF4-DREB1D AT5G51990

MNPFYSTFPDSFLSISDHRSPVSDSSECSPKLASSCPKKRAGRKKFRETRHPIYRGVRQRNSGKWVCEVREPNKKSRIWLGTFPTVEMAARAHDVAALALRGRSACLNFADSAWRLRIPETTCPKEIQKAASEAAMAFQNETTTEGSKTAAEAEEAAGEGVREGERRAEEQNGGVFYMDDEALLGMPNFFENMAEGMLLPPPEVGWNHNDFDGVGDVSLWSFDE

>AtDDF2 AT1G63030

MENDDITVAEMKPKKRAGRRIFKETRHPIYRGVRRRDGDKWVCEVREPIHQRRVWLGTYPTADMAARAHDVAVLALRGRSACLNFSDSAWRLPVPASTDPDTIRRTAAEAAEMFRPPEFSTGITVLPSASEFDTSDEGVAGMMMRLAEEPLMSPPRSYIDMNTSVYVDEEMCYEDLSLWSY

>AtDDF1 AT1G12610

MNNDDIILAEMRPKKRAGRRVFKETRHPVYRGIRRRNGDKWVCEVREPTHQRRIWLGTYPTADMAARAHDVAVLALRGRSACLNFADSAWRLPVPESNDPDVIRRVAAEAAEMFRPVDLESGITVLPCAGDDVDLGFGSGSGSGSGSEERNSSSYGFGDYEEVSTTMMRLAEGPLMSPPRSYMEDMTPTNVYTEEEMCYEDMSLWSYRY

>At-ERF27 AT1G12630

MNSSMASAGLGSRRKDPVYRGIRCRSGKWVSEIREPRKTTRIWLGTYPMAEMAAAAYDVAAMALKGREAVLNFPGSVGSYPVPESTSAADIRAAAAAAAAMKGCEEGEEEKKAKEKKSSSSKSRARECHVDNDVGSSSWCGTEFMDEEEVLNMPNLLANMAEGMMVAPPSWMGSRPSDDSPENSNDEDLWGY

>At-ERF25 AT5G52020

MSNNNNSPTTVNQETTTSREVSITLPTDQSPQTSPGSSSSPSPRPSGGSPARRTATGLSGKHSIFRGIRLRNGKWVSEIREPRKTTRIWLGTYPVPEMAAAAYDVAALALKGPDAVLNFPGLALTYVAPVSNSAADIRAAASRAAEMKQPDQGGDEKVLEPVQPGKEEELEEVSCNSCSLEFMDEEAMLNMPTLLTEMAEGMLMSPPRMMIHPTMEDDSPENHEGDNLWSYK

>PcTINY-like2

MPKRRAEEVNNADESGKVESAACNGDEEQWVKIKGKRHRRYRGVRMRSWGKWVSEIREPKKKSRIWLGTFSTPEMAARAHDVAALSIKGKSAFLNFPHIASSLPRPATLSPKDIQAAAAVAAAEFHMPSEEDCSEDRSLDPVETNNVILSHADTVIPADSGNNEDAAAVCASNSVWVSSFLSENECMTIYDDILFDLPNIVGNMAEGLLVTPPWMVEQDGYSASDNFYDVNGSISAETSLWNYS

>PcTINY-like1

MRKRRGRKINNAGEPGEVESAVCNGDEKQGVRIKEKRHRVYRGVRMRSWGRWVSEIREPKKKSRIWLGTFPTPEMAARAHDVAALSIKGKSAFLNFPHMASSLPRPATLCPKDIQAAAALAAAEFHMPSEEDCSEDRSLDPVGTSNAIPSDSHTVIPAESGNKESAAVVCASNFVSSFLSGNEWMSIEDDIVLDLPNIVGNMAEGLLVPPPWMLEHDNYSAADNFFDENASISAETSLWNYS

>Pc_Contig5286

MVLSFEPESSPCCGLNDQQGRSRCADCGPLVSINSCICPDLSEGNCIYHSAMIDAASSTSTGSSGSDSALALMQSRSRKRRAAEISNADDSGEEESAVCNGDKQGGRGKGNRHPVYRGVRMRTWGKWVSEIREPKKKSRIWLGTFPTPEMAARAHDVAALSIKGKSAFLNFPNLAASLPRPATLSPKDIQAAAAAAATEFQIPKDLSEDRSLDLAEMSSVSPVHGNALVPAESRNNEQAVATDCAPSNLAWVSSFLSENESMIIDDDILFDLPNFLCNMAEGLLVAPPWLLEQEDLYGSSYYAADNVYEGNGAMSLWN

>AtTINY2 AT5G11590

MAEEYYSLRSERVTQLLVPNSESDSVSDKSKAEQSEKKTKRGRDSGKHPVYRGVRMRNWGKWVSEIREPRKKSRIWLGTFPTPEMAARAHDVAALSIKGTAAILNFPELADSFPRPVSLSPRDIQTAALKAAHMEPTTSFSSSTSSSSSLSSTSSLESLVLVMDLSRTESEELGEIVELPSLGASYDVDSANLGNEFVFYDSVDYCLYPPPWGQSSEDNYGHGISPNFGHGLSWD

>AtERF38 AT2G35700

MERDDCRRFQDSPAQTTERRVKYKPKKKRAKDDDDEKVVSKHPNFRGVRMRQWGKWVSEIREPKKKSRIWLGTFSTAEMAARAHDVAALAIKGGSAHLNFPELAYHLPRPASADPKDIQAAAAAAAAAVAIDMDVETSSPSPSPTVTETSSPAMIALSDDAFSDLPDLLLNVNHNIDGFWDSFPYEEPFLSQSY

>AtERF39 AT4G16750

MQDSSSHESQRNLRSPVPEKTGKSSKTKNEQKGVSKQPNFRGVRMRQWGKWVSEIREPRKKSRIWLGTFSTPEMAARAHDVAALAIKGGSAHLNFPELAYHLPRPASADPKDIQEAAAAAAAVDWKAPESPSSTVTSSPVADDAFSDLPDLLLDVNDHNKNDGFWDSFPYEDPFFLENY

>AtERF34 AT2G44940

MARQINIESSVSQVTFISSAIPAVSSSSSITASASLSSSPTTSSSSSSSTNSNFIEEDNSKRKASRRSLSSLVSVEDDDDQNGGGGKRRKTNGGDKHPTYRGVRMRSWGKWVSEIREPRKKSRIWLGTYPTAEMAARAHDVAALAIKGTTAYLNFPKLAGELPRPVTNSPKDIQAAASLAAVNWQDSVNDVSNSEVAEIVEAEPSRAVVAQLFSSDTSTTTTTQSQEYSEASCASTSACTDKDSEEEKLFDLPDLFTDENEMMIRNDAFCYYSSTWQLCGADAGFRLEEPFFLSE

>AtERF35 AT3G60490

MGKQINIESSATHHQDNIVSVITATISSSSVVTSSSDSWSTSKRSLVQDNDSGGKRRKSNVSDDNKNPTSYRGVRMRSWGKWVSEIREPRKKSRIWLGTYPTAEMAARAHDVAALAIKGNSGFLNFPELSGLLPRPVSCSPKDIQAAATKAAEATTWHKPVIDKKLADELSHSELLSTAQSSTSSSFVFSSDTSETSSTDKESNEETVFDLPDLFTDGLMNPNDAFCLCNGTFTWQLYGEEDVGFRFEEPFNWQND

>AtERF36 AT3G16280

MTSLNSSASPTSSSSDQSDATTTTSTHLSEEEAPPRNNNTRKRRRDSSSASSSSSMQHPVYRGVRMRSWGKWVSEIRQPRKKTRIWLGTFVTADMAARAHDVAALTIKGSSAVLNFPELASLFPRPASSSPHDIQTAAAEAAAMVVEEKLLEKDEAPEAPPSSESSYVAAESEDEERLEKIVELPNIEEGSYDESVTSRADLAYSEPFDCWVYPPVMDFYEEISEFNFVELWSFNH

>AtERF37 AT1G77200

MTESSIISVKQSSPVPEEEDHHHHQQDSHRTNTKKRVRSDPGYRGVRMRTWGKWVSEIREPRKKSRIWLGTFSTPEMAARAHDAAALTIKGTSAVLNFPELATYLPRPASSSPRDVQAAAAVAAAMDFSPSSSSLVVSDPTTVIAPAETQLSSSSYSTCTSSSLSPSSEEAASTAEELSEIVELPSLETSYDESLSEFVYVDSAYPPSSPWYINNCYSFYYHSDENGISMAEPFDSSNFGPLFP

>AtERF42 AT2G25820

MVDSHGSDTECSSKKKKEKTKEKGVYRGARMRSWGKWVSEIREPRKKSRIWLGTFPTAEMAARAHDVAALSIKGSSAILNFPELADFLPRPVSLSQQDIQAAAAEAALMDFKTVPFHLQDDSTPLQTRCDTEKIEKWSSSSSSASSSSSSSSSSSSSMLSGELGDIVELPSLENNVKYDCALYDSLEGLVSMPPWLDATENDFRYGDDSVLLDPCLKESFLWNYE

>AtERF43 AT4G32800

MADSSSDKEKKENNKQPVYRGVRMRSWGKWVSEIREPRKKSRIWLGTFPTAEMAMRAHDVAAMSIKGTSAILNFPELSKLLPRPVSLSPRDVRAAATKAALMDFDTTAFRSDTETSETTTSNKMSESSESNETVSFSSSSWSSVTSIEESTVSDDLDEIVKLPSLGTSLNESNEFVIFDSLEDLVYMPRWLSGTEEEVFTYNNNDSSLNYSSVFESWKHFP

>PcDREB-like

MAEKQFVPNSPNGMVELESKKRKNRACDEAEGAGSYPIYRGVRRRRWGKWVSEIREPRKKKRIWLGSYDTPQMAARAHDVAALCLRGKAACLNFPDLVGMFPRPSSLDPSDIQSAAAEAARAFNGETFSLQSSSAHGSYRDQQNYYNALSESLRESLGPDHAENSVHEQQATGNSASTPCTELELRLNLWWPPPKIIQEKL

>At_ERF23 AT1G01250

MSPQRMKLSSPPVTNNEPTATASAVKSCGGGGKETSSSTTRHPVYHGVRKRRWGKWVSEIREPRKKSRIWLGSFPVPEMAAKAYDVAAFCLKGRKAQLNFPEEIEDLPRPSTCTPRDIQVAAAKAANAVKIIKMGDDDVAGIDDGDDFWEGIELPELMMSGGGWSPEPFVAGDDATWLVDGDLYQYQFMACL

>Pc_Contig4380

MDKEASSIPETMVTLATSSGASSKSASTGKGGVKRIPTQNRAASSSPQERTLKLKIPTYKGVRRRSWGKWVSEIKEPKKKSKIWLGSFDTPEMAARAYDVAEFYLKGKKQALLNFPEMIDHLLQPLSLSPRDIQVAAEEAAVAFYFSEQERKSPRNPPVSNEPPSSHQLLTVISSGFEATDVELPGEADTSNYSSSVCTPTEGVGESESTVVEDDSFKSFHLFTNWAEAPLLSPRPLFMVHEEEHNLEEGFLWSGF

>At_ERF22 AT1G33760

MENTYVGQRDYRFNVNQLSYRGIRRRKWGKWVSEIREPGKKTRIWLGSYETAEMAAAAYDAAALHLRGRGTNLNFPELVDSFPRPESSSSEHIQAAAQDAALMFKPGRLSEPALESGQGLSRVGLSPDQIQAINESPLDSPRMGWMQDLEVADYEELYGQFFGQHDRDEFFEMQQFQSIWNSNN

>At_ERF21 AT1G71450

MAGLRNSGNSDKAQNDGKGVPSAYRGVRKRKWGKWVSEIREPGTKNRIWLGSFETPEMAATAYDVAAFHFRGREARLNFPELASSLPRPADSSSDSIRMAVHEATLCRTTEGTESAMQVDSSSSSNVAPTMVRLSPREIQAINESTLGSPTTMMHSTYDPMEFANDVEMNAWETYQSDFLWDP

>Pc_Contig8996

MVKPSPKQNANGKPENRSLMSRQFKGIRLRKWGKWVSEIRMPNCRAKIWLGSYDTPEKAARAYDFAAYCLRGSKARFNFPDSPPEMPCASSLSPPQIQAAAARFAAEEFRLPSDDDTASSSCGSVAESDLLPGIPCASSVSPPPIQAAAARFAAEEFRLPSDEDTASSSCGSVAESEIDSQQILAEQGSAFWDSLLLEDLYNGESLNLEDFPPIDLAMDDLGFLLQPTEDWLIF

>Pc_Contig2345

MVKPMPKQSSPSGSENCQIKSRQFKGIRLRKWGKWVSEIRMPNSRAKIWLGSYDSPEKAARAYDFALYCLRGSKATFNFPDSPPEIPCASDLSPPQIQAAAARFAIEDFQLPSEEDAASSSSSPSEAESGIDSQQISAEQWPAFWDSLLLEDLDSGESLNLEDFPPLDFSMDDFGFLFEQTKDWGLF

>Pc_Contig2409

GHSSTQSSSLKSGQFKGIRRRKWGKWVSEIRIPNSSGRIWLGSYDTPEKAARAYDFAVYCLRGSNAKLNFPHSPPEILCASSLSPIQIQSAAAKFAAQEFRLPSENSAASSSSSLGVECNMEDQQITAEKDHAFRESVPLEGTDCWGSPNIEDIPPLDVWMEDFEILFQLTEDWGPWISNFYFPAAEILL

>Pc_Contig4057

MVKPLPEQTSPSGPENCQMKSRQFKGIRLRKWGKWVSEIRMPNSRAKIWLGSYDSPEKAARAYDFAVYCLRGSKAKFNFPDSPAEIPCPSCLSPPQPGSPQKISGCRQKRTRQPPLRKQSPALTANRFLRNSAPHSGIRYCLKIWTVASL

>AtERF17 AT1G19210

MEGSSSSMQSKYKGVRKRKWGKWVSEIRLPNSRERIWLGSYDTPEKAARAFDAALYCLRGNNAKFNFPDNPPVISGGRNLSRSEIREAAARFANSAEDDSSGGAGYEIRQESASTSMDVDSEFLSMLPTVGSGNFASEFGLFPGFDDFSDEYSGDRFREQLSPTQDYYQLGEETYADGSMFLWNF

>Pc_Contig4321

MKKEVDPAIVVKRSREAETEELGSKRFRGVRRRSWGKWVAEIRMLRCRSRVWLGSYHTAEQAARAYDAASFCLRGPAAFLNFPESPPAQFLPYPLRPLHDIHLSPQQIRTIAANYATMTPSSTSISTSTSGLQPQQSKPMDTSTLEEAGGEGSASVTTHVDSAKIGPRKMVFPNLNDTLDDEFGDFLER

>Pc_Contig8985

MVCTSKEPEPHQNVLIGSQRHPQRREFQVSSKASKKSSRGSSSRRYRGVRMRSWGSWVSEIRAPHDKKRIWLGSYSSAEAAARAYDAALLCLKGPSATFNFPESATIIHPPQSCQPLLMSPRSIQKVAAAAAAYGSASPATDFAGSVGVDCAAQSLQVSLDSNVKTEIGHQIMPSTANIHSDMGVTGEISWDSPRGVHYTYDQMLSCALLSSDLAPYSDWVDASYAEYRHDDEDEDLCNLWSFP

>AtERF10 AT5G67190

MEGGGVADVAVPGTRKRDRPYKGIRMRKWGKWVAEIREPNKRSRLWLGSYSTPEAAARAYDTAVFYLRGPTARLNFPELLPGEKFSDEDMSAATIRKKATEVGAQVDALGTAVQNNRHRVFGQNRDSDVDNKNFHRNYQNGEREEEEEDEDDKRLRSGGRLLDRVDLNKLPDPESSDEEWESKH

>AtERF19 AT1G22810

MDYRESTGESQSKYKGIRRRKWGKWVSEIRVPGTRDRLWLGSFSTAEGAAVAHDVAFFCLHQPDSLESLNFPHLLNPSLVSRTSPRSIQQAASNAGMAIDAGIVHSTSVNSGCGDTTTYYENGADQVEPLNISVYDYLGGHDHV

>AtERF20 AT1G71520

MDSRDTGETDQSKYKGIRRRKWGKWVSEIRVPGTRQRLWLGSFSTAEGAAVAHDVAFYCLHRPSSLDDESFNFPHLLTTSLASNISPKSIQKAASDAGMAVDAGFHGAVSGSGGCEERSSMANMEEEDKLSISVYDYLEDDLV

>AtERF14 AT1G44830

MVKTLQKTPKRMSSPSSSSSSSSSTSSSSIRMKKYKGVRMRSWGSWVSEIRAPNQKTRIWLGSYSTAEAAARAYDAALLCLKGSSANNLNFPEISTSLYHIINNGDNNNDMSPKSIQRVAAAAAAANTDPSSSSVSTSSPLLSSPSEDLYDVVSMSQYDQQVSLSESSSWYNCFDGDDQFMFINGVSAPYLTTSLSDDFFEEGDIRLWNFC

>AtERF12 AT1G21910

MVKQERKIQTSSTKKEMPLSSSPSSSSSSSSSSSSSSCKNKNKKSKIKKYKGVRMRSWGSWVSEIRAPNQKTRIWLGSYSTAEAAARAYDVALLCLKGPQANLNFPTSSSSHHLLDNLLDENTLLSPKSIQRVAAQAANSFNHFAPTSSAVSSPSDHDHHHDDGMQSLMGSFVDNHVSLMDSTSSWYDDHNGMFLFDNGAPFNYSPQLNSTTMLDEYFYEDADIPLWSFN

>AtERF13 AT1G77640

MVKQELKIQVTTSSSSLSHSSSSSSSSTSALRHQSCKNKIKKYKGVRMRSWGSWVTEIRAPNQKTRIWLGSYSTAEAAARAYDAALLCLKGPKANLNFPNITTTSPFLMNIDEKTLLSPKSIQKVAAQAANSSSDHFTPPSDENDHDHDDGLDHHPSASSSAASSPPDDDHHNDDDGDLVSLMESFVDYNEHVSLMDPSLYEFGHNEIFFTNGDPFDYSPQLHSSEATMDDFYDDVDIPLWSFS

>AtERF15 AT4G31060

MPPSPPKSPFISSSLKGAHEDRKFKCYRGVRKRSWGKWVSEIRVPKTGRRIWLGSYDAPEKAARAYDAALFCIRGEKGVYNFPTDKKPQLPEGSVRPLSKLDIQTIATNYASSVVHVPSHATTLPATTQVPSEVPASSDVSASTEITEMVDEYYLPTDATAESIFSVEDLQLDSFLMMDIDWINNLI

>AtERF61 AT1G64380

MEESNDIFQNNFSPKISEIRASLSQIILAGGPNTLDSIFSLLTPSSVESATTSFNTHNPPPPPQLGSSVYLRQRDIIEKFHLQNRAISTPHPPLFSSTYDHHQTSELMLQAAAGSPAAAFAAALAAGRVTKKKKLYRGVRQRHWGKWVAEIRLPQNRMRVWLGTYDTAEAAAYAYDRAAYKLRGEYARLNFPNLKDPSELLGLGDSSKLIALKNAVDGKIQSICQRVRKERAKKSVKVSKNSSATADSSCLSSPEILSSSPVTTTTTAVTSEDSYWVSPMGLCNSENSSPVSVSVPSEVPATAEEEAMMGVDTDGFLLARMPSFDPELIWEVLAN

>PcERF61-like

MVGKMDLITDPQNSEGPDRQGFRFPSLVSNQNLGVFNASVQDVFEDSASSSSSPSNGDARLEGLDAWQNLRMPEASLFSGNSEVLCSSSSFFPNLPSNGFGHSDGLVWRPSFQGQNQYSGESIESAMVLYELLHVQQIQQIQQQQFQLQQHQTSAAASIHHMGRNPLGPRAQPMKLHGSSLSKPAKLYRGVRQRHWGKWVAEIRLPRNRTRLWLGTFDTAEEAALAYDKAAYRLRGDYARLNFPHLKHHLEANSFAPWTGNSVLPSSVDAKLQAICQSLKQPLESMSKTEESEEISCAYENSGSLGSVRDEDAKKNDVVSVKSETCDSDSSDDSTITALNSAGESESRSASKSETQAETETDTLCSMPSFSASSIWAELDDYLLSIPPLDMDINWDVLS

>PcRAP2.4-like

MERRDQSPVAARHPMRKHYRGVRQRQWGKWVAEIRLPQNRTRLWLGTFDTAEAAALAYDRAAYRWRGECARLNFPHLFSKRYQNSSPSSTNGRIPRLSCEKSDQKYAYNGDPVHTNVYKGPPIRITAYNGDPVPIDVYRSDPVRVSAYNGDPVRISAYSGDPVGNTVTLAESELESSCSHESPNTKVERFIWEEEEDENWLNDLPVLEADMTWDVLSGCSDIDTEVSQTRTCHLW*

>AtERF62 AT4G13620

MITPIHTQHSLILVYINIYSPPILSKLRTGFILWTNTQKTNKKRNMEDQFPKIETSFMHDKLLSSGIYGFLSSSTPPQLLGVPIFLEGMKSPLLPASSTPSYFVSPHDHELTSSIHPSPVASVPWNFLESFPQSQHPDHHPSKPPNLTLFLKEPKLLELSQSESNMSPYHKYIPNSFYQSDQNRNEWVEINKTLTNYPSKGFGNYWLSTTKTQPMKSKTRKVVQTTTPTKLYRGVRQRHWGKWVAEIRLPRNRTRVWLGTFETAEQAAMAYDTAAYILRGEFAHLNFPDLKHQLKSGSLRCMIASLLESKIQQISSSQVSNSPSPPPPKVGTPEQKNHHMKMESGEDVMMKKQKSHKEVMEGDGVQLSRMPSLDMDLIWDALSFPHSS

>AtERF58 AT1G22190

MTTSMDFYSNKTFQQSDPFGGELMEALLPFIKSPSNDSSAFAFSLPAPISYGSDLHSFSHHLSPKPVSMKQTGTSAAKPTKLYRGVRQRHWGKWVAEIRLPRNRTRLWLGTFDTAEEAALAYDKAAYKLRGDFARLNFPDLRHNDEYQPLQSSVDAKLEAICQNLAETTQKQVRSTKKSSSRKRSSTVAVKLPEEDYSSAGSSPLLTESYGSGGSSSPLSELTFGDTEEEIQPPWNENALEKYPSYEIDWDSILQCSSLVN

>AtERF55 AT1G36060

MADLFGGGHGGELMEALQPFYKSASTSASNPAFASSNDAFASAPNDLFSSSSYYNPHASLFPSHSTTSYPDIYSGSMTYPSSFGSDLQQPENYQSQFHYQNTITYTHQDNNTCMLNFIEPSQPGFMTQPGPSSGSVSKPAKLYRGVRQRHWGKWVAEIRLPRNRTRLWLGTFDTAEEAALAYDRAAFKLRGDSARLNFPALRYQTGSSPSDTGEYGPIQAAVDAKLEAILAEPKNQPGKTERTSRKRAKAAASSAEQPSAPQQHSGSGESDGSGSPTSDVMVQEMCQEPEMPWNENFMLGKCPSYEIDWASILS

>AtERF57 AT5G65130

MALNMNAYVDEFMEALEPFMKVTSSSSTSNSSNPKPLTPNFIPNNDQVLPVSNQTGPIGLNQLTPTQILQIQTELHLRQNQSRRRAGSHLLTAKPTSMKKIDVATKPVKLYRGVRQRQWGKWVAEIRLPKNRTRLWLGTFETAQEAALAYDQAAHKIRGDNARLNFPDIVRQGHYKQILSPSINAKIESICNSSDLPLPQIEKQNKTEEVLSGFSKPEKEPEFGEIYGCGYSGSSPESDITLLDFSSDCVKEDESFLMGLHKYPSLEIDWDAIEKLF

>AtERF56 AT2G22200

METASLSFPVPNTSFGVNKSMPLGLNQLTPYQIHQIQNQLNHRRSTISNLSPNRIRMKNLTPSTSKTKNLYRGVRQRHWGKWVAEIRLPKNRTRLWLGTFETAEKAALAYDQAAFQLRGDIAKLNFPNLIHEDMNPLPSSVDTKLQAICKSLRKTEEICSVSDQTKEYSVYSVSDKTELFLPKAELFLPKREHLETNELSNESPRSDETSLLDESQAEYSSSDKTFLDFSDTEFEEIGSFGLRKFPSVEIDWDAISKLANS

>AtERF60 AT4G39780

MAAIDMFNSNTDPFQEELMKALQPYTTNTDSSSPTYSNTVFGFNQTTSLGLNQLTPYQIHQIQNQLNQRRNIISPNLAPKPVPMKNMTAQKLYRGVRQRHWGKWVAEIRLPKNRTRLWLGTFDTAEEAAMAYDLAAYKLRGEFARLNFPQFRHEDGYYGGGSCFNPLHSSVDAKLQEICQSLRKTEDIDLPCSETELFPPKTEYQESEYGFLRSDENSFSDESHVESSSPESGITTFLDFSDSGFDEIGSFGLEKFPSVEIDWDAISKLSES

>AtERF54 AT4G28140

MDFDEELNLCITKGKNVDHSFGGEASSTSPRSMKKMKSPSRPKPYFQSSSSPYSLEAFPFSLDPTLQNQQQQLGSYVPVLEQRQDPTMQGQKQMISFSPQQQQQQQQYMAQYWSDTLNLSPRGRMMMMMSQEAVQPYIATKLYRGVRQRQWGKWVAEIRKPRSRARLWLGTFDTAEEAAMAYDRQAFKLRGHSATLNFPEHFVNKESELHDSNSSDQKEPETPQPSEVNLESKELPVIDVGREEGMAEAWYNAITSGWGPESPLWDDLDSSHQFSSESSSSSPLSCPMRPFF

>AtERF53 AT2G20880

MATAKNKGKSIRVLGTSEAEKKDEMELEEEFQFSSGKYKDSGPGSDMWLGDASSTSPRSLRKTRTFDRHNPYLVSSYATPQPPTTTTCSVSFPFYLPPAIQNQQRFLHPNDPSGQRQQQMISFDPQQQVQPYVAQQQQQQQHLLQYWRDILKLSPSGRMMMMNMLRQESDLPLTRPPVQPFSATKLYRGVRQRHWGKWVAEIRKPRNRTRLWLGTFDTAEEAAMAYDREAFKLRGETARLNFPELFLNKQEPTPVHQKQCETGTTSEDSSRRGEDDSSTALAVGGVSEETGWAEAWFNAIPEEWGPGSPLWDDYHFPISNHKDDLDATQNSSSDTI

>AtABI4 AT2G40220

MDPLASQHQHNHLEDNNQTLTHNNPQSDSTTDSSTSSAQRKRKGKGGPDNSKFRYRGVRQRSWGKWVAEIREPRKRTRKWLGTFATAEDAARAYDRAAVYLYGSRAQLNLTPSSPSSVSSSSSSVSAASSPSTSSSSTQTLRPLLPRPAAATVGGGANFGPYGIPFNNNIFLNGGTSMLCPSYGFFPQQQQQQNQMVQMGQFQHQQYQNLHSNTNNNKISDIELTDVPVTNSTSFHHEVALGQEQGGSGCNNNSSMEDLNSLAGSVGSSLSITHPPPLVDPVCSMGLDPGYMVGDGSSTIWPFGGEEEYSHNWGSIWDFIDPILGEFY

>AtRAP2.9 AT4G06746

MVIQYKRKQEFPMVKEGMVMTEKPKRNLISSNEKRYKGIRMRKWGKWVAEIREPNKRSRIWLGSYKTAVAAARAYDTAVFYLRGPSARLNFPEEVFKDGNGGEGLGGDMSPTLIRKKAAEVGARVDAELRLENRMVENLDMNKLPEAYGL

>AtRAP2.1 AT1G46768

MEREQEESTMRKRRQPPQEEVPNHVATRKPYRGIRRRKWGKWVAEIREPNKRSRLWLGSYTTDIAAARAYDVAVFYLRGPSARLNFPDLLLQEEDHLSAATTADMPAALIREKAAEVGARVDALLASAAPSMAHSTPPVIKPDLNQIPESGDI

>AtHRD AT2G36450

MQGTSKDNGGRHPLYRGVRQRKNSNKWVSEIREPRKPNRIWLGTFSTPEMAAIAYDVAALALKGSQAELNFPNSVSSLPAPTSMSPADIQAAAASAAAAFGAARDAIVMANNNSQTSGVACMNSSYDNTNMNGFMDEDLVFDMPNVLMNMAEGMLLSPPRPTVFDAAYDADGFPGGDDYLWNFP

>AtORA47 AT1G74930

MVKQAMKEEEKKRNTAMQSKYKGVRKRKWGKWVSEIRLPHSRERIWLGSYDTPEKAARAFDAAQFCLRGGDANFNFPNNPPSISVEKSLTPPEIQEAAARFANTFQDIVKGEEESGLVPGSEIRPESPSTSASVATSTVDYDFSFLDLLPMNFGFDSFSDDFSGFSGGDRFTEILPIEDYGGESLLDESLILWDF

>ATCEJ1 AT3G50260

MDAGVAVKADVAVKMKRERPFKGIRMRKWGKWVAEIREPNKRSRLWLGSYSTPEAAARAYDTAVFYLRGPTATLNFPELLPCTSAEDMSAATIRKKATEVGAQVDAIGATVVQNNKRRRVFSQKRDFGGGLLELVDLNKLPDPENLDDDLVGK

>AtDREB2B AT1G75490

MSSIEPKVMMVGANKKQRTVQASSRKGCMRGKGGPDNASCTYKGVRQRTWGKWVAEIREPNRGARLWLGTFDTSREAALAYDSAARKLYGPEAHLNLPESLRSYPKTASSPASQTTPSSNTGGKSSSDSESPCSSNEMSSCGRVTEEISWEHINVDLPVMDDSSIWEEATMSLGFPWVHEGDNDISRFDTCISGGYSNWDSFHSPL

>PcCHI1.1

MKSMKFCAMAIALLTMATMNMYFVSAEQCGQQAGGALCPGGLCCSKWGWCGNTDAHCGQDCQSQCSGSTPTPGGQGVASIITESIFNELLKHRNDAGCKASGFYTYSAFIAAANAFPSFGTTGDVATRKRELAAFFGQTSHETTGGWATAPDGAYAWGYCFKEEQGNPPAEYCQATSQWSCASGKRYYGRGPVQLSWNYNYGPAGKAIGFDGINNPDIVASDATVSFKTAIWFWMTAQSPKPSCHDVMTGKWTPSGSDSAAGRAAGYGAVTNIINGGLECGKGSDSRQQDRIGFYKRYSDILGVSYGSNLDCNNQRPFGAAVQSEPRLIKTVV

>PcCHI2.2

MAYTNMGRRMSIMRLLLALTAVAIMSSLCCYVSAQQGVASIISEDVFNQFLKHRNDAACSAKGFYTYTAFIAAANSFPDFGNNGDLESRKRELAAFFGQTSQETTGGWATAPDGPYAWGYCFKDQVNSGDRYHGRGPIQLTGDYNYKAAGDALGYDLINNPDLVVNDATISFKTAVWFWMTAQSPKPSCHDVILGRWSPSATDTAAGRVAGYGMVTDIINGGPECGTGTISDVQQGRIGFYQRYCNMLGVDVGSNLDCKNQKPFGT

>PcCHI2.1

MARKMSMKLLLALAAVAIMSTLCYVSEQQGVGSIITEDVFNEFLKHRNDAACQARDFYTYSAFIAATNSFSDFGNNGDLESRKRELAAFFGQTSQETTGGWATAPDGPYAWGYCFKEENSGDKYHGRGPIQLTGDYNYKAAGDALGYDLINNPDLLVTDATVSFKTAVWFWMTAQAPKPSCHDVILGRWSPSNDDTAAGRVPGYGLLTNIINGGMECGTGTISDRQQGRIGFYQRYCSLLGVDTGSNLDCQNQKHF

>PcCHI4.1

MVLVVVLVLLLAGVSVNAQNCGCASGLCCSKFGYCGTTSAYCGDGCQSGPCTSSGGGSPSGGGGSVGTIISQSFFNGLAGGAASSCEGKGFYTYDAFIAAANAYSGFGTTGSADVTKRELAAFFANVMHETGGMCYINERTPPMIYCMSSATWPCASGKDYHGRGPLQLSWNYNYGAAGKNIGFDGVNNPEKVGQDSTISFKTAVWFWMDNSNCHTAITSGQGFGGTIKAINSQECNGGNSGEVNSRVNYYKNICSQLGVDPGANVSC

>Pg chitinase AAA85364.1 (IV)

MGSSSSDKSVMALVLVLLLVGVSVNAQNCGCASGVCCSQYGYCGTTSAYCGKGCKSGPCYSSGGGSPSAGGGSVGGIISQSFFNGLAGGAGSSCEGKGFYTYNAFIAAANAFSGFGTTGSNDVKKRELAAFFANVMHETGGLCYINEKNPPMKYCQSSSTWPCTSGKSYHGRGPLQLSWNYNYGAVGKSIGFDGLNNPEKVGKDPTTSSKTAVWFWMKNRNCHSAITSGKGLGGTIKAINSMECNGGNSGEVNSRVNYYKKICSQLGVDPGANVSC

>Pa chitinase AAQ17048.1 (IV) MGSIIIDKSVMALVLLLLLVCVTVNAQNCGCATGVCCSQYGYCGTTSAYCGKGCKSGPCYSSGGKSPIAGGGSVGGIISQSFFNGLAGGAGSSCEGKGFYTYNAFIAAANAYSGFGTTGSNNVKKRELAAFFANVMHETGGLCYINEKNPPINYCQSSSTWPCTSGKSYHGRGPLQLSWNYNYGAAGKSIGFDGLNNPEKVGQDSTISFKTAVWFWMKNSNCHSAITSGQGFGGTIKAINSMECNGGNSGEVSSRVNYYKKICSQLGVDPGANVSC

>Pm chitinase ACY06320.1 (IV) MGRTGGEKWVMALVLVLLLVGVGVNAQNCGCASGLCCSKFGYCGTTSAYCGTGCQSGPCSSSGGGSPSGGGGSVGTIISESVFNGLAGGAASSCEGKGFYTYNAFIAAASAYSGFGTTGSSDVQKRELAAFFANVMHESGGLCYINEINPPIIYCQSSSTWPCTSGKSYHGRGPLQLSWNYNYGAAGQSIGFDGLNNPEKVGQDATISFKTAVVFWMKNSNCHSAITGGQGFGATIKAINSGECNGGNSGEVSSRVNYYKKICSQLGVDPGANVSC

>Pm chitinase 2-2 ACY06318.1 (II) MVIHLRVMTASVFVLWLCLALSNCSGDVGYITTEAFFNGILSGAADTCEAKNFYTYSDFITAANAFSGFGTTGNFDDKKREIAAFFANVAHETIGFCYIEEIVKGVYCDSNNTQYPCAAGKEYYGRGPIRLSWNFNYGGAGNYLGCDLLNKPEIVAQNDLIAWMTALWYWNVASDCHSAITSGKGFGATIRAINGAIECNGGNTGEVNDRISYYKKYCTQFGVDPGSNLSC

>Pm chitinase 2-1 ACY06317.1 (II)

MATQYLPVSVIALWLTLALSALSISRAAVGDIATQSFFNGILSTAADSCAGKTFYTYSDFINAANAFSAFGTTGTSDDQKREIAAFFANVAHETGSLCYIEEIDKSDSYCDSTNTQYPCVSGKQYYGRGPLQLTWNYNYGAAGDYLGSDLLNNPETVAQDDLISWKTALWFWNVNTGSVGTTCHAAITSGQGFGETIRIINGGVECDGKSPSSVQDRVSLYTNYCSQLGVDPGSNPSC

>St chitinase AAB96340.1 (II)

FLTETLAQNAGSIVTRELFEQMLSFRNNDVCPGKGFYTYDAFIAAANSFPAFGTTGDDTARKKEMAAFFGQTSHETNGGSAGTFTGGYCFVRQIDQSERYYGRGPIQLTHQSNYERAGQGIGVGQDLVNNPDLVATDAIISFKTAIWFWMTAQDNKPSCHNVIIGQWTPSPADTAANRVPGYGVITNIINGGLECGMGQNTAVESRIGFYRRYCGMLNVPTGENLDCNNQRNFAQG

>Sc endochitinase AAG53610.1 (II)

MARFAALAALLLAVAVGGAAAQSVGSVITQSMYASMLPNRDNSLCPARGFYTYDAFIAAANTFPGFGTTGSTDDVKREVAAFFGQTSHETTGGTRGAADQFQWGYCFKEEINKATSPPYYGRGPIQLTGRSNYDLAGRAIGKDLVSNPDLVSTDAVVSFRTAMWFWMTAQGNKPSSHDVALRRWTPTAADNAAGRVPGYGVITNIINGGLECGMGRNDANVDRIGYYTRYCGMLGTATGGNLDCYTQRNFAS

>Nt chitinase CAA34812.1 (I)

MRLCKFTALSSLLFSLLLLSASAEQCGSQAGGARCPSGLCCSKFGWCGNTNDYCGPGNCQSQCPGGPTPTPPTPPGGGDLGSIISSSMFDQMLKHRNDNACQGKGFYSYNAFINAARSFPGFGTSGDTTARKREIAAFFAQTSHETTGGWATAPDGPYAWGYCWLREQGSPGDYCTPSGQWPCAPGRKYFGRGPIQISHNYNYGPCGRAIGVDLLNNPDLVATDPVISFKSALWFWMTPQSPKPSCHDVIIGRWQPSAGDRAANRLPGFGVITNIINGGLECGRGTDSRVQDRIGFYRRYCSILGVSPGDNLDCGNQRSFGNGLLVDTM

>Hv endochitinase P23951.1 (II)

SVSSIVSRAQFDRMLLHRNDGACQAKGFYTYDAFVAAAAAFPGFGTTGSADAQKREVAAFLAQTSHETTGGWATAPDGAFAWGYCFKQERGASSDYCTPSAQWPCAPGKRYYGRGPIQLSHNYNYGPAGRAIGVDLLANPDLVATDATVGFKTAIWFWMTAQPPKPSSHAVIAGQWSPSGADRAAGRVPGFGVITNIINGGIECGHGQDSRVADRIGFYKRYCDILGVGYGNNLDCYSQRPFA

>Sc endochitinase AAG53609.1 (I)

MRGVVVVAMLAAAFAVSAHAEQCGSQAGGATCPNCLCCSKFGFCGSTSEYCGDGCQSQCNRCGGTPVPVPTPTGGGVSSIISQSLFDQMLLHRNDAACLAKGFYNYGAFIAAANSFSAFATTGGTDVRKREVAAFLAQTSHETTGGWPTAPDGPYSWGYCFNQERGAPSDYCSPSSQWPCAPGKKYFGRGPIQISYNYNYGPAGRAIGTDLLNNPDLVATDATVSFKTALWFWMTPQSPKPSSHDVITGRWSPSGADQAAGRVPGYGVITNIINGGLECGRGQDARVADRIGFYKRYCDLLGVSYGHNLDCYNQRPFA

>Zm chitinase AAA62421.1 (I)

MMRALAWWPCWPAFFAVPARAEQCGSQAGGALCPNCLCCSQFGWCGSSDYCGSGCQSQCSAACSTPNPPSSGGVASIIPESLFNQMLLHRNDAACPANGFYTYAGFIAAANAFPGLAPRVRPDVQKRELAAFLAQTSHETTGGWATARRPYAWGYCFKEEQGGASGPDYCEPSAQWPCAAGKKYYGPRAIQISININIGPAGRPSAPGILANPDLVATRPHRVVRRPPSGSGMTPQSPKPSCTDVMTGQWTPSAADTAAGRLPGYGVVTNISNGGLECGHGADSRVADRIGFYKRYCDLLGVSYGDNLDCANQTPFNG

>Gh chitinase AAP80801.1 (II)

MADFKKATALILTVALLVNLALMVDADGDDDKKIRVRKHKGEKQCIQGWECSYWSKYCCNKTVSDVFQVYQFEDLFAKRNSPVAHAVGFWDYHSFILAASIYEPLGFGTTGGKRMQMKEVAAFLAHVGAKTSCGDGVIDGGPLAWGLCFKREMSPSQDYCDDYYKYMYPCAPGAQYYGRGALPIYWNYNYGAAGDGIKVDLLHHPEYLEQNATIAFQAAIWRWMTPIKKNQPSAHDIFVGNWKPTKNDTEEKRGPTFGSTMNVLYGDYTCGQGDIDPMNIIISHYLHYLDLLGVGREEAGPHEELSCAEQKAFNPTPAPPAASAS

>At chitinase BAA94976.1 (II)

MVTLIDEEKDKKKSTMVSKPLFSLLLLTVALVVFQTGTLVNAEDSEPSSSTRKPLVKIVKGKKLCDKGWECKGWSEYCCNHTISDFFETYQFENLFSKRNSPVAHAVGFWDYRSFITAAAEYQPLGFGTAGEKLQGMKEVAAFLGHVGSKTSCGYGVATGGPLAWGLCYNKEMSPDQLYCDDYYKLTYPCTPGVSYHGRGALPVYWNYNYGQTGEALKVDLLSHPEYLENNATLAFQAAIWRWMTPPKKHLPSAHDVFVGKWKPTKNDTAAKRTPGFGATINVLYGDQICNSGFDNDEMNNIVSHYLYYLDLIGVGREEAGPHEKLSCADQEPFSSSSSAPPSSGSSS

>At putative chitinase CAB78983.1 (V)

MSSTKLISLIVSITFFLTLQCSMAQTVVKASYWFPASEFPVTDIDSSLFTHLFCAFADLNSQTNQVTVSSANQPKFSTFTQTVQRRNPSVKTLLSIGGGIADKTAYASMASNPTSRKSFIDSSIRVARSYGFHGLDLDWEYPSSATEMTNFGTLLREWRSAVVAEASSSGKPRLLLAAAVFYSNNYYSVLYPVSAVASSLDWVNLMAYDFYGPGWSRVTGPPAALFDPSNAGPSGDAGTRSWIQAGLPAKKAVLGFPYYGYAWRLTNANSHSYYAPTTGAAISPDGSIGYGQIRKFIVDNGATTVYNSTVVGDYCYAGTNWIGYDDNQSIVTKVRYAKQRGLLGYFSWHVGADDNSGLSRAASQAWDATTATTRTIQKV

>Nt chitinase CAA54374.1 (V)

MANSVTLFAIIFSCFLLQQLVCTNSQNVKGGYWFKDSGLALNNIDSTLFTHLFCAFADLNPQLNQLIISPENQDSFRQFTSTVQRKNPSVKTFLSIAGGRANSTAYGIMARQPNSRKSFIDSSIRLARQLGFHGLDLDWEYPLSAADMTNLGTLLNEWRTAINTEARNSGRAALLLTAAVSNSPRVNGLNYPVESLARNLDWINLMAYDFYGPNWSPSQTNSHAQLFDPVNHVSGSDGINAWIQAGVPTKKLVLGIPFYGYAWRLVNANIHGLRAPAAGKSNVGAVDDGSMTYNRIRDYIVESRATTVYNATIVGDYCYSGSNWISYDDTQTVRNKVNYVKGRGLLGYFAWHVAGDQNWGLSRTASQTWGVSFQEMK

>Os chitinase AAM08773.1 (III)

MVNGYLFREYIGAQFTGVRFSDVPVNPGLSFHFILAFAIDYFMATQSSKPAPANGVFAPYWDTANLSPAAVAAAKAAHPNLSVILALGGDTVQNTGVNATFAPTSSVDAWVRNAADSVSGLIDAYGLDGVDVDYEHFAAGVDTFVECIGRLLTELKARHPNIATSIAPFEHPVVQRYYQPLWRRYAGVIDYVNFQFYGYGANTDVATYVMFYDEQAANYPGSKLLASFKTGNVTGLLSPEQGIAGAKELQRQGKLPGLFIWSADSSMVSSYKFEYETKAQEIVANH

>Sr chitinase homologue CAA88593.1 (III)

MAPKRQALILIVLSLLTINTSEAATGGIAIYWGQNNGDGTLTSTCDTGNYEIVVLSFLTTFGCSRTPQWNFAGHCGDWSPCTKLQPEIQHCQQKGVKVFLSLGGASGSYSLCSPQDAKEVADYLFSNFLTGRYGPLGSVTLDGIDFDIEGGSNLYWDDLAKELDALRQTNNYFYLSAAPQCPIPDYYLDKAIKTGLFDYVFVQFYNNPPCQYSNGNTGPLLGSWDAWTSLVLPNNTVFMGLPASREAAPSGGYIPPNVLISEVLPYIKQASNYGGIMLWSRFQDVTNHYSDQIKYYVTKYMLRFVKAVSNAISDCVSAALHRFLPKPY

>Ms_gi|22901738(III)

TRLLLLLVLTIFPFTIKASSSGGIAIYWGQNLGDGTLTSTCDTGNYEIVLLAFLNVFGGGRVPNWNFAGHCGDWSPCTKLEPEIKHCQQKGVKVLLSIGGAVGSYSLSSPEDAKNVADYLHTNFLSGQFGPLGSVTLDGVDFDIEGGTNLYWDDLARDLDNLRQQNRYFYLSAAPQCFMPDYYLDKAIKTGLFDYVLVQFYNNPPCQYDIKNSDPKLLLQSWNAWTSLVLPNNTVFMGLPAAPNAAPSGGYIPPDDLISKVPPSIKPTSNYGGIMLWDRFHDVTGNNDYIYSDQIKEHVKRSVLRFKTQVSEAISRCISAALNPMLPN

>Nt acidic chitinase CAA77656.1 (III)

MIKYSFLLTALVLFLRALKLEAGDIVIYWGQNGNEGSLADTCATNNYAIVNIAFLVVFGNGQNPVLNLAGHCDPNAGACTGLSNDIRACQNQGIKVMLSLGGGAGSYFLSSADDARNVANYLWNNYLGGQSNTRPLGDAVLDGIDFDIEGGTTQHWDELAKTLSQFSQQRKVYLTAAPQCPFPDTWLNGALSTGLFDYVWVQFYNNPPCQYSGGSADNLKNYWNQWNAIQAGKIFLGLPAAQGAAGSGFIPSDVLVSQVLPLINGSPKYGGVMLWSKFYDNGYSSAIKANV

>Pc.isotig40157

STLTKLFVAASSAYSEFGTTGSSDDQKRELAAFFANVMHETGGLCYINEVNPGSIYCDSSSNTWPCASGKSYHGRGPLQLSWNYNYGAAGEDIGFDGLNHPEEVALDAIISFRTGVWFWMKNSNCHSAITSGQGFGATIKAINSMECNGGNTAAVNSRINYYTKFCS

>Pc.isotig26389

CGDGCQSGPCTSSGGGSPSTGGGNVGTIISQSVFDGLAGGAASSCEGKGFYTYDAFIAAANAYSGFGTTGASDDQKRELAAFFANVMHETGGLCYINEQNPPSIYCDATSTSWPCASGKSYHGRGPLQLSWNYNYGVAGQNIGFDGLNNPDVVGQDATISFKTAVWFWMQNCHSAITSGQGFGATIKAINSMECNGGNTPAVNSRVSYYNTICSQLGVDPGANVSC

>Pc.isotig26276

MASNASDRKVFINSAIAIARKYGFHGLDLDWEFPKNSVEMENLGTLFSEWRHAIDLEATASGRPRILLTAAVYFAQYFFLSSEKRAYPATSIAQNLDWVNVMCYDYHGSWNISATGAHAALYDPSSNISTSFGIGSWLHSGVSPNNIVMGMPLYGRSWILKSLDETGIGAPAMAAGPKQTLSNEKGVMFFSEIRDLINQKNATEVFDKDTVSAYSYSSGMIWVAYDNPDSVATKVSFAKERRLLGYFFWGYQSR

>Pc.isotig24918

MAAQLPVSAIILWFVFALSALSICRGAVSDIATEDFFNGILSAATDGCAGKTFYTYSDFINAANSFSSFGTTGTSDDNKREIAAFFGNVAHETTNLCFVEENAKSDNCDSSNTQYPCASGQQYYGRGPLQLTGNGNYGAAGSYLGVDLLNNPGLVAQDDLTSWKTALWFWNVNSNCHTAITSGQGFGATIQAINGAKECNGVNTAEVNDRVSRYTTYCSQLVVDPGSNLSC

>Pc.isotig24895

MRNYLRVMTPSVIILWLVLAFDLVSICRGDVGDFATQDFFNGILSGASDSCAGKNFYTYNNFMDAATAFSGFGTTGPDVDHKREIAAFFANVAHETSRLCYVEQIEKSDYCDSTNEKYQCVAGKQYYGRGPLQLTWNYNYDAAGDYLGFDGLNHPEIVAQNGSISWKTAVWFWMKHSNCHSAITSGQGFRATIKAISGDECNGGDSNAVDERVNYYTNYCNEFGVDPGNNLSC

>Pc.isotig23736

MGVNGKLFADYIGALFKGVKFSDVPINSAVEFHFILAFAIDYSTESGSPVYTDGNFNIYWDTGNLSPEAVEAIKAQHTNVKVALSLGGDTVDSQPVEFKPSSVSAWVDNAVSSLSKIIKEYHLDGIDIDYEHFQANNDIFAECIGQLITRLKQSDAISFASIAPYDNQEVQSHYLGLWNKYGHVIDYINFQFYAYDSSTTIAQFKEYYNTQASQYSGGKVLVSFSSATGGGGLLPVNGFFEACKMLKQNGELEGIFVWCADCSKANGFEYEIESQDLLAAS

>Pc.isotig23657

NMGRRMSIMRLLLALTAVAIMSSLCCYVSAQQGVASIISEDVFNQFLKHRNDAACPAKGFYTYTAFIAAANSFPDFGNNGDLQSRKRELAAFFGQTSQETTGGWATAPDGPYAWGYCFKDQLKSGDRYHGRGPIQLTGDYNYKAAGDALGYDLINNPDLVVNDATISFKTAVWFWMTAQSPKPSCHDVILGRWSPSATDTAAGRVAGYGMVTDIINGGPECGTGTISDVQKGRIGFYQRYCNMLGVAVGSNLDCKNQKPFGT

>Pc.isotig21128

METKQVNRILVVAGICLMMSSWFCCIEAAAAAAAADENEETKFKKILCAKATDCKNKTISELFTVDQFESLFSHRNAPLAHAQGFWDYHSFITAAAHYEPKGFGTTGGDIVQKRELAAFFAHIATETSCESLMAQAASTAPSDSPTKWGLCYKEELSPDSTYCESSLVYPCAPGVSYHGRGALPVYWNYNYGQLGQALKVDLLHHAEYLSQNATLAFAAAIWRWMTPMKVKQPSAHQVMVGKWVPTKNDTEALRLPGFGMTINILKADAECGTDSDDKQMNTRIAHYLDFLDHMDVGRENAGDNVDCSEQKVLNPSSSAST

>Pc.isotig20095

MAQAPKNLCIILFFITSSIIYSSSSSNNTTSNNGVKAAYWPSDSSLSPSSISTDLFTHIFYAFADLNDQTFQVQLPATADPAEFTSTLLQKNPSLKTLISIGGGGSNATAFALMASNASHRKVFINSAIAVARKYGFHGLDLDWEFPRDAAEMEYLGTLFDDWRHAIDLEATASGRPRLLLTAAVYFAQYFFVWGKKRAYPVTSIARNLDWVNVMCYDYHGSWDISATGAHAALYDPTSNISTSFGIGSWLHSGVPPNKVAMGMPLYGRSWILKSLDETEIGAPAVAAGPKQTLSNEKGVMFFSEIRELINQKNATEVFDKETVSAYSYSSDLLWVGYDNQESVATKVSFAKEMHLLGYFFWAIGQDNNWMLSAQASDSWN

>Pc.isotig10629

MAMGRICLVVLLALALCLNVSLASKKMKVCDKGWECKGTYCCNQTISEIFTVDNFEELFSKRNTPVAHAVGFWDYYSFINAAAQFEGIGFGTTGGQVMQQKELAAFLGNVAAETSCGYNVATGGPTAWGLCYKEEMSPDQLYCDQNLLYPCAPGASYHGRGALPIYWNFNYGPIGEALKLDLLTSPDMVSNNATIGFLTAMWRWMNPIKPKQPSAHDVFVGNWKPTKNDTESYRLPGFGMVINVLNGGLECGKGDIDAMNNRISHYLYFLDLLGVGREQAGDNLDCGQQVPLNPVSTSTTSR

>Pc.isotig10072

MGTRTGRSVIVSPYEHMVLRMSMVLLLVVVVSVNVVYAQNCGCASGLCCSKWGYCGTTSAYCGNGCQSGPCYGSGGGSPSGGGGNVGTIISQNFFSGLANAAGSSCEGKGFYTYNAFITAANAYSGFGTTGASDNQKRELAAFFANVMHETGGLCYINEINPLSNFCQSSSTWPCASGKSYHGRGPIQLTWNYNYGAAGQDIGFDGLNNPEKVGQDATISFKTAVWFWMKNSNCHSAITSGQGFGATIKAINGGECNGGHSAQVNSRVNYYKKLCSQLGVDPGTNISC

>Pc.isotig09774

MAQTQKYLLFILLSMTTSLVYSSSSQSASHGGGVKAAYWPSYAVSYSPPSSINAALFTHIFYAFANLNDQTFQVELPATAKPAEFTLTLRKKNPFLKTLISIGGGGSNSTAFALMASNASRRKVFINSAIALARKYGFHGLDLDWEFPKDATEMQNLGSLFAEWRHAIELEARAWPRCPRLLLTAAVYFAQYFLLAADKRAYPATSITQNLDWVNVMCFDYHGSWDTSATGAHAALYDPSSNISSSFGIGSWLHSGVPTNKVVMGMPLYGRSWILKSLDETGIGAPAIAAGPKQTLSSETGVMFFSEIIDFISQKNATEVFDKETVSAYAYSSDMMWVGYDNKQSVAAKVSFAKNKHLLGYFFWAISQDSNWMLSAQASVSWNQAR

>Pc.isotig06999

MEGTIGKMSSILLATTLAIFTAMTIVITGLPSVSADDCGKNAGGALCPGGLCCSKYGYCGNTQAHCGKDCQSQCGGGGSTPTPTPPTPTPTTPTPTPSGQGVASIITEDLFNQLLKYKDDSRCKANGFYSYDAFINVANAYPGFGTAGDVTSNKRELAAFFGQTSHETTGGWPAAPDGPYAWGYCLKEEIGTPPPVYCNETAQWPCGSGKSYYGRGPIQISWNYNYGPAGNALGFDGINNPDIVASDATVSFKTAIWFWMTAQSPKPSCHDVMTGKWTPSGSDSAAGRVPGYGAVTNIINGGLECGKGTVSRQQDRIGFYQRYCDILGVSYGANLDCNNQTPFGS

>Pc.isotig06997

TIGKMSSILLATTLAIFTAMAIMITGLPSVSAEDCGQQAGGAVCPRGLCCSKWGWCGNTEAHCGLGCQSQCGGGGSTPTPTPPTPTPTNPTPTPSGQGVASIITEDLFNQLLKYKDDSRCKANGFYSYAAFIAAANAFPGFGTASDVTTNKRELAAFLGQTSHETTGGWQTAPDGPYAWGYCFKEEQGTPPPVYCQASSQWSCVSGKSYYGRGPIQLTWNYNYGPAGKANRIRWHKQPRHCC

>Pc.isotig06604

MGSSSGNSVMVVVVLVLLLVSVSVNAQNCGCASGLCCSKYGYCGTTSAYCGAGCKSGPCSSSGGGSPSGGGGSVGTIISQSFFNGLAGGAASSCEGKGFYTYNAFIAAANAYSGFGTTGSADVRKRELAAFFANVMHETEGMCYINEINPQSNYCNSSATWPCASGKSYHGRGPLQLSWNYNYGAAGQTIGFDGVNNPEKVGQDPTISFKTAVWFWMKNSNCHSAITSGQGFGGTIKAINSQECNGGKTGEVNNRVNYYKNICSQLGVDPGANVSC

>Pc.isotig06603

MGGSSGNSVMVLVMVLVLLLAGVSVNAQNCGCASGLCCSKYGYCGSTPAYCGRGCRSGPCSSSGGGSPSGGGGSVGTIISQSFFNGLAGGAASSCEGKGFYTYDAFIAAANAYSGFGTTGSADVTKRELAAFLANVMHETGGMCYINERNPPMNYCMSSATWPCASGKSYHGRGPLQLSWNYNYGAAGQTIGFDGVNNPEKVGQDPTISFKTAVWFWMKNSNCHSAITSGQGFGGTIKAINSQECNGGNSGEVNSRVKYYKNICSQLGVDPGANVSC

>Pc.isotig06602

MGSSSGNSVMVVVVLVLLLVSVSVNAQNCGCASGLCCSKYGYCGTTSAYCGAGCKSGPCSSSGGGSPSGGGGSVGTIISQSFFNGLAGGAASSCEGKGFYTYNAFIAAANAYSGFGTTGSADVRKRELAAFFANVMHETGGMCYINERNPPMNYCMSSAMWPCASGKSYHGRGPLQLSWNYNYGAAGKSIGFDGVNNPEKVGQDPTISFKTAVWFWMKNSNCHSAITSGQGFGGTIKAINSQECNGGNSGEVNSRVKYYKNICSQLGVDPGANVSC

>Pc.isotig05752

MKSMKFCAMAIALLTMATMNMYFVSAEQCGQQAGGALCPGGLCCSKWGWCGNTDAHCGQDCQSQCGGSTPTPGGQGVASIITESIFNELLKHRNDAGCKASGFYTYSAFIAAANAFPSFGTTGDVATRKRELAAFFGQTSHETTGGWATAPDGAYAWGYCFKEEQGNPPAEYCQATSQWPCASGKRYYGRGPVQLSWNYNYGPAGKAIGFDGINNPDIVASDATVSFKTAIWFWMTAQSPKPSCHDVMTGKWTPSGSDSAAGRAAGYGAVTNIINGGLECGKGSDSRQQDRIGFYKRYSDILGVSYGSNLDCNNQRPFGAAVQSEPRLIKTVV

>Pc.contig5089

MASHLPVTTVSLFIIGLALAFFTCRGDVENIATEDFFNGIISGVSDTCEAKNFYTYSDFITAANVFSGFGTTGTSDDEKREIAAFFANVADETIGFCYINEIQKGDYCDSNSTQYPCAPGKQYYGRGPIQLSWNFNYGPAGNYLGVDLLNHPEIVAQDALISWKSALWYWNVNSDCHSSITSGKGFGATIQAINGAIECNGGNTDEVNDRISYYTKYCSQFGVDPGSNLSC

>Pc.contig453

MAAQLPVSAIILWFVFALSALSICRGAVSDIATEDFFNGILSAATDGCAGKTFYTYSDFINAANSFSSFGTTGTSDDNKREIAAFFGNVAHETTNLCFVEENAKSDNCDSSNTQYPCASGQQYYGRGPLQLTGNGNYGAAGSYLGVDLLNNPGLVAQEDLTSWKTALWFWNVNSNCHTAITSGQGFGATILAINGAVECNGGNTAEVNDRVSRYTTYCRQLGVDPGSNLSC

>Pc.contig257

MATHFTVRAVILWFVFALSALSICRGAVSDIATQDFFNGILSAAADGCAGKTFYTYSDFINAANSFSSFGTTGTSDDNKREIAAFFANVAHETTDLCYVEEIAKSAYCDSTNTQYPCASGQQYYGRGPLQLTGNANYGAGGAYLALDLLNNPGLVAQDDLTSWKTALWFWNVNSNCHTAITSGQGFGATIQAINGAVECNGGNTDEVNDRVSRYTTYCSQLGVDPGSNLTC

>Pc.contig1840

MHSLASAQLGLPMMRREKLPLSSPTLLTKLSKGDYCDSNSTQYPCAPGKQYYGRGPIQLSWNFNYGPAGNYLGVDLLNHPEIVAQDALISWKSALWYWNVNSDCHSAITSGKGFGATIQAINGAIECNGGNTDEVNDRISYYTKYCSQFGVDPGSNLSC

>Pb.isotig31508

TASWDISATGAHAALYDPSSNVSTSFGIGSWLHSGVPPNKVVMGMPLYGCTWILKSLNETGIGAPAVAAGPKLTLSNETGVMFFSDIRNFITQKNVTVVFDNETVSAYAYSSDMMWVGYDNPDSVAIKVSFAKERRLLGYFFWAVSQDSNWMLSTRALETWNQVQ

>Pb.isotig30625

ALGYDLINNPDLLVTDATVSFKTAVWFWMTAQAPKPSCHDVILGRWSPSNDDTAAGRVPGYGLLTNIINGGEECGTGTISDRQQGRIGFYQRYCSLLGVDTGSNLDCQNQKHF

>Pb.isotig26611

SRKRELAAFFGQTSQETTGGWATAPDGPYAWGYCFKDQVNSGDRYHGRGPIQLTGDYNYKAAGDALGYDLINNPDLVVNDATISFKTAVWFWMTAQSPKPSCHDVILGRWSPSATDTAAGRVAGYGMVTDIINGGPECGTGTISDVQKGRIGFYQRYCNMLGVDVGSNLDCKNQKPFGT

>Pb.isotig24434

QDFFNGILSAAADSCAGKTFYAYSDFINAANSFSSFGTTGTSDDNKREIAAFFANVAHETGNLCYVEEINKSDYCDSTNTQYPCAAGKQYYGRGPLQLSWNYNYGAAGDYLGQDLLNNPDTVAQDDLTSWKTALWFWNVNSNCHTAITSGQGFGATIQAINGAIECNGGNTDQVNDRISRYTNYCSQFNVDPGSNLSC

>Pb.isotig24240

THIFYAFAHLNNQTFEVEVLSTDDPAEFTSTLLMKKPSLKTLLSIGGGGSSPTVFALMANNASDRKVFINSAIAIARKYGFHGLDLDWEFPKNSVEMENLGTLFSEWRHAIDLEATASGRPRILLTAAVYFAQYFFLSSEKRAYPATSIAQNLDWVNVMCYDYHGSWNISATGAHAALYDPSSNISTSFGIGSWLHSGVSPNNIVMGMPLYGRSWILKSLDETGIGAPAMAAGPKQTLSNEKGVMFFSEIRDLINQKNATEVFDKDTVSAYSYSSDMIWVAYDNPDSVATKVSFAKER

>Pb.isotig23529

MGVRCGTSVMALVLVLLLVGVSVNAQNCGCASGLCCSKYGYCGTTSDYCGDGCQSGPCTSSGGGSPSTGGGNVGTIISQSVFDGLAGGAPSSCEGKGFYTYDAFIAAANAYSGFGTTGASDDQKRELAAFFANVMHETGGLCYINEQNPASIYCDATSTSWPCASGKSYHGRGPLQLSWNYNYGVAGQNIGFDGLNNPDVVGQDATISFKTAVWFWMQNCHSAITSGQGFGATIKAINSMECNGGNTPAVNSRVSYYNTICSQLGVDPGANVSC

>Pb.isotig22805

MATHFTVRAVILWFVFALSALSICRGAVSDIATQNFFNGILSAATDGCAGKTFYKYSDFINAANSFSSFGTTGTSDDNKREIAAFFANVAHETTNLCYVEEIVKSAYCDSTNTQYPCASGQQYYGRGPLQLTGNANYGAAGAYLAVDLRNNPGLVAQDDLTSWKTALWFWNVNSNCHTAITSGQGFGATIQAINGAVECNGGNTAEVNDRVSRYTTYCSQLGVDPGSNLTC

>Pb.isotig22541

KAFIDSSIALARNYSFHGLDLDWEYPQDTTEMNNLGQLLSEWRSAVQAEATATGNDPLLLTAAVYFSHSFFVSDIVREYPSEMDKLDWLNIMAYDFHGSWEPKQTGAPAALYDPDSNISTSFGVQSWLDAGLPSSKVVMGMPLYGYSWKLQSAGEVGIGAAASGTGIKDGSVTYSDIRDFIAQNAATEVFDNTTVSAYCYSGLDWIGYDNDQSVARKAVFAKQEGLLGYFFWNVVQDTADWALSRTASNTWDG

>Pb.isotig22381

MGVNGKLFADYIGALFKGVKFSDVPINSAVEFHFILAFAIDYSTESGSPVYTDGNFNIYWDTGNLSPEAVEAIKAQHTNVKVALSLGGDTVDSQPVEFKPSSVSAWVDNAVSSLSKIIKEYHLDGIDIDYEHFQANNDIFAECIGQLITRLKQSDAISFASIAPYDNQEVQSHYLGLWNKYGHVIDYINFQFYAYDSSTTIAQFKEYYNTQASQYTGGKVLVSFSSATGGGGLLPVNGFFEACKMLKQNGELEGIFVWCADWSKANGFEYEIESQDLLAAS

>Pb.isotig22332

MVLRMSMVLLLVVVVSVNVVYAQNCGCASGLCCSKWGYCGTTSAYCGNGCQSGPCYGSGGGSPSGGGGNVGTIISQNFFSGLANAAGSSCEGKGFYTYNAFIAAANAYSGFGTTGASDNQKRELAAFFANVMHETGGLCYINEINPSSNYCQSSSTWPCASGKSYHGRGPIQLSWNYNYGAAGQDIGFDGLNNPEKVGQDATISFKTAVWFWMKNSNCHSAITSGQGFGGTIKAINSGECNGGNSAQVNRRVNYYKKFCSQLGVDPGTNISC

>Pb.isotig21783

MGANGLLFSDYIGALFRNVTFSDVPINPAVEFHFILAFVIDYSTDSGSPVPKNGNFSIYWDTGNLTPQAVQSIKAQHKNVKVALSVGGDSINGQPVEFKPSSVSSWVDNAVSSLSKIIEEYHLDGIDIDYEHFQADNNTFTECIGQLITRLKRSKVISFASIAPFDNQDVQSHYLALWKRYGHAIDYVNFQFYAYSANTTISQFIKYYNKQAALYTGGKVLISFTSANGGGGLSPKNAFFEAARELKQSGKLEGIFLWCADWSKVNNVFKYESQSEALLAS

>Pb.isotig21624

MAIMITGLPSVSAEDCGQQAGGAVCPRGLCCSKWGWCGNTEAHCGLGCQSQCGGGGSTPTPTPPTPTPTNPTPTPSGQGVASIITEDLFNQLLKYKDDSRCKANGFYSYAAFIAAANAFPGFGTAGDVTTNKRELAAFLGQTSHETTGGWQTAPDGPYAWGYCFKEEQGSPPPVYCQASSQWPCVSGKSYYGRGPIQLTWNYNYGPAGKAIGFDGINNPDIVASDATVSFKTAIWFWMTAQSPKPSCHDVMTGKWTPSGSDSAAGRVPGYGAVTNIVNGGLECGKGTDSRQQDRIGFYQRYCDILGVSYGANLDCNNQTPFGS

>Pb.isotig19861

METKQVNRILVVAGICLMMSSWFCCIEAAAAAAAADENEETKFKKILCAKATDCKNKTISELFTVDQFESLFSHRNAPLAHAQGFWDYHSFITAAAHYEPKGFGTTGGDIVQKRELAAFFAHIATETSCESLMAQAASTAPSDSPTKWGLCYKEELSPDSTYCESSLVYPCAPGVSYHGRGALPVYWNYNYGQLGQALKVDLLHHAEYLSENATLAFAAAIWRWMTPMKVKQPSAHQVMVGKWVPTKNDTEALRLPGFGMTINILKADAECGTDSDDKQMNTRIAHYLDFLDHMDVGRENAGDNVDCSEQKVLNPSSSAST

>Pb.isotig19666

MAIVITGLPSVSADDCGKNAGGALCPGGLCCSKYGYCGNTQAHCGKDCQSQCGGGGSTPTPTPPTPTPTTPTPTPSGQGVASIITEDLFNQLLKYKDDSRCKANGFYSYDAFINVANAYPGFGTAGDVTSNKRELAAFFGQTSHETTGGWPAAPDGPYAWGYCLKEEIGTPPPVYCNETAQWPCGSGKSYYGRGPIQISWNYNYGPAGKAIGFDGINNPDIVASDATVSFKTAIWFWMTAQSPKPSCHDVMTGRWSPSSSDSAAGRAPGYGAVTNIINGGLECGKGSYPKQEDRIGFYKRYCDILGVSYGSNLDCNNQTPFGS

>Pb.isotig19621

MTTSLVYSSSSQSASHGGGVKAAYWPSYTVSYSPPSSINAALFTHIFYAFANLNDQTFQVELPATAKPAEFTLTLRKKNPFLKTLISIGGGGSNSTAFALMASNAFRRKVFINSAITLARKYGFHGLDLDWEFPKDATEMRNLGSLFAEWRHAIELEARAWPRRPRLLLTAAVYFAQYFLLAADKRAYPATSITQNLDWVNVMCFDYHGSWDTSATGAHAALYDPSSNISNSFGIGSWLHSGVPTNKVVMGMPLYGRSWILKSLDETGIGAPAIAAGPKQTLSSETGVMFFSEIIDFISQKNATEVFDKETVSAYAYSSDMMWVGYDNKQSVAAKVSFAKNKHLLGYFFWAISQDSNWMLSAQASVSWNQAR

>Pb.isotig19602

MAMGRICLVVLLALALCLNVSLASKKMKICDKGWECKGAYCCNQTISEIFTVDNFEELFSKRNTPVAHAVGFWDYYSFINAAAQFEGIGFGTTGGQVMQQKELAAFLGNVAAETSCGYNVATGGPTAWGLCYKEEMSPDQLYCDQNLLYPCAPGASYHGRGALPIYWNFNYGPIGEALKLDLLTSPDMVSNNATIGFLTAMWRWMNPIKPKQPSAHDVFVGNWKPTKNDTESYRLPGFGMVINVLNGGLECGKGDIDAMNNRISHYLYFLDLLGVGREQAGDNLDCGQQVPLNPISTSTTSR

>Pb.isotig11638

MTLSAIILWLVLAFDLVSMCRGDVGDFATQDFFNGILSGASDSCAGKTFYTYNNFMDAATAFSGFGTTGADVDHKREIAAFFANIAHETSRLCYVEQIEKSDYCDSTNKKYQCVAGKQYYGRGPLQLTWNYNYDAAGDYLGFDGLNHPEIVAQNGSISWKTAVWFWMKHSNCHSAITSGQGFRATIKAISGDECNGGDSDAIDERVNYYTNYCNEFGVDPGSNLSC

>Pb.isotig11637

MTLSAIILWLVLAFDLVSMCRGDVGDFATQDFFNGILSGASDSCTGKTFYTYNNFMDAATAFSGFGTTGPDVDHKREIAAFFANVAHETSRLCYVEQIEKSDYCDSTNQKYQCVAGKQYYGRGPLQLTWNYNYGAAGDYLGFDGLNHPEIVAQNGSISWKTAVWFWMKHSNCHSAITSGQGFMATIKAISGDECNGGDSNAVDERVNYYTNYCNEFGVDPGNNLSC

>Pb.isotig11200

MAAQLPVSAIILWFVFALSALSICRGAVSDIATEDFFNGILSAATDGCAGKTFYTYSDFINAANSFSSFGTTGTSDDNKREIAAFFGNVAHETTNLCFVEENAKSDNCDSSNTQYPCASGQQYYGRGPLQLTGNGNYGAAGSYLGVDLLNNPGLVAQDDLTSWKTALWFWNVNSNCHTAITSGQGFGATIQAINGAKECNGGNTAEVNDRVSRYTTYCSQLVVDPGSNLSC

>Pb.isotig08316

MAQAPKNLCIILFFITSSIIYSSSSSNNTTSNNGLKAAYWPSDSSLSPSSISTDLFTHIFYAFADLNDQTFQVQLPATADPAEFTSTLLQKNPSLKTLISIGGGGSNATAFALMASNASHRKVFINSAIAVARKYGFHGLDLDWEFPQDAAEMEYLGTLFDDWRHAIDLEATASGRPRLLLTAAVYFAQYFFVWGKKRAYPVTSIARNLDWVNVMCYDYHGSWDISATGAHAALYDPTSNISTSFGIGSWLHSGVPPNKVAMGMPLYGRSWILKSLDETEIGAPAVAAGPKQTLSNEKGVMFFSEIRELINQKNATEVFDKETVSAYSYSSDLLWVGYDNQESVATKVSFAKEMHLLGYFFWAIGQDNNWMLSAQASDSWN

>Pb.isotig08315

MAQAPKNLCIILFFITSSIIYSSSSSNNTTSNNGVKAAYWPSDSSLSPSSISTDLFTHIFYAFADLNDQTFQVQLPATADPAEFTSTLLQKNPSLKTLISIGGGGSNATAFALMASNTSYRKVFINSTIALARKYGFHGLDLDWEFPQDAAEMEYLGTLFDDWRHDIDLEATASGRPRLLLTAAVYFAQYFFVWGEKRAYPVTSIARNLDWVNVMCYDYHGSWDISATGAHAALYDPTSNISTSFGIGSWLHSGVPPNKVAMGMPLYGRSWILKSLDETEIGAPAVAAGPKQTLSNEKGVMFFSEIRELINQKNATEVFDKETVSAYSYSSDLLWVGYDNQESVATKVSFAKEMHLLGYFFWAIGQDNNWMLSAQASDSWN

>Pb.isotig06375

MATVLVLVQLLLGVIVNVVNAESVANIVSRSFFDSILNAPTLANCSCAIGLCCSKWGYCGNTASYCGDGCQSGSCYNQSGGSSTGRHGGAGAIISSSFFDGLAGGAASSCEGKGFYTYKAFVAASSAYSVFGTTGSSDDQKRELAAFFANVMHETGGLCYINEVNPGSIYCDSSSNTWPCASGKSYHGRGPLQLSWNYNYGAAGEDIGFDGLNHPEEVALDAIISFRTGVWFWMKNSNCHSAITSGQGFGATIKAINSMECNGGNTAAVNSRINYYTKFCSELGVDPGTKLSC

>Pb.isotig02119

MKSMKFSAMAIALLTMATMNLYFVSAEQCGQQAGGALCPGGLCCSKWGWCGNTDAHCGQDCQSQCGGSTPTPGGQGVASIITESIFNELLKHRNDAGCKASGFYTYSAFIAAANAFPSFGTTGDVATRKRELAAFFGQTSHETTGGWATAPDGAYAWGYCFKEEQGNPPAEYCQATSQWPCASGKRYYGRGPVQLSWNYNYGPAGKAIGFDGINNPDIVASDATVSFKTAIWFWMTAQSPKPSCHDVMTGKWTPSGSDSAAGRAAGYGAVTNIINGGLECGKGSDSRQQDRIGFYKRYSDILGVSYGSNLDCNNQRPFGAAVQSEPRIIKTVV

>Pb.isotig02112

MKSMKFSAMAIALLTMATMNLYFVSAEQCGQQAGGALCPGGLCCSKWGWCGNTDAHCGQDCQSQCGGSTPTPGGQGVASIITESIFNELLKHRNDAGCKASGFYTYSAFIAAANAFPSFGTTGDVATRKRELAAFFGQTSHETTGGWATAPDGAYAWGYCFKEEQGNPPAEYCQATSQWPCASGKRYYGRGPVQLSWNYNYGPAGKAIGFDGINNPDIVASDATVSFKTAIWFWMTAQSPKPSCHDVMTGKWTPSGSDSAAGRATGYGAVTNIINGGLECGKGSDSRQQDRIGFYKRYSDILGVSYGSNLDCNNQRPFGAAVQSEPRLIKTVV

>Pb.isotig02108

MKSMKFSAMAIALLTMATMNLYFVSAEQCGQQAGGALCPGGLCCSKWGWCGNTDAHCGQDCQSQCGGSTPTPGGQGVASIITESIFNELLKHRNDAGCKASGFYTYSAFIAAANAFPSFGTTGDVATRKRELAAFFGQTSHETTGGWATAPDGAYAWGYCFKEEQGNPPAEYCQATSQWPCASGKRYYGRGPVQLSWNYNYGPAGKAIGFDGINNPDIVASDATVSFKTAIWFWMTAQSPKPSCHDVMTGKWTPSGSDSAAGRAAGYGAVTNIINGGLECGKGSDSRQQDRIGFYKRYSDILGVSYGSNLDCNNQRPFGAAVQSEPRLIKTVV

>Pb.contig9822

MATHLRSVLILWLALALYVVSICRGDVGDIVTRDFFNGILSGAADSCAGKNFYTYDDFITAANAFSGFGTTGTSDDNKRELAAFFANVAHETGSLCYIEEINKSDYCDSTNTQYPCVAGKQYYGRGPLQLTWNYNYGAAGNYLGFDGLNNPDIVAQDDSISWKTAVWFWMLDSNCHSAITSGQGFGATIQAINSGECNGGNTAAVTDRVNYYNNYCGQFGVDPGSNVSC

>Pb.contig8129

MTPSVIILLLVLAFDVVSICRGDVGDFATQDFFNGILSGASDSCAGKTFYTYNNFMDAATAFSGFGTTGADVDHKREIAAFFANIARETSRLCYVEQIEKSDYCDSTNKKYQCVAGKQYYGRGPLQLTWNYNYDAAGDYLGFDGLNHPEIVAQNGSISWKAAVWFWMKHSNCHSAITSGQGFRATIKAISGDECNGGDSDSVDERVNYYTNYCNEFGVDPGSNLSC

>Pb.contig7310

MLFPSAEEMLGILPPKISSMEFCLVLLIVAQERPSIHTITSWTQPLHSLALAQQAPTLTTREIAAFFANIAHETSRLCYVEQIEKSDYCDSTNKKYQCVAGKQYYGRGPLQLTWNYNYDAAGHYLGFDGLNHPEIVAQNGSISWKTAVWFWMKHSNCHSAITSGQGFRATIKAISGDECNGGDSDSVDERVNYYTNYCNEFGVDPGSNLSC

>Pb.contig7077

MGTRTGRSVIVSPYEHMVLRMSMVLLLVVVVSVNVVYAQNCGCASGLCCSKWGYCGTTSAYCGNGCQSGPCYGSGGGSPSGGGGNVGTIISQNFFSGLANAAGSSCEGKGFYTYNAFITAANAYSGFGTTGASDNQKRELAAFFANVMHETGGLCYINEINPLSNFCQSSSTWPCASGKSYHGRGPIQLTWNYNYGAAGQDIGFDGLNNPEKVGQDATISFKTAVWFWMKNSNCHSAITSGQGFGATIKAINGGECNGGHSAQVNSRVNYYKKLCSQLGVDPGTNISC

>Pb.contig4532

MGSSSGNSAMVVVVLVLLLVGVSVTAQNCGCASDLCCSKYGYCGTTSAYCGDGCQSGPCTSSGGSLSGGGGSVGTIISQSFFDGLKGGAASSCEGKGFYTYNAFIAAANAYSGFGTTGSADVTKRELAAFFANVMHETGGMCYINERNPPMNYCMGSATSPCASGKSYYGRGPLQLSWNYNYGAAGQSIGFDGVNNPEKVGQDSTISFKTAVWFWMKNSNCHSAITSGQGFGGTIKAINSQECNGGNSGEVNSRVNYYKNICSQLGVDPGANVSC

>Pb.contig3795

MGTRTGRSVIASPYEHMVLRMSMVLLLVVVVSVNVVYAQNCGCASGLCCSKWGYCGTTSAYCGNGCQSGPCYGSGGGSPSGGGGNVGTIISQNFFSGLANAAGSSCEGKGFYTYNAFITAANAYSGFGTTGASDNQKRELAAFFANVMHETGGLCYINEINPLSNFCQSSSTWPCASGKSYHGRGPIQLTWNYNYGAAGQDIGFDGLNNPEKVGQDATISFKTAVWFWMKNSNCHSAITSGQGFGGTIKAINSRECNGGNSAQVNSRVNYYKKLCSQLGVDPGTNISC

>Pb.contig3728

MATFLRSTLILWLALALYVVSFCQGDVGDIVTQDFFNAILSGADGSCAGQNFYTYNDFINAANAFSGFGTTGTSDDNKRELAAFFANVAHETGSLCYIEEISKSDYCDSTNTQYPCATGKQYYGRGPIQLTWNYNYGAAGNYVGFDGLNNPDIVAQDDSISWKTAVWFWMLDSNCHSAITSGQGFGATIQAINSGECNGGNTAAVTSRVNYYNNYCSQFGVDPGSDVSC

>Pb.contig3417

MAAQLPVSAIILWFVFALSALSICRGAVSDIATEDFFNGILSAATDGCAGKTFYTYSDFINAANSFSSFGTTGTSDDNKREIAAFFGNVAHETTNLCFVEENAKSDNCDSSNTQYPCASGQQYYGRGPLQLTGNGNYGAAGSYLGVDLLNNPGLVAQDDLTSWKTALWFWNVNSNCHTAITSGQGFGATIQAINGAKECNGVNTAEVNDRVSRYTTYCSQLVVDPGSNLSC

>PcTPS-LAS1 synthase-like

RAVYTKTSNFTVILDDLYDAHGTLDNLKLFSESVKRWDLSLVDQMPQDMKICFKGFYNTFNEIAEEGRKRQGRDVLGYIQKVWEVQLEAYTKEAEWSAVRYVPSYDEYIGNASVSIALGTVVLISALFTGEILTDDILSKIGRDSRFLYLMGLTGRLVNDTKTYQAERGQGEVASAVQCYMKDHPEISEEEALKHVYTIMDNALDELNREFVNNRDVPDTCRRLVFETARIMQLFYMDGDGLTLSHNMEIKEHVKNCLFQPVA

>Pc(E)-beta-farnesene synthase-like

MLSLYRASQLAFPGETILDEARDFATKYLREALEKSEIFTAWNNKHNLSQEIQYELENSWHASVSRVEAKRYCQGYSSDYARLAKSVHKLPRVSQNILELAKLDFNIIQAIHQKEMKNVTTWFKHSEFPLLPFGRERPVECFFIVAAGTYEPQYAKCRFLFSKVACLNTVLDDMYDTYGTLELKLFTEAVRRWDLSLTESLPDYMKLCYKIFYEIVHEVVREAEKLQGRELLSFFRKGWEEYLLGYYEEAEWLASEYVPSLEEYIRNGIISIGQRILLVSGVLLMEGQILSQEALEELDYPGRRVLTELNCIITRLADDIHTYKAEKARGELASSIECYMKEHPGSTEEVAVNYMYSLLEPAVKELTWEFLKPHDTADVDIPFQCKKMLMEETRVTMVIFKEGDGFGIFKTKIKDYIKECLIEPLPL

>PcTPS-3car1 synthase-like

MVDNVERLGIDRHFQTEIKVALDYVYRYWSEEGIGCGRDSAFTDLNTTALAFRIFRLHGYTVSSDVFEHFKDQKGQFAASANDTELQTRSVFNLFRASLIAFPEEKVLEEAEKFAAAYLKAALQTLPVSGLSREIQYVFDYRWHSNLPRLEARSYIDILADNTISGTPDANTKKLLELAKLEFNIFHSVQQKELQCLWRWWKEWGCPELTFIRHRYVEFYTLVSGIDMVPEHATFRLSCVKTCHLITILDDMYDTFGTIDELRLFTAAVKRWDPSATECLPEYMKGVYMVLYETVNEMAKEAQKSQGRDTLGYVRQALEDYIGSYLKEAEWIATGYVPTFQEYFENGKLSSGHRIATLQPILTLSIPFPHHILQEIDFPSKFNDYAASILRLRGDTRCYKADSARGEEASCISCYMRDNPGSTQEDALNHINGMIEDMIKKLNWEFLRPDNNAPISSKKHAFNISRGLHHFYNYRDGYSVASKETKDLVIKTVLEPVLM

>PcTPS-(+)alpha-pin1 synthase-like

GTLEPMLTLGFPLPPRILQEIDFPSKFNDLTCAILRLKGDTQCYKADRARGEEASAVSCYMKDHPGITEEDAVNQVNAMVDNLTKELNWELLRPDSGVPISYKKVAFDICRVFHYGYKYRDGFSVASVEIKNLVTRTVVETVPL

>Ag beta-phellandrene synthase gi|7381249

MALVSSAPKSCLHKSLIRSTHHELKPLRRTIPTLGMCRRGKSFTPSVSMSLTTAVSDDGLQRRIGDYHSN

LWDDDFIQSLSTPYGEPSYRERAEKLIGEVKEMFNSMPSEDGESMSPLNDLIERLWMVDSVERLGIDRHF

KKEIKSALDYVYSYWNEKGIGCGRDSVFPDVNSTASGFRTLRLHGYSVSSEVLKVFQDQNGQFAFSPSTK

ERDIRTVLNLYRASFIAFPGEKVMEEAEIFSSRYLKEAVQKIPVSSLSQEIDYTLEYGWHTNMPRLETRN

YLDVFGHPTSPWLKKKRTQYLDSEKLLELAKLEFNIFHSLQQKELQYLSRWWIHSGLPELTFGRHRHVEY

YTLSSCIATEPKHSAFRLGFAKTCHLITVLDDIYDTFGTMDEIELFNEAVRRWNPSEKERLPEYMKEIYM

ALYEALTDMAREAEKTQGRDTLNYARKAWEVYLDSYTQEAKWIASGYLPTFEEYLENAKVSSGHRAAALT

PLLTLDVPLPDDVLKGIDFPSRFNDLASSFLRLRGDTRCYKADRDRGEEASSISCYMKDNPGLTEEDALN

HINAMINDIIKELNWELLKPDSNIPMTARKHAYEITRAFHQLYKYRDGFSVATQETKSLVRRTVLEPVPL

>Ag myrcene synthase gi|2411481

MALVSISPLASKSCLRKSLISSIHEHKPPYRTIPNLGMRRRGKSVTPSMSISLATAAPDDGVQRRIGDYH

SNIWDDDFIQSLSTPYGEPSYQERAERLIVEVKKIFNSMYLDDGRLMSSFNDLMQRLWIVDSVERLGIAR

HFKNEITSALDYVFRYWEENGIGCGRDSIVTDLNSTALGFRTLRLHGYTVSPEVLKAFQDQNGQFVCSPG

QTEGEIRSVLNLYRASLIAFPGEKVMEEAEIFSTRYLKEALQKIPVSALSQEIKFVMEYGWHTNLPRLEA

RNYIDTLEKDTSAWLNKNAGKKLLELAKLEFNIFNSLQQKELQYLLRWWKESDLPKLTFARHRHVEFYTL

ASCIAIDPKHSAFRLGFAKMCHLVTVLDDIYDTFGTIDELELFTSAIKRWNSSEIEHLPEYMKCVYMVVF

ETVNELTREAEKTQGRNTLNYVRKAWEAYFDSYMEEAKWISNGYLPMFEEYHENGKVSSAYRVATLQPIL

TLNAWLPDYILKGIDFPSRFNDLASSFLRLRGDTRCYKADRDRGEEASCISCYMKDNPGSTEEDALNHIN

AMVNDIIKELNWELLRSNDNIPMLAKKHAFDITRALHHLYIYRDGFSVANKETKKLVMETLLESMLF

>Ag (-)limonene/(-)alpha-pinene synthase gi|7381253

MALLSIVSLQVPKSCGLKSLISSSNVQKALCISTAVPTLRMRRRQKALVINMKLTTVSHRDDNGGGVLQR

RIADHHPNLWEDDFIQSLSSPYGGSSYSERAVTVVEEVKEMFNSIPNNRELFGSQNDLLTRLWMVDSIER

LGIDRHFQNEIRVALDYVYSYWKEKEGIGCGRDSTFPDLNSTALALRTLRLHGYNVSSDVLEYFKDQKGH

FACPAILTEGQITRSVLNLYRASLVAFPGEKVMEEAEIFSASYLKEVLQKIPVSSFSREIEYVLEYGWHT

NLPRLEARNYIDVYGQDSYESSNEMPYVNTQKLLKLAKLEFNIFHSLQQKELQYISRWWKDSCSSHLTFT

RHRHVEYYTMASCISMEPKHSAFRLGFVKTCHLLTVLDDMYDTFGTLDELQLFTTAFKRWDLSETKCLPE

YMKAVYMDLYQCLNELAQEAEKTQGRDTLNYIRNAYESHFDSFMHEAKWISSGYLPTFEEYLKNGKVSSG

SRTATLQPILTLDVPLPNYILQEIDYPSRFNDLASSLLRLRGDTRCYKADRARGEEASAISCYMKDHPGS

TEEDALNHINVMISDAIRELNWELLRPDSKSPISSKKHAFDITRAFHHLYKYRDGYTVASSETKNLVMKT

VLEPVAL

>Ag terpinolene synthase gi|7381251

MALVSILPLSSKSVLHKSWIVSTYEHKAISRTIPNLGLRGRGKSVTHSLRMSLSTAVSDDHGVQRRIVEF

HSNLWDDDFIQSLSTPYGAPSYRERADRLIVEVKGIFTSISAEDGELITPLNDLIQRLLMVDNVERLGID

RHFKNEIKAALDYVYSYWNEKGIGSGSDSGVADLNSTALGFRILRLHGYSVSSDVLEHFKEEKEKGQFVC

SAIQTEEEIKSVLNLFRASLIAFPGEKVMEEAEIFSKIYLKEALQNIAVSSLSREIEYVLEDGWQTNMPR

LETRNYIDVLGENDRDETLYMNMEKLLEIAKLEFNIFHSLQQRELKDLSRWWKDSGFSHLTFSRHRHVEF

YALASCIETDRKHSGFRLGFAKMCHLITVLDDIYDTFGTMEELELFTAAFKRWDPSATDLLPEYMKGLYM

VVYETVNEIAREADKSQGRETLNDARRAWEAYLDSYMKEAEWISSGYLPTFEEYMETSKVSFGYRIFALQ

PILTMDVPLTHHILQEIDFPLRFNDLICSILRLKNDTRCYKADRARGEEASCISCYMKENPGSTEEDAIN

HINAMVNNLIKEVNWELLRQDGTAHIACKKHAFDILKGSLHGYKYRDGFSVANKETKNWVRRTVLESVPL

>Ag (-)4S-limonene synthase gi|2429145

MALLSIVSLQVPKSCGLKSLISSSNVQKALCISTAVPTLRMRRRQKALVINMKLTTVSHRDDNGGGVLQR

RIADHHPNLWEDDFIQSLSSPYGGSSYSERAETVVEEVKEMFNSIPNNRELFGSQNDLLTRLWMVDSIER

LGIDRHFQNEIRVALDYVYSYWKEKEGIGCGRDSTFPDLNSTALALRTLRLHGYNVSSDVLEYFKDEKGH

FACPAILTEGQITRSVLNLYRASLVAFPGEKVMEEAEIFSASYLKKVLQKIPVSNLSGEIEYVLEYGWHT

NLPRLEARNYIEVYEQSGYESLNEMPYMNMKKLLQLAKLEFNIFHSLQLRELQSISRWWKESGSSQLTFT

RHRHVEYYTMASCISMLPKHSAFRMEFVKVCHLVTVLDDIYDTFGTMNELQLFTDAIKRWDLSTTRWLPE

YMKGVYMDLYQCINEMVEEAEKTQGRDMLNYIQNAWEALFDTFMQEAKWISSSYLPTFEEYLKNAKVSSG

SRIATLQPILTLDVPLPDYILQEIDYPSRFNELASSILRLRGDTRCYKADRARGEEASAISCYMKDHPGS

IEEDALNHINAMISDAIRELNWELLRPDSKSPISSKKHAFDITRAFHHVYKYRDGYTVSNNETKNLVMKT

VLEPLAL

>Ag pinene synthase gi|2411483

MALVSTAPLASKSCLHKSLISSTHELKALSRTIPALGMSRRGKSITPSISMSSTTVVTDDGVRRRMGDFH

SNLWDDDVIQSLPTAYEEKSYLERAEKLIGEVKNMFNSMSLEDGELMSPLNDLIQRLWIVDSLERLGIHR

HFKDEIKSALDYVYSYWGENGIGCGRESVVTDLNSTALGLRTLRLHGYPVSSDVFKAFKGQNGQFSCSEN

IQTDEEIRGVLNLFRASLIAFPGEKIMDEAEIFSTKYLKEALQKIPVSSLSREIGDVLEYGWHTYLPRLE

ARNYIQVFGQDTENTKSYVKSKKLLELAKLEFNIFQSLQKRELESLVRWWKESGFPEMTFCRHRHVEYYT

LASCIAFEPQHSGFRLGFAKTCHLITVLDDMYDTFGTVDELELFTATMKRWDPSSIDCLPEYMKGVYIAV

YDTVNEMAREAEEAQGRDTLTYAREAWEAYIDSYMQEARWIATGYLPSFDEYYENGKVSCGHRISALQPI

LTMDIPFPDHILKEVDFPSKLNDLACAILRLRGDTRCYKADRARGEEASSISCYMKDNPGVSEEDALDHI

NAMISDVIKGLNWELLKPDINVPISAKKHAFDIARAFHYGYKYRDGYSVANVETKSLVTRTLLESVPL

>Ag (-)camphene synthase gi|2411485

MALLSITPLVSRSCLSSSHEIKALRRTIPTLGICRPGKSVAHSINMCLTSVASTDSVQRRVGNYHSNLWD

DDFIQSLISTPYGAPDYRERADRLIGEVKDIMFNFKSLEDGGNDLLQRLLLVDDVERLGIDRHFKKEIKT

ALDYVNSYWNEKGIGCGRESVVTDLNSTALGLRTLRLHGYTVSSDVLNVFKDKNGQFSSTANIQIEGEIR

GVLNLFRASLVAFPGEKVMDEAETFSTKYLREALQKIPASSILSLEIRDVLEYGWHTNLPRLEARNYMDV

FGQHTKNKNAAEKLLELAKLEFNIFHSLQERELKHVSRWWKDSGSPEMTFCRHRHVEYYALASCIAFEPQ

HSGFRLGFTKMSHLITVLDDMYDVFGTVDELELFTATIKRWDPSAMECLPEYMKGVYMMVYHTVNEMARV

AEKAQGRDTLNYARQAWEACFDSYMQEAKWIATGYLPTFEEYLENGKVSSAHRPCALQPILTLDIPFPDH

ILKEVDFPSKLNDLICIILRLRGDTRCYKADRARGEEASSISCYMKDNPGLTEEDALNHINFMIRDAIRE

LNWELLKPDNSVPITSKKHAFDISRVWHHGYRYRDGYSFANVETKSLVMRTVIEPVPL

>Ag d-selinene synthase gi|2961460

MAEISESSIPRRTGNHHGNVWDDDLIHSLNSPYGAPAYYELLQKLIQEIKHLLLTEMEMDDGDHDLIKRL

QIVDTLECLGIDRHFEHEIQTAALDYVYRWWNEKGIGEGSRDSFSKDLNATALGFRALRLHRYNVSSGVL

KNFKDENGKFFCNFTGEEGRGDKQVRSMLSLLRASEISFPGEKVMEEAKAFTREYLNQVLAGHGDVTDVD

QSLLREVKYALEFPWHCSVPRWEARSFLEIYGHNHSWLKSNINQKMLKLAKLDFNILQCKHHKEIQFITR

WWRDSGISQLNFYRKRHVEYYSWVVMCIFEPEFSESRIAFAKTAILCTVLDDLYDTHATLHEIKIMTEGV

RRWDLSLTDDLPDYIKIAFQFFFNTVNELIVEIVKRQGRDMTTIVKDCWKRYIESYLQEAEWIATGHIPT

FNEYIKNGMASSGMCILNLNPLLLLDKLLPDNILEQIHSPSKILDLLELTGRIADDLKDFEDEKERGEMA

SSLQCYMKENPESTVENALNHIKGILNRSLEEFNWEFMKQDSVPMCCKKFTFNIGRGLQFIYKYRDGLYI

SDKEVKDQIFKILVHQVPMEE

>Ag gamma-humulene synthase gi|2961462

MAQISESVSPSTDLKSTESSITSNRHGNMWEDDRIQSLNSPYGAPAYQERSEKLIEEIKLLFLSDMDDSC

NDSDRDLIKRLEIVDTVECLGIDRHFQPEIKLALDYVYRCWNERGIGEGSRDSLKKDLNATALGFRALRL

HRYNVSSGVLENFRDDNGQFFCGSTVEEEGAEAYNKHVRCMLSLSRASNILFPGEKVMEEAKAFTTNYLK

KVLAGREATHVDESLLGEVKYALEFPWHCSVQRWEARSFIEIFGQIDSELKSNLSKKMLELAKLDFNILQ

CTHQKELQIISRWFADSSIASLNFYRKCYVEFYFWMAAAISEPEFSGSRVAFTKIAILMTMLDDLYDTHG

TLDQLKIFTEGVRRWDVSLVEGLPDFMKIAFEFWLKTSNELIAEAVKAQGQDMAAYIRKNAWERYLEAYL

QDAEWIATGHVPTFDEYLNNGTPNTGMCVLNLIPLLLMGEHLPIDILEQIFLPSRFHHLIELASRLVDDA

RDFQAEKDHGDLSCIECYLKDHPESTVEDALNHVNGLLGNCLLEMNWKFLKKQDSVPLSCKKYSFHVLAR

SIQFMYNQGDGFSISNKVIKDQVQKVLIVPVPI

>Ag E-alpha-bisabolene synthase gi|3252840

MAGVSAVSKVSSLVCDLSSTSGLIRRTANPHPNVWGYDLVHSLKSPYIDSSYRERAEVLVSEIKAMLNPA

ITGDGESMITPSAYDTAWVARVPAIDGSARPQFPQTVDWILKNQLKDGSWGIQSHFLLSDRLLATLSCVL

VLLKWNVGDLQVEQGIEFIKSNLELVKDETDQDSLVTDFEIIFPSLLREAQSLRLGLPYDLPYIHLLQTK

RQERLAKLSREEIYAVPSPLLYSLEGIQDIVEWERIMEVQSQDGSFLSSPASTACVFMHTGDAKCLEFLN

SVMIKFGNFVPCLYPVDLLERLLIVDNIVRLGIYRHFEKEIKEALDYVYRHWNERGIGWGRLNPIADLET

TALGFRLLRLHRYNVSPAIFDNFKDANGKFICSTGQFNKDVASMLNLYRASQLAFPGENILDEAKSFATK

YLREALEKSETSSAWNNKQNLSQEIKYALKTSWHASVPRVEAKRYCQVYRPDYARIAKCVYKLPYVNNEK

FLELGKLDFNIIQSIHQEEMKNVTSWFRDSGLPLFTFARERPLEFYFLVAAGTYEPQYAKCRFLFTKVAC

LQTVLDDMYDTYGTLDELKLFTEAVRRWDLSFTENLPDYMKLCYQIYYDIVHEVAWEAEKEQGRELVSFF

RKGWEDYLLGYYEEAEWLAAEYVPTLDEYIKNGITSIGQRILLLSGVLIMDGQLLSQEALEKVDYPGRRV

LTELNSLISRLADDTKTYKAEKARGELASSIECYMKDHPECTEEEALDHIYSILEPAVKELTREFLKPDD

VPFACKKMLFEETRVTMVIFKDGDGFGVSKLEVKDHIKECLIEPLPL

>Ag abietadiene cyclase gi|1477584

MAMPSSSLSSQIPTAAHHLTANAQSIPHFSTTLNAGSSASKRRSLYLRWGKGSNKIIACVGEGGATSVPY

QSAEKNDSLSSSTLVKREFPPGFWKDDLIDSLTSSHKVAASDEKRIETLISEIKNMFRCMGYGETNPSAY

DTAWVARIPAVDGSDNPHFPETVEWILQNQLKDGSWGEGFYFLAYDRILATLACIITLTLWRTGETQVQK

GIEFFRTQAGKMEDEADSHRPSGFEIVFPAMLKEAKILGLDLPYDLPFLKQIIEKREAKLKRIPTDVLYA

LPTTLLYSLEGLQEIVDWQKIMKLQSKDGSFLSSPASTAAVFMRTGNKKCLDFLNFVLKKFGNHVPCHYP

LDLFERLWAVDTVERLGIDRHFKEEIKEALDYVYSHWDERGIGWARENPVPDIDDTAMGLRILRLHGYNV

SSDVLKTFRDENGEFFCFLGQTQRGVTDMLNVNRCSHVSFPGETIMEEAKLCTERYLRNALENVDAFDKW

AFKKNIRGEVEYALKYPWHKSMPRLEARSYIENYGPDDVWLGKTVYMMPYISNEKYLELAKLDFNKVQSI

HQTELQDLRRWWKSSGFTDLNFTRERVTEIYFSPASFIFEPEFSKCREVYTKTSNFTVILDDLYDAHGSL

DDLKLFTESVKRWDLSLVDQMPQQMKICFVGFYNTFNDIAKEGRERQGRDVLGYIQNVWKVQLEAYTKEA

EWSEAKYVPSFNEYIENASVSIALGTVVLISALFTGEVLTDEVLSKIDRESRFLQLMGLTGRLVNDTKTY

QAERGQGEVASAIQCYMKDHPKISEEEALQHVYSVMENALEELNREFVNNKIPDIYKRLVFETARIMQLF

YMQGDGLTLSHDMEIKEHVKNCLFQPVA

>Pa Diterpene synthase gi|75115607

MALLSSSLSSQIPTGAHHLTLNAYANTQCIPHFFSTLNAGTSAGKRSSLYLRWGKGSNKIIACVGEDSLS

APTLVKREFPPGFWKDHVIDSLTSSHKVAASDEKRIETLISEIKNMFRSMGYGDTNPSAYDTAWVARIPA

VDGSEQPEFPETLEWILQNQLKDGSWGEGFYFLAYDRILATLACIITLTLWRTGEIQVQKGIEFFKTQAG

KIEDEADSHRPSGFEIVFPAMLKEAKVLGLDLPYELPFIKQIIEKREAKLERLPTNILYALPTTLLYSLE

GLQEIVDWQKIIKLQSKDGSFLSSPASTAAVFMRTGNKKCLEFLNFVLKKFGNHVPCHYPLDLFERLWAV

DTIERLGIDRHFKEEIKDALDYVYSHWDERGIGWARENPVPDIDDTAMGLRILRLHGYNVSSDVLKTFRD

ENGEFFCFLGQTQRGVTDMLNVNRCSHVAFPGETIMEEAKTCTERYLRNALEDVGAFDKWALKKNIRGEV

EYALKYPWHRSMPRLEARSYIEHYGPNDVWLGKTMYMMPYISNEKYLELAKLDFNHVQSLHQKELRDLRR

WWTSSGFTELKFTRERVTEIYFSPASFMFEPEFATCRAVYTKTSNFTVILDDLYDAHGTLDDLKLFSDSV

KKWDLSLVDRMPQDMKICFMGFYNTFNEIAEEGRKRQGRDVLGYIRNVWEIQLEAYTKEAEWSAARYVPS

FDEYIDNASVSIALGTVVLISALFTGEILTDDVLSKIGRGSRFLQLMGLTGRLVNDTKTYEAERGQGEVA

SAVQCYMKDHPEISEEEALKHVYTVMENALDELNREFVNNREVPDSCRRLVFETARIMQLFYMDGDGLTL

SHETEIKEHVKNCLFQPVA

>Ps (E)-beta farnesene synthase gi|296496002

MASASVASSTLPSGLSSSSSSSVIRRTANPHPNVWDYDFVQSLQSPYTDSCYGERAETLISEIKLVLTGE

GDALMITPSAYDTAWVARVPAIDGSSRPQFPQTVDWILKNQLKDGSWGTESHFLLSDRLLATLSCVLALL

KWKVGHVQVEHGIEFIKSNLEAIKDESNQDSCVTDFEIIFPSLIGEAQSLHLGLPYNLPYVRMLQMKRRE

KLANLPRDEIHGGTLLSSLEGIQDTVEWERIMEVQSQDGSFSGSPASTACVFMHTGDMKCLQFLNSVLTK

FGISVPFLYPVDLLEGLLMVDNIVRLGIDRHFEKEIKEILDCVYRHWNESLNPVADLEITALGFRLLRLH

RYSVTPAVFENFKDADGHFFGSTSQFNKNVASMLSLYRASQLAFPGETILDEARDFATKYLREALEKSEI

FTAWNNKQNLSQEIQYELENSWHASVSRVEAKRYCQGYSSDYARLAKSVYKLPRANNQKILELAKLDFNI

IQAIHQKEMKNVTSWFKHSEFPLLPFGRERPVECFFLVAAGTYEPQYAKCRFLFSKVACLNTVLDDMYDT

YGTLDELKLFTEAVRRWDLSLTESLPDYMKLCYKIFYEIVHEVVREAEKLQGRELLSFFRKGWEEYLLGY

YEEAEWLASEYVPSLEEYIRNGIISIGQRILLVSGVLLMEGQILSQEALEKLDYPGRRVLTELNCIITRL

ADDIHTYKAEKARGELASSIECYMKEHPGSTEEVAVNYMYSLLEPAVKELTWEFLKPDDTSDVDIPFQCK

KMLMEETRVTMVIFKEGDGFGISKTKIKDYIKECLIEPLPL

>Pa (-)limonene synthase gi|44804558

MSPVSVIPLAYKLCLPRSLMSSSREVKPLHITIPNLGMCRRGKSMAPASTSMILTAAVSDDDRVQRRRGN

YHSNLWDDDFIQSLSTPYGEPSYRERAERLKGEIKKMFRSMSKDDGELITPLNDLIQRLWMVDSVQRLGI

DRHFKNEIKSALDYVYSYWNEKGIGCGRDSVVADLNSTALGFRTLRLHGYNVSSEVLKVFEDQNGQFACS

PSKTEGEIRSALNLYRASLIAFPGEKVMDDAEIFSSRYLKEAVQEIPDCSLSQEIAYALEYGWHTNMPRL

EARNYMDVFGHPSSPWLKKNKTQYMDGEKLLELAKLEFNIFHSLQQEELQYISRWWKDSGLPKLAFSRHR

HVEYYTLGSCIATDPKHRAFRLGFVKTCHLNTVLDDIYDTFGTMDEIELFTEAVRRWDPSETESLPDYMK

GVYMVLYEALTEMAQEAQKTQGRDTLNYARKAWEIYLDSYIQEAKWIASGYLPTFQEYFENGKISSAYRA

AALTPILTLDVPLPEYILKGIDFPSRFNDLASSFLRLRGDTRCYKADRARGEEASCISCYMKDNPGSTEE

DALNHINSMINEIIKELNWELLRPDSNIPMPARKHAFDITRALHHLYKYRDGFSVATKETKSLVSRMVLE

PVPL

>Pa (-)linalool synthase gi|44804547

MALLSIAPLTSTWCVDKSLVGSSEAKALLRKIPTLEMCRLTKSVTPSISMCLTTTVSDDGVQRRIADHHP

NLWDDNFIQSLSTPYGATAYHERAQKLIGEVKVIINSILVEDGELITPPNDLLQRLSIVDSIERLGIDRH

FKNEIKSALDYVYSYWSEKGIGCGRDSVVNDLNTTALGLRTLRLHGYPVSSDVLEQFKDQNGQFACSAIQ

TEGEIKTVLNLFRASLIAFPGEKVMEEAEIFSTIYLKEALLKIPVCSLSREIAYVLEYGWHMNLPRLEAR

NYIDVFGQDPIYLRSTQKLIELAKLEFNIFQSLQQEELKHVSRWWKDSGFSQMAFARHRHVEYYTLASCI

DIYPQHSSFRLGFAKIAHLGTVLDDIYDTFGTMDELELFTAAVKRWHPSAAEGLPEYMKGVYMMFYETVN

EMAREAEKSQGRDTLNYARQALEAYIDSYMKEAKWISSGFLPTFEEYLDNGKVSFGYRIATLQPILTLGI

PFPHHILQEIDFPSRLNDLAGSILRLKGDIHSYQAERSRGEESSCISCYMKDNPEATEEDAVTYINAMVN

RLLKELNWELLKPDNNVPITSKKHAFDILRAFYHLYKDRDGFSVARNEIRNLVMTTVIEHVPL

>Pa (-)alpha/beta-pinene synthase gi|44804535

PRAAGKSCLHKSLSSSAHELKTICRTIPTLGMSRRGKSATPSMSMSLTTTVSDDGVQRRMGDFHSNLWND

DFIQSLSTSYGEPSYRERAERLIGEVKKMFNSMSSEDGELISPHNDLIQRVWMVDSVERLGIERHFKNEI

KSALDYVYSYWSEKGIGCGRESVVADLNSTALGLRTLRLHGYAVSADVLNLFKDQNGQFACSPSQTEEEI

RSVLNLYRASLIAFPGEKVMEEAEIFSAKYLEEALQKISVSSLSQEIRDVLEYGWHTYLPRMEARNHIDV

FGQDTQNSKSCINTDKLLELAKLEFNIFHSLQKRELEYLVRWWKDSGSPQMTFGRHRHIEYYTLASCIAF

EPQHSGFRLGFAKTCHIITILDDMYDTFGTVDELELFTAAMKRWDPSAADCLPEYMKVMYMIVYDTVNEM

CQEAEKAQGRDTLDYARQAWEDYLDSYMQEAKWIATGYLPTFEEYYENGKVSSGHRVAALQPILTMDIPF

PPHILKEVDFPSKLSDLACAILRLRGDTRCYKADRARGEEASSISCYMKDNPGATEEDALDHINAMISDV

IRGLNWELLKPNSSVPISSKKHVFDISRAFHYGYKYRDGYSVANIETKSLVKRTVIDPVTL

>Pa myrcene synthase gi|44804588 MSPVSVVPLACKLCLCRSMTSSTDELKPLPTTIPTRGMCGRRMSVTPSMSMSLNTVVSDNDAVQRRIGDY

HSNLWNDDFIQSLTTPYGAPSYIERADRLISEVKEMFNRMCMEDGELMSPLNDLIQRLWTVDSVERLGID

RHFKNEIKASLDYVYSYWNEKGIGCGRQSVVTDLNSTALGLRILRQHGYTVSSEVLKVFEEENGQFACSP

SQTEGEIRSFLNLYRASLIAFPGEKVMEEAQIFSSRYLKEAVQKIPVSGLSREIGDVLEYGWHTNLPRWE

ARNYMDVFGQDTNTSFNKNKMQYMNTEKILQLVKLEFNIFHSLQQRELQCLLRWWKESGLPQLTFARHRH

VEFYTLASCIACEPKHSAFRLGFAKMCHLVTVLDDVYDTFGKMDELELFTAAVKRWDLSETERLPEYMKG

LYVVVFETVNELAQEAEKTQGRNTLNYVRKAWEAYFDSYMKEAEWISTGYLPTFEEYCENGKVSSAYRVA

ALQPILTLDVQLPDDILKGIDFPSRFNDLASSFLRLRGDTRCYEADRARGEEASCISCYMKDNPGSTEED

ALNHINAMINDIIRELNWEFLKPDSNIPMPARKHAFDITRALHHLYIYRDGFSVANKETKNLVEKTLLES

MLF

>Pa E,E-alpha-farnesene synthase gi|44804601

MDLAVEIAMDLAVDDVERRVGDYHSNLWDDDFIQSLSTPYGASSYRERAERLVGEVKEMFTSISIEDGEL

TSDLLQRLWMVDNVERLGISRHFENEIKAAIDYVYSYWSDKGIVRGRDSAVPDLNSIALGFRTLRLHGYT

VSSDVFKVFQDRKGEFACSAIPTEGDIKGVLNLLRASYIAFPGEKVMEKAQTFAATYLKEALQKIQVSSL

SREIEYVLEYGWLTNFPRLEARNYIDVFGEEICPYFKKPCIMVDKLLELAKLEFNLFHSLQQTELKHVSR

WWKDSGFSQLTFTRHRHVEFYTLASCIAIEPKHSAFRLGFAKVCYLGIVLDDIYDTFGKMKELELFTAAI

KRWDPSTTECLPEYMKGVYMAFYNCVNELALQAEKTQGRDMLNYARKAWEALFDAFLEEAKWISSGYLPT

FEEYLENGKVSFGYRAATLQPILTLDIPLPLHILQQIDFPSRFNDLASSILRLRGDICGYQAERSRGEEA

SSISCYMKDNPGSTEEDALSHINAMISDNINELNWELLKPNSNVPISSKKHAFDILRAFYHLYKYRDGFS

IAKIETKNLVMRTVLEPVPM

>Pa longifolene synthase gi|44804575

MAQISKCSSLSAELNESSIISHHHGNLWDDDFIQSLKSSNGAPQYHERAAKLVEEIKNLVVSEMKDCNDD

LIRRLQMVDIFECLGIDRHFQHEIQVALDYVYRYWNQLEGIGIGSRDSLIKDFNATALGFRALRLHRYNV

SSDVLENFKNENGQFFCSSTVEEKEVRCMLTLFRASEISFPGEKVMDEAKAFTTEYLTKVLTGVDVTDVN

QSLLREVKYALEFPWHCSLPRWEARSFIEICGQNDSWLKSIMNKRVLELAKLDFNILQWAHHRELQLLSS

WWSQSDIAQQNFYRKRHVEFYLWVVIGTFEPEFSTCRITFAKISTLMTILDDLYDTHGTLEQLKIFTEGV

KRWDLSLVDRLPDYIKITFEFFLNTSNELIAEVAKTQERDMSAYIRKTWERYLEAYLQEAEWIAARHVPT

FDEYMKNGISSSGMCILNLYSLLLMGQLLPDDVLEQIHSPSKIHELVELTARLVDDSKDFETKKVGGELA

SGIECYVKDNPECTLEDASNHLNGLLDLTVKELNWEFVRHDSVALCFKKFAFNVARGLRLIYKYRDGFDV

SNQEMKTHIFKILIDPLT

>Pa E-alpha-bisabolene synthase gi|44804486

MTSVSVESGTVSCLSSNNLIRRTANPHPNIWGYDFVHSLKSPYTHDSSYRERAETLISEIKVMLGGGELM

MTPSAYDTAWVARVPSIDGSACPQFPQTVEWILKNQLKDGSWGTESHFLLSDRLLATLSCVLALLKWKVA

DVQVEQGIEFIKRNLQAIKDERDQDSLVTDFEIIFPSLLKEAQSLNLGLPYDLPYIRLLQTKRQERLANL

SMDKIHGGTLLSSLEGIQDIVEWETIMDVQSQDGSFLSSPASTACVFMHTGDMKCLDFLNNVLTKFGSSV

PCLYPVDLLERLLIVDNVERLGIDRHFEKEIKEALDYVYRHWNDRGIGWGRLSPIADLETTALGFRLLRL

HRYNVSPVVLDNFKDADGEFFCSTGQFNKDVASMLSLYRASQLAFPEESILDEAKSFSTQYLREALEKSE

TFSSWNHRQSLSEEIKYALKTSWHASVPRVEAKRYCQVYRQDYAHLAKSVYKLPKVNNEKILELAKLDFN

IIQSIHQKEMKNVTSWFRDSGLPLFTFARERPLEFYFLIAGGTYEPQYAKCRFLFTKVACLQTVLDDMYD

TYGTPSELKLFTEAVRRWDLSFTENLPDYMKLCYKIYYDIVHEVAWEVEKEQGRELVSFFRKGWEDYLLG

YYEEAEWLAAEYVPTLDEYIKNGITSIGQRILLLSGVLIMEGQLLSQEALEKVDYPGRRVLTELNSLISR

LADDTKTYKAEKARGELASSIECYMKDHPGCQEEEALNHIYGILEPAVKELTREFLKADHVPFPCKKMLF

DETRVTMVIFKDGDGFGISKLEVKDHIKECLIEPLPL

>Pa levopimaradiene/abietadiene synthase gi|44804521

MALLSSSLSSQIPTGAHHLTLNAYANTQCIPHFFSTLNAGTSAGKRSSLYLRWGKGSNKIIACVGEDSLS

APTLVKREFPPGFWKDHVIDSLTSSHKVAASDEKRIETLISEIKNMFRSMGYGDTNPSAYDTAWVARIPA

VDGSEQPEFPETLEWILQNQLKDGSWGEGFYFLAYDRILATLACIITLTLWRTGEIQVQKGIEFFKTQAG

KIEDEADSHRPSGFEIVFPAMLKEAKVLGLDLPYELPFIKQIIEKREAKLERLPTNILYALPTTLLYSLE

GLQEIVDWQKIIKLQSKDGSFLSSPASTAAVFMRTGNKKCLEFLNFVLKKFGNHVPCHYPLDLFERLWAV

DTIERLGIDRHFKEEIKDALDYVYSHWDERGIGWARENPVPDIDDTAMGLRILRLHGYNVSSDVLKTFRD

ENGEFFCFLGQTQRGVTDMLNVNRCSHVAFPGETIMEEAKTCTERYLRNALEDVGAFDKWALKKNIRGEV

EYALKYPWHRSMPRLEARSYIEHYGPNDVWLGKTMYMMPYISNEKYLELAKLDFNHVQSLHQKELRDLRR

WWTSSGFTELKFTRERVTEIYFSPASFMFEPEFATCRAVYTKTSNFTVILDDLYDAHGTLDDLKLFSDSV

KKWDLSLVDRMPQDMKICFMGFYNTFNEIAEEGRKRQGRDVLGYIRNVWEIQLEAYTKEAEWSAARYVPS

FDEYIDNASVSIALGTVVLISALFTGEILTDDVLSKIGRGSRFLQLMGLTGRLVNDTKTYEAERGQGEVA

SAVQCYMKDHPEISEEEALKHVYTVMENALDELNREFVNNREVPDSCRRLVFETARIMQLFYMDGDGLTL

SHETEIKEHVKNCLFQPVA

>Ps (-)pinene synthase gi|34582667

MALVSVAPMASRSCLHKSLSSSAHELKTICRTIPTLGMSRRGKSATPSMSMSLTTTVSDDGVQRRMGDFH

SNLWNDDFIQSLSTSYGEPSYRERAERLIGEVKKMFNSMSSEDGELISPHNDLIQRVWMVDSVERLGIER

HFKNEIKSALDYVYSYWSEKGIGCGRESVVADLNSTALGFRTLRLHGYAVSADVLNLFKDQNGQFACSPS

QTEEEIRSVLNLYRASLIAFPGEKVMEEAEIFSAKYLEESLQKISVSSLSQEIRDVLEYGWHTYLPRMEA

RNHIDVFGQDTQNSKSCINTEKLLELAKLEFNIFHSLQKRELEYLVRWWKDSGSPQMTFCRHRHVEYYTL

ASCIAFEPQHSGFRLGFAKACHIITILDDMYDTFGTVDELELFTAAMKRWDPSAADCLPEYMKGVYLILY

DTVNETSREAEKAQGRDTLDYARRAWDDYLDSYMQEAKWIATGYLPTFAEYYENGKVSSGHRTSALQPIL

TMDIPFPPHILKEVDFPSKLNDLASAILRLRGDTRCYKADRARGEEASSISCYMKDNPGATEEDALDHIN

AMISDVIRGLNWELLNPNSSVPISSKKHVFDISRAFHYGYKYRDGYSVANIETKSLVRRTVIDPVTL

>Pt (+)alpha-pinene synthase gi|28894488

MALVSAVPLNSKLCLRRTLFGFSHELKAIHSTVPNLGMCRGGKSIAPSMSMSSTTSVSNEDGVPRRIAGH

HSNLWDDDSIASLSTSYEAPSYRKRADKLIGEVKNIFDLMSVEDGVFTSPLSDLHHRLWMVDSVERLGID

RHFKDEINSALDHVYSYWTEKGIGRGRESGVTDLNSTALGLRTLRLHGYTVSSHVLDHFKNEKGQFTCSA

IQTEGEIRDVLNLFRASLIAFPGEKIMEAAEIFSTMYLKDALQKIPPSGLSQEIEYLLEFGWHTNLPRME

TRMYIDVFGEDTTFETPYLIREKLLELAKLEFNIFHSLVKRELQSLSRWWKDYGFPEITFSRHRHVEYYT

LAACIANDPKHSAFRLGFGKISHMITILDDIYDTFGTMEELKLLTAAFKRWDPSSIECLPDYMKGVYMAV

YDNINEMAREAQKIQGWDTVSYARKSWEAFIGAYIQEAKWISSGYLPTFDEYLENGKVSFGSRITTLEPM

LTLGFPLPPRILQEIDFPSKFNDLICAILRLKGDTQCYKADRARGEEASAVSCYMKDHPGITEEDAVNQV

NAMVDNLTKELNWELLRPDSGVPISYKKVAFDICRVFHYGYKYRDGFSVASIEIKNLVTRTVVETVPL

>Pt (-)alpha-pinene synthase gi|28894482

MSPVSVISLPSDLCLPTSFIDRSGRELIPLHITIPNVAMRRQGKLMTRASMSMNLRTAVSDDAVIRRRGD

FHSNLWDDDLIQSLSSPYGEPSYRERAERLIGEVKNSFNSMSNEDGESITPLDDLIQRLWMVDSVERLGI

DRHFKKEIKSALDHVYRYWSEKGIGCGRESVVTDLNSTALGLRTLRLHGYDVSADVLNHFKNQSGQFACT

LKQTEDQIRTVLNLYRASLIAFPGEKVMDEAESFSAKYLKEALQKIPVSSFSREIGDVLEYGWHTYLPRL

EARNYIDVFGQDTENSKSYMKTEKLLELAKLEFNIFHALQKRELEYLVRWWKGSGSPQMTFCRHRHVEYY

TLASCIAFEPQHSGFRLGFAKACHIITVLDDMYDTFGTLDELELFTSAIKRWDPSATECLPEYMKGVYMI

VYNTVNEMSQEADKAQGRDTLNYCRQAWEEYIDAYMQEAKWIASGEVPTFEEYYENGKVSSGHRVSALQP

ILTTDIPFPEHVLKEVDIPSQLNDLASAILRLRGDTRCYQADRARGEEASCISCYMKDNPGTTEEDALNH

LNAMISDVIKGLNWELLKPNSSVPISAKKHAFDISRAFHCGYKYRDGYSVANIETKSLVKRTVIDPVTL

>Pt alpha-terpineol synthase gi|28894486

MDLISVLPSASKSCVCLHKPLSSSTHKLKPFCKTIRILVMPRRWEFARPSMSLSTVASEDDIQRRTGGYL

SNLWNDDVIQFLSTPYGELAYRERAERLIDEVRDIFSSMSLEDGEFSDLIQRLWMVDNVERLGIDRHFKN

EIKSALDYVYSYWSEKGIGCGTKSIITNLNSTALGFRTLRLHGYPVSADVLKHFRNQIGQFVSCPSETEE

DIRIMVNLYRASLIAFPVAFPGEKVMEEAESFSEKYLKETLQKIPDCSLSREIGDVLEHGWHTNLPRLEA

RNYIDVFGQDTKNMEPNRKTEKLLELAKLEFNIFQSIQKTELESLLRWWNDSGSPQITFTRHRHVEYYTL

ASCIAFEPQHSGFRLGFAKACHILTVLDDMYDLFGTVDELKLFTAAIKRWDPSATDCLPQYMKGIYMMVY

NTVNEMSAEAQKAQGRDTLNYARQAWEDCLDSHMQEAKWIATGFLPTFEEYLENGKVSSAHRVSALQPML

TMDIPFPPHILKEVDFPSNLNDLACAMLRLRGDTRCYQADRARGEETSCISCYMKDNPGATEEDALNHLN

VMISGVIKELNWELLKPNSSVPISSKKINFDITRAFHYGYKYRDGYSVSSVETKSLVMRTLLEPVPL

>Pt alpha-farnesene synthase gi|28894484

MSSLAVDDAERRVGDYHPNLWDDALIQSLSTPYGASPYRDVAEKLIGEIKEMFASISIEDGDDEICYFLQ

RLWMIDNVERLGISRHFENEIKAAMEDVYSRHWSDKGIACGRHSVVADLNSTALAFRTLRLHGYSVCSDV

FKIFQDQKGEFACSADQTEGEIKGILNLLRASLIAFPGERILQEAEIFATTYLKEALPKIQGSRLSQEIE

YVLEYGWLTDLPRLETRNYIEVLAEEITPYFKKPCMAVEKLLKLAKIEFNLFHSLQQTELKHLSRWWKDS

GFAQLTFTRHRHVEFYTLASCIAMEPKHSAFRLGFAKLCYLGIVLDDIYDTYGKMEELELFTAAIKRWDT

STTECLPEYMKGVYMAFYDCVNEMARQAEKTQGWDTLDYARKTWEALIDAFMEEAKWISSGYVPTFQKYL

DNGKVSFGYRAATLQPILTLDIPLPLHILQEIDFPSSFNDLASSILRLRGDICGYQAERSRGEQASSISC

YMKDNPGSTEEDALSHVNAMIGDKIPEFNWEFMKPSKAPISSKKYAFDILRAFYHLYKYRDGFSIAKIET

KKLVMRTVLDPVPM

>Pa (-)limonene synthase gi|44804558

MSPVSVIPLAYKLCLPRSLMSSSREVKPLHITIPNLGMCRRGKSMAPASTSMILTAAVSDDDRVQRRRGNYHSNLWDDDFIQSLSTPYGEPSYRERAERLKGEIKKMFRSMSKDDGELITPLNDLIQRLWMVDSVQRLGIDRHFKNEIKSALDYVYSYWNEKGIGCGRDSVVADLNSTALGFRTLRLHGYNVSSEVLKVFEDQNGQFACSPSKTEGEIRSALNLYRASLIAFPGEKVMDDAEIFSSRYLKEAVQEIPDCSLSQEIAYALEYGWHTNMPRLEARNYMDVFGHPSSPWLKKNKTQYMDGEKLLELAKLEFNIFHSLQQEELQYISRWWKDSGLPKLAFSRHRHVEYYTLGSCIATDPKHRAFRLGFVKTCHLNTVLDDIYDTFGTMDEIELFTEAVRRWDPSETESLPDYMKGVYMVLYEALTEMAQEAQKTQGRDTLNYARKAWEIYLDSYIQEAKWIASGYLPTFQEYFENGKISSAYRAAALTPILTLDVPLPEYILKGIDFPSRFNDLASSFLRLRGDTRCYKADRARGEEASCISCYMKDNPGSTEEDALNHINSMINEIIKELNWELLRPDSNIPMPARKHAFDITRALHHLYKYRDGFSVATKETKSLVSRMVLEPVPL

>Pa (-)linalool synthase gi|44804547

MALLSIAPLTSTWCVDKSLVGSSEAKALLRKIPTLEMCRLTKSVTPSISMCLTTTVSDDGVQRRIADHHPNLWDDNFIQSLSTPYGATAYHERAQKLIGEVKVIINSILVEDGELITPPNDLLQRLSIVDSIERLGIDRHFKNEIKSALDYVYSYWSEKGIGCGRDSVVNDLNTTALGLRTLRLHGYPVSSDVLEQFKDQNGQFACSAIQTEGEIKTVLNLFRASLIAFPGEKVMEEAEIFSTIYLKEALLKIPVCSLSREIAYVLEYGWHMNLPRLEARNYIDVFGQDPIYLRSTQKLIELAKLEFNIFQSLQQEELKHVSRWWKDSGFSQMAFARHRHVEYYTLASCIDIYPQHSSFRLGFAKIAHLGTVLDDIYDTFGTMDELELFTAAVKRWHPSAAEGLPEYMKGVYMMFYETVNEMAREAEKSQGRDTLNYARQALEAYIDSYMKEAKWISSGFLPTFEEYLDNGKVSFGYRIATLQPILTLGIPFPHHILQEIDFPSRLNDLAGSILRLKGDIHSYQAERSRGEESSCISCYMKDNPEATEEDAVTYINAMVNRLLKELNWELLKPDNNVPITSKKHAFDILRAFYHLYKDRDGFSVARNEIRNLVMTTVIEHVPL

>Pa myrcene synthase gi|44804588

MSPVSVVPLACKLCLCRSMTSSTDELKPLPTTIPTRGMCGRRMSVTPSMSMSLNTVVSDNDAVQRRIGDYHSNLWNDDFIQSLTTPYGAPSYIERADRLISEVKEMFNRMCMEDGELMSPLNDLIQRLWTVDSVERLGIDRHFKNEIKASLDYVYSYWNEKGIGCGRQSVVTDLNSTALGLRILRQHGYTVSSEVLKVFEEENGQFACSPSQTEGEIRSFLNLYRASLIAFPGEKVMEEAQIFSSRYLKEAVQKIPVSGLSREIGDVLEYGWHTNLPRWEARNYMDVFGQDTNTSFNKNKMQYMNTEKILQLVKLEFNIFHSLQQRELQCLLRWWKESGLPQLTFARHRHVEFYTLASCIACEPKHSAFRLGFAKMCHLVTVLDDVYDTFGKMDELELFTAAVKRWDLSETERLPEYMKGLYVVVFETVNELAQEAEKTQGRNTLNYVRKAWEAYFDSYMKEAEWISTGYLPTFEEYCENGKVSSAYRVAALQPILTLDVQLPDDILKGIDFPSRFNDLASSFLRLRGDTRCYEADRARGEEASCISCYMKDNPGSTEEDALNHINAMINDIIRELNWEFLKPDSNIPMPARKHAFDITRALHHLYIYRDGFSVANKETKNLVEKTLLESMLF

>Pa (-)alpha/beta-pinene synthase gi|44804535

PRAAGKSCLHKSLSSSAHELKTICRTIPTLGMSRRGKSATPSMSMSLTTTVSDDGVQRRMGDFHSNLWNDDFIQSLSTSYGEPSYRERAERLIGEVKKMFNSMSSEDGELISPHNDLIQRVWMVDSVERLGIERHFKNEIKSALDYVYSYWSEKGIGCGRESVVADLNSTALGLRTLRLHGYAVSADVLNLFKDQNGQFACSPSQTEEEIRSVLNLYRASLIAFPGEKVMEEAEIFSAKYLEEALQKISVSSLSQEIRDVLEYGWHTYLPRMEARNHIDVFGQDTQNSKSCINTDKLLELAKLEFNIFHSLQKRELEYLVRWWKDSGSPQMTFGRHRHIEYYTLASCIAFEPQHSGFRLGFAKTCHIITILDDMYDTFGTVDELELFTAAMKRWDPSAADCLPEYMKVMYMIVYDTVNEMCQEAEKAQGRDTLDYARQAWEDYLDSYMQEAKWIATGYLPTFEEYYENGKVSSGHRVAALQPILTMDIPFPPHILKEVDFPSKLSDLACAILRLRGDTRCYKADRARGEEASSISCYMKDNPGATEEDALDHINAMISDVIRGLNWELLKPNSSVPISSKKHVFDISRAFHYGYKYRDGYSVANIETKSLVKRTVIDPVTL

>Ps pinene synthase gi|34582667

MALVSVAPMASRSCLHKSLSSSAHELKTICRTIPTLGMSRRGKSATPSMSMSLTTTVSDDGVQRRMGDFHSNLWNDDFIQSLSTSYGEPSYRERAERLIGEVKKMFNSMSSEDGELISPHNDLIQRVWMVDSVERLGIERHFKNEIKSALDYVYSYWSEKGIGCGRESVVADLNSTALGFRTLRLHGYAVSADVLNLFKDQNGQFACSPSQTEEEIRSVLNLYRASLIAFPGEKVMEEAEIFSAKYLEESLQKISVSSLSQEIRDVLEYGWHTYLPRMEARNHIDVFGQDTQNSKSCINTEKLLELAKLEFNIFHSLQKRELEYLVRWWKDSGSPQMTFCRHRHVEYYTLASCIAFEPQHSGFRLGFAKACHIITILDDMYDTFGTVDELELFTAAMKRWDPSAADCLPEYMKGVYLILYDTVNETSREAEKAQGRDTLDYARRAWDDYLDSYMQEAKWIATGYLPTFAEYYENGKVSSGHRTSALQPILTMDIPFPPHILKEVDFPSKLNDLASAILRLRGDTRCYKADRARGEEASSISCYMKDNPGATEEDALDHINAMISDVIRGLNWELLNPNSSVPISSKKHVFDISRAFHYGYKYRDGYSVANIETKSLVRRTVIDPVTL

>Pm (E)gamma-bisabolene synthase gi|59799472

MAASTLPSGLSTNDLIRRTANPHPNVWGYDLLCSLKSPYSRDSSYKERADTLINEIKAMLGAAFGDGKEMITPSAYDTAWVARIPSIDGSSGSARPQFPQTVDWILKNQLKDGSWGTESHFLLSEPLLATISCVLALFKWQVGDLQVERGIEFLKSSLEKIKNESDQDSLVTDFEIIFPSMLREAQSLHLGLPYDLPYIQLLQTKRQERLANLSREKIHGGILQLSSLEGIEDMVEWERLMDLQSLDGSFLSSPASTAFVFIHTGDLKCLAFLNSVLAKFGAFVPCLYHVDLLERLLIVDNIERLGIDRHFEKEINEALDYVYRYWSNERGIGWGRMNATADLETTALGFRLLRLHRYHVSPVVFKKFKDADGEFLSSIGQFNKDVASMLNLYRACELAFPGENILDEAKGFTAKYLREALEKTETFSSWNIKRNLSQEIKYALKTSWHASIPRVEAKRYCQVYRPDYARLDKSVYKLHHVNNEKILELAKLDFNIIQSILQEEMKNVTSWFRDSGLPLFSFARQRPLEFYFLITAGTYEPRYAKCRLLFTKVACVETVLDDMYDTYGTLDELKLFTQAVRRWDPSLTENLPDYMKRCYKIFYDIVHEAAWEAEKEQGRELVSFLRKAWEDFVLSYHEEAEWLSAEYVPGFDEYIKNGITSIGQRVLLLSGLLVMDGQLLSQKALEKIDYPERSRVLMEQICLISRLADDTQSYKAEKARGELASGIECYMKDHPECTEEEALNHIYGIMEVTAKELTKEYLKVDDDDVPFACKKMLFEETRVTMVIFKDGDRLSNSKLEMKDHFKECLIEPLPL

>Pm (E)beta-farnesene synthase gi|59799470

MSLEEFYPMATVYVPSSTLPCALSTSSSSSSLVRRTANPHPNVWDYHFVQSLQSPYTDPCYGERVETLVAEIKAMLHGEGGLMITPSAYDTAWVARVPSIDGSARPQFPQTVQWILKNQLKDGSWGTESHFLLSDRLLATLSCVLALLKWKVGDLQVQQGIEFIKSNLEAIKDENDEDSLVTDFDIIFPSLLREAQYLDIELPLQPALCKSTPPKRQERLANMSREEIHGVPSPLLYSLEGIEDMVDWERIMDVRSQDGSFLSSPASIACVFMHTGDIKCLEFLNNVLTNFGTFVPCLYPVDLLERLLIVDNLVQLGIDRHFEKEIKEALDYVHRHWNERGIGWGRLNPIADLEITALGFRLLRLHRYNVSPAVFENFKDSNGHFVCSGAQFNKDVASMLSLYRASQLAFPGENILDEAKSFTSKYLKEALEKRETYSAWNNKQSLSEEIKYALENSWHASVPRVEAKRYCQVYRSDYTYLAKSVYKLPKVNNEKILELAKLDFQHYPGHPPKRDEECHHLVKNSEFPLLPFGRERPVECFFIVAAGTYEPQYAKCRFLFSKVACLNTVLDDMYDTYGTLDELKLFTEAVRRWDLSLTENLPDYMKLCYKIFYDIVHEVVLEAEKEQGRELLTFFRKGWEEYLMGYYEEAEWLACEYLPSLEEYIRNGIISIGQRILVVSGVLLMEGQILSQEALEQLDYPGRRVLTELNSIITRLADDIHTYKAEKARGELASSIECYMREHPGSTEEVAVNYMYSLLEPAVKELTWEFLKPEDSTVHIPFQCKKMLMEETRVTMVIFKEGDGFGISKTKIKDYIKDCLIEPLPL

>Pb_Contig235

GKGLYMAVYDNINEMAQEAQRIQGRDTVSYVRKSWEALIDAHMQEAKWISSGYLPTFEEYLENGKVSFGSRLTTLEPMLTLGFPLPPRTLQEIDFPSNFNDLICAILRLRGDTQCYKADRARGEEASSVSCYMKDHPGITEEDAVNQINAMVNNLTKELNWELLRPDSGVPISYKKFSFDIWRVFHYGYKYRDGFSVASIELKNLVTRTVVETVPL*

>Pb_Contig2734

GGTLDELKLFTEAVRRWDLSYTENLPDYMKLCYKIYYDIVHEVAWEAEKEQGRELVSFFRKGWEDYLLGYYEEAEWLAAEYVPSLDEYIKNGITSIGQRILLLSGVLIMEEQLLSQEALEKVDYPGRRVLTELNSLISRLADDTKTYKAEKARGELASSIECYMKDHPECTEEEALNHIYGILEPAVKELTKEFLKPGDDDVPFACRKMLFEETRVTMVIFKDGDGFGVSKWEVKDHITECLIDPIPL

>Pb_Contig3962

GENASVSIALGTVVLISALFTGEILTDDILSKIGRDSRFLYPMDLTGRLVNDTKTYQAERGQGEVASAVQCYMKDHPEISEEEALKHVYTIMDNALDELNREFVNNRDVPDTCRRLVFETARIMQLFYMDGDGLTLSHNMEIKEHVKNCLFQPVA*

>Pb_Contig625

GGTLIDFSVHYFQLLEAGMSLISAVPLASSCVSKSLISSVREHKALRRAIATLQMSRPGKSVAASTRMSSATAGCDDGVKRRIGDYHSNLWDDNFIQSLSSPYGASSYGDHADRLIGEVKEIFNSFSIADGELTSPVSDLLQQLWMVDNVERLGIDRHFQTEIKVALDNVYRYWSEKGIGCGRDSASTDLNTTALGFRIFRLHGYTVSSDAFEHFKDQMGQFTASANDTELQTRSVFNLFRASLIAFPEEKVLEEAEKFAAAYLKAALQTLPVSGLSREIKYVFDYRWHSNLPRLEARSYIDILADNTISGTPDANTKKLLELAKLEFNIFHSLQQKELQCLWRWWKEWGCPELTFIRHRYVEFYTLVSGIDMVPEHATFRLSFVKTCHLITILDDMYDTFGTIDELRLFTAAVKRWDPSATECLPEYMKGVYMVLYETVNEMAKEAQKSQGRDTLGYVRQALEDYIGSYLKEAEWIATGYVPTFQEYFENGKLSSGHRIATLQPILTLSIPFPHHILQEIDFPSKFNDYACSILRLRGDTRCYKADSARGEEASCTSCYMKENLGSTQEDALNHINGMIEDLIKKLNWEFLRPDNNAPISSKKHAFNISRGLHHFYNYRDGYSVASNETKDLVIKTVLEPVLM*

>Pb_Contig7348

GNEIAEEGRKRQGHDVLPYIRNLWEIQLESFAKEAEWSRAEHVPSFHEYIKAAAISSALPTLVLIGVIFTGEVLTDHILSQIDYRSKFAYLMSLTGRLANDTKTYQVERGQGEVASAIQCYMKENPELSEEEALEYIYRLMENALADFKCEFLDTKDVPEYCRRLVFDNARSMQLIYMEGDGFKLSHETEIKEHVKKILFEPVA*

>Pc_Contig_3056

GDVLGYIRNLWEIQIEAFTKEAEWAESEYVPSFQEYIETATVSVALATMVLITILFTGEVLTDHILSQIDYRSKFAYLMCLTARLINDTKTYQAERSRGEVASAVQCYMKDHPELSEGEALKHVYTLMEDALTNLKVEFLKAKDVPDKCRRLVFDNARMMQLFYQQGDGLTLANDTEIKQHVKNILFQPVP*

>Pb_Contig8905

YALVLHSYRVYCVHRWWSQSDIEKQNFYRKRHVEFYFWMVIGTFEPEFSGSRIAFAKIATLMTILDDLYDTHGTLEQLKIFTEAVKRWDLSLQDRLPDYIKITLQFFFNTSNELNAEVAKMQERDMSAYIRKAGWERYLEGYMQESEWMAARHVPTFDDYMKNGKPSSGMCILNLYSLLLMGRLVPDNILEQIYLPSKIHELVELTARLVDDSKDFQAKKNGGEFASGIECFLKEKPECTEEDAMNHLIGLLNLKAMELNWEFVKHDGVEQCLKKFVFEVARGLRFIYKYRDGFDYSNKEMKTQITKILIDHVPI*

>Pc_Contig4969

MKDSCSDAADDLIRRLQMVDIIECLGIDRHFQPEIKEAIDYVYRYWNETGIGLGSRNSGIKHLYSTALGFRALRMHRYNVSSDVLENFKDENGQFFCSSSGEEGNADKEVRSMLSLFRASNISFPGEKVMEEAKTFTTQYLTQVLTGHAVADVDQSLQREVKYALEFPWHCSVPRWEARNFIEIYEQNYSWLKSSINQKIL??AKLDFNILQCTHKKEMQLISRWWSESYLPQLDFYRKRHVELYFWAVLGAFEPEFRSSRIAFTKLSTVMTVIDDLYDTHGTLDEIKIFTEGVRRWDTSLISRLPDHIQKIFEFFMKTSNEWTAEVEKKQGRDMAAYIRKNAWERYVESYLQEAEWMAAGYVPSFNEFYKNGLASSGMCVLNLIPLLLMDQILPDDILKQIVYPSKIHDLLELTIRVKDDIMDFEKEKEHGEVASTIECYLKDNPACTCEDALNHMKGILDLSVSQLNWEFLKHDNVPLCCKRFTFNLARGMHFLFKYNDGITLTDNEVKDQIFKVLIQPLQL*

>Pc_PCO0136.CR_F03

FFMKTSNEWTAEVEKKQGRDMAAYIRKNAWERYVESYLQEAEWMAAGYLPSFNEYYKNGLASSGMCVLNMIPLLLMDQILPDDILKQIVYPSKIHDLLELTVRVKDDITDFEQEKERGEVASSLECYVKDNPERTREDALNHMKGILDLSVSQLNWEFLKHDNVPLCCKRFTFNLARGMHFLFKYKDGISLSDNEVKDQIFKVLIQPLQL

>Pc_Contig5096

MKTSNEWTAEAEKKQGRDMAAYIRKNAWERYVESNLQEAEWMAAGYVPSYNEYYKNGLASSGMCVLNLIPLLLMDQILPDDILKQIVYPSKIHDLLELTVRLKDDITDFEQEKEHGEVASSIECYVKDNPECTCENALNHMKGILDLSVSQLNWEFLKHDNVPLCCKRFTFNLARGMHFLYKYNDGISLSDNEVKDQIFKVLIQPLQL

>Pb_Contig625

GGTLIDFSVHYFQLLEAGMSLISAVPLASSCVSKSLISSVREHKALRRAIATLQMSRPGKSVAASTRMSSATAGCDDGVKRRIGDYHSNLWDDNFIQSLSSPYGASSYGDHADRLIGEVKEIFNSFSIADGELTSPVSDLLQQLWMVDNVERLGIDRHFQTEIKVALDNVYRYWSEKGIGCGRDSASTDLNTTALGFRIFRLHGYTVSSDAFEHFKDQMGQFTASANDTELQTRSVFNLFRASLIAFPEEKVLEEAEKFAAAYLKAALQTLPVSGLSREIKYVFDYRWHSNLPRLEARSYIDILADNTISGTPDANTKKLLELAKLEFNIFHSLQQKELQCLWRWWKEWGCPELTFIRHRYVEFYTLVSGIDMVPEHATFRLSFVKTCHLITILDDMYDTFGTIDELRLFTAAVKRWDPSATECLPEYMKGVYMVLYETVNEMAKEAQKSQGRDTLGYVRQALEDYIGSYLKEAEWIATGYVPTFQEYFENGKLSSGHRIATLQPILTLSIPFPHHILQEIDFPSKFNDYACSILRLRGDTRCYKADSARGEEASCTSCYMKENLGSTQEDALNHINGMIEDLIKKLNWEFLRPDNNAPISSKKHAFNISRGLHHFYNYRDGYSVASNETKDLVIKTVLEPVLM*

>Pb_Contig8298

FVKFYLCVEVAAMDLISVLPSASKSCVCLHKPLSSSTHKLKPFCKTIRILGMPRRWKFAGPSMSLSTVASDDDIQRRTGGYHSNLWNDDVIQFLSTPYGELAYRERAERLIDEVRDIFSSMSLEDGEFSDLIQRLWMVDNVERLGIDRHFKNEIKSALDYVYSYWSQKGIGCGTKSIITNLNSTALGFRTLRLHGYPVSADVLKHFRNQIGQFVSCPSETEEDIRSMVNLYRASLIAFPGEEAMEEAESFSEKYLKETLQKIPDCSLSREIGDVLEHGWHTNLPRFEARNYIDVFGQDTKNMESNRKAEKLLELAKLEFNIFQSIQKTELESLLRWWNDSGSPQITFTRHRHVEYYTLASCIAFEPQHSGFRLGFAKACHIITVLDDMYDLFGTVDELKLFTAAIKRWDPSATDCLPQYMKGIYMMVYNTVNEMSAEAQKAQGRDTLNYARQAWEVYLDSYMQEAKWIATGYLPTFEEYLENGKVSSGHRVSALQPMLTMDIPFPPHILKEVDFPSNLNDLACAILRLRGDTRCYQEDRARGEETSCISCYMKDNPGATEEDALNHLNVMISGVIKGLNWELLKPDSGVPISSKKINFDITRAFHYGYKYRDGYSVSSVETKSLVMRTLLEPVPL*

>Pc_Contig540

AGSPQITFTRHRHVEYYTLASCIAFEPQHSG?RLGFAKACHIITVLDDMYDLFGTVEELKLFTAAIKRWDPSATDCLPQYMKGIYMMVYNTVNEMSAEAQKAQGRDTLNYARQAWEVYLDSYMQEAKWIATGYLPTFQEYLENGKVSSGHRVSALQPMLTMDIPFPPHILKEVDFPSNLNDLACAILRLRGDTRCYQEDRARGEETSCISCYMKDNPGATEEDALNHLNVMISGVIKELNWELLKPDSSVPISSKKINFDITRAFHYGYKYRDGYSVSSVETKSLVMRTLLEPVPL

>Pb_Contig235

GKGLYMAVYDNINEMAQEAQRIQGRDTVSYVRKSWEALIDAHMQEAKWISSGYLPTFEEYLENGKVSFGSRLTTLEPMLTLGFPLPPRTLQEIDFPSNFNDLICAILRLRGDTQCYKADRARGEEASSVSCYMKDHPGITEEDAVNQINAMVNNLTKELNWELLRPDSGVPISYKKFSFDIWRVFHYGYKYRDGFSVASIELKNLVTRTVVETVPL*

>Pc_Contig4556

AQEIEYLLEYGWHTNLPRLEARMYMDVFPQDTIYEQKLVELAKVEFNIFHSLQKRELQSLTRWWKHYGFPQLSFTRHIHVEYYTFASCVATDPKQSAFRLGFAKMSHFVTVLDDIYDTYGTMEELELFTAAIKRWDPSLVDCLPEYMKGVYMAVYDTVNEMAKEAEKVQGRDTLNYVRQAWEPYFDAYMIEAKWISSGYLPTLQEYLDNSKISFGSRITILQPILTLGEPLPHEILQEIDFPSKFNDLISVLLRLKGDTRCYKADRARGEEASSVSCYMKDNAGLTEEDAIHRINAMVHNLLKELNWELLKPDCNVPISCKKAAFDICRIFHHGYKYRDGYGDATIETKNLVKRTVLEPVPL

>Pb_Contig3891

GDLDSYMKEAEWISTGCLPTFEEYYENGKISFGYRISMLQPILSMDIPFPHHILQEIDYPSRFSSLAAGILRLKGDTRCYQADSARGEEASCISCYMKENPGLTEEDVVNHIHGMVDDLIKELNWELLKPDCNVPISSKKHAFDICRAVHHGYKYRDGYSVATNEIKDLVMITVLEPVPL*
