# Supplementary material for: Influence of water deficit on the molecular responses of Pinus contorta × Pinus banksiana mature trees to infection by the mountain pine beetle fungal associate, Grosmannia clavigera
Source: Tree Physiol. 2013 Dec 5;34(11):1220–39. doi: 10.1093/treephys/tpt101 (PMC4277265; doi:10.1093/treephys/tpt101)
Supplement: Supplementary Data [file supp_tpt101_tpt101supp_table1.docx]

**Supplemental Table S1.** Gene name, EST pine database and sequences of primers used for qRT-PCR

| **Designated gene name** | **Clone ID** | **Forward primer** | **Reverse primer** |
| --- | --- | --- | --- |
| ***PcbTIP2;1*** | PCO0139_K24 | TGGAATACTGAGAAAGAAAAGGTTGA | CAGTGGAGAAATATCTAGACGACTGAA |
| ***PcbTIP1;1*** | PCO0120_P03 | GGGCATTTCCACGCTCAT | AAAGCCATGCCGGATCCT |
| ***PcbPIP2;1*** | PCO0132_K04 | GACGAGGGAAGTCTAGATAAGCAAA | CTCAATCATGAAAACAGCTTCCAT |
| ***PcbPIP1;1*** | PCO0126_B15 | TCTGGGTTGGTACCCTTGGT | GGCTCGGATGATGAGCATGT |
| ***PcbTINY-like2*** | PCO0125_L21 | TGCATCAATATCTGCAGAAACTTCT | AATTCCAATTGAAGCCGTTGAAT |
| ***PcbTINY-like1*** | PCO0125_O07 | ACAGTGCTTCTGATAATTTCTATGATGTAA | CCTGTTGGATCCGACCATATGT |
| ***PcbDREB7-like*** | PCO0140_F24 | GGAAAAACTGTGATACAGAATGGAAAATA | GCAATCAGTATAATAACAGAAATCAGGAAT |
| ***PcbRAP2.4-like*** | PCO023_F17 | TGCTTGTACTAGTGTAAAGTTAGCATTCAGTA | CAGAGGAAGATGATATTTATTTATCTCTCTGAT |
| ***PcbERF61-like*** | PCO0211_P16 | ATTTCAAAGTGTCTGGCTGGATTAT | CAATGGATGTAGCTGATCAATAGGTT |
| ***Pcb (E)-beta-farnesene synthase*** | PCO0133_B13 | TGAGACCTGCCTTCCAGATGA | CTTCCGTGAAAGCTCAAATAGAAAC |
| ***Pcb diterpene synthase-like*** | PCO0215_B23 | GGAGTTGTTGCTTAGTATTGGTATTTGT | CTGTTTGATGGCAATGTCTCTTTAG |
| ***Pcb (+)-alpha-pinene synthase-like*** | PCO026_F07 | TTCCTAAGTTTCTGATTTCGCTACCTA | CTCCTCCATATAATGATATGGATGTTTTT |
| ***Pcb (+)-3-carene synthase-like*** | PCO029_K24 | CCTAGTCTACGTTACTTTAGGTTATGTGTTTT | AACCTAGGCCTATACGACCAAAGA |
| ***PcbCHI4.1*** | PCO0124_O12 | TGGTGCTAGTCCTGCTACTGGC | AGATCCTCCTCCTGAACTGGTACAA |
| ***PcbCHI2.2*** | PCO016_G11 | GTCAGCCACCGATACCGCT | GGCCCACCGTTTATGATGTC |
| ***PcbCHI2.1*** | PCO024_G13 | CCGGATCTCTTGGTGACTGAC | GGCGGCAGTATCGTCGTT |
| ***PcbCHI1*** | PCO0111_M10 | GAGATTTCGGATTAGCAGCAGTTC | GGTATGGATTTTATTATATCTTATTATGGCAA |
| ***PcbCHIi5.1*** | PCO024_O22 | CTGTGTCATGGAACCAAGCG | GCTACAGATTCCTGCGTTTTATCATT |
