# Supplementary material for: Influence of water deficit on the molecular responses of Pinus contorta × Pinus banksiana mature trees to infection by the mountain pine beetle fungal associate, Grosmannia clavigera
Source: Tree Physiol. 2013 Dec 5;34(11):1220–39. doi: 10.1093/treephys/tpt101 (PMC4277265; doi:10.1093/treephys/tpt101)
Supplement: Supplementary Data [file supp_tpt101_tpt101supp_table2.docx]

**Supplemental Table S2**. The table shows the characteristics of the major chitinase classes (Glycosyl hydrolase family 19) according to the domain structure as shown by Hamel et al. (1997).

| **Domain^1^** | **Class** | | | ***Pinus contorta* analyzed chitinases** | | | |
| --- | --- | --- | --- | --- | --- | --- | --- |
|  | **I** | **II** | **IV** | ***PcCHI1.1*** | ***PcCHI4.1*** | ***PcCHI2.1*** | ***PcCHI2.2*** |
| **Signal peptide** | Present | Present | Present | Present | Present | Present | Present |
| **Chiting-binding** | Present | Absent | Present | Present | Present | Absent | Absent |
| **Catalytic region** | Present | Present | Present | Present | Present | Present | Present |
| **Carboxyl-terminal extensión** | Present or absent | Absent | Absent | Present | Absent | Absent | Absent |
| **One deletion in the catalytic region: 14 amino acids** | Absent | Present or absent | Absent | Absent | Absent | Present^2^ | Present^2^ |
| **Two deletions in the catalytic region: 13 and 19 amino acids** | Absent | Absent | Present | Absent | Present^3^ | Absent | Absent |

^1^Domains according to Hamel et al., (1997).

^2^Deletions are 17 and 16 amino acids.

^3^The two deletions are 13 amino acids.
